# Association between Usual Dietary Intake of Food Groups and DNA Methylation and Effect Modification by Metabotype in the KORA FF4 Cohort

**DOI:** 10.3390/life12071064

**Published:** 2022-07-15

**Authors:** Fabian Hellbach, Sebastian-Edgar Baumeister, Rory Wilson, Nina Wawro, Chetana Dahal, Dennis Freuer, Hans Hauner, Annette Peters, Juliane Winkelmann, Lars Schwettmann, Wolfgang Rathmann, Florian Kronenberg, Wolfgang Koenig, Christa Meisinger, Melanie Waldenberger, Jakob Linseisen

**Affiliations:** 1 Institute for Medical Information Processing, Biometry and Epidemiology, Medical Faculty, Ludwig-Maximilian University of Munich, Marchioninistr. 15, 81377 Munich, Germany; nina.wawro@helmholtz-muenchen.de (N.W.); jakob.linseisen@med.uni-augsburg.de (J.L.); 2 Epidemiology, Faculty of Medicine, University Hospital Augsburg, University of Augsburg, Stenglinstraße 2, 86156 Augsburg, Germany; chetana.dahal@helmholtz-muenchen.de (C.D.); dennis.freuer@med.uni-augsburg.de (D.F.); christine.meisinger@med.uni-augsburg.de (C.M.); 3 Institute of Health Services Research in Dentistry, Medical Faculty, University of Münster, Albert-Schweitzer-Campus 1, 48149 Münster, Germany; sebastian.baumeister@uni-muenster.de; 4 Institute of Epidemiology, Helmholtz Zentrum München, German Research Center for Environmental Health (GmbH), Ingolstädter Landstr. 1, 85764 Neuherberg, Germany; rory.wilson@helmholtz-muenchen.de (R.W.); peters@helmholtz-muenchen.de (A.P.); waldenberger@helmholtz-muenchen.de (M.W.); 5 Research Unit Molecular Epidemiology, Helmholtz Zentrum München, German Research Center for Environmental Health (GmbH), Ingolstädter Landstr. 1, 85764 Neuherberg, Germany; 6 Else Kröner-Fresenius-Center for Nutritional Medicine, TUM School of Life Sciences, Technical University of Munich, 85354 Freising, Germany; hans.hauner@tum.de; 7 Institute of Nutritional Medicine, School of Medicine, Technical University of Munich, Georg-Brauchle-Ring 62, 80992 Munich, Germany; 8 German Center for Diabetes Research (DZD e.V.), Ingolstädter Landstr. 1, 85764 Neuherberg, Germany; 9 Institute for Biometrics and Epidemiology, German Diabetes Center, Leibniz Center for Diabetes Research at Heinrich Heine University Düsseldorf, Auf’m Hennekamp 65, 40225 Düsseldorf, Germany; rathmann@ddz.de; 10 Institute of Neurogenomic, Helmholtz Zentrum München, German Research Center for Environmental Health (GmbH), Ingolstädter Landstr. 1, 85764 Neuherberg, Germany; juliane.winkelmann@helmholtz-muenchen.de; 11 Institute of Health Economics and Health Care Management, Helmholtz Zentrum München, German Research Center for Environmental Health (GmbH), Ingolstädter Landstr. 1, 85764 Neuherberg, Germany; lars.schwettmann@helmholtz-muenchen.de; 12 Department of Economics, Martin Luther University Halle-Wittenberg, 06099 Halle, Germany; 13 Department of Genetics and Pharmacology, Institute of Genetic Epidemiology, Medical University of Innsbruck, Schöpfstr. 41, 6020 Innsbruck, Austria; florian.kronenberg@helmholtz-muenchen.de; 14 DZHK (German Centre for Cardiovascular Research), Partner Site Munich Heart Alliance, Pettenkoferstr. 8A & 9, 80336 Munich, Germany; koenig@dhm.mhn.de; 15 German Heart Centre Munich, Technical University Munich, Lazarettstr. 36, 80636 Munich, Germany; 16 Institute of Epidemiology and Medical Biometry, University of Ulm, Helmholtzstr. 22, 89081 Ulm, Germany

**Keywords:** humans, diet, metabotype, interaction, EWAS, EPIC, epigenome-wide association study

## Abstract

Associations between diet and DNA methylation may vary among subjects with different metabolic states, which can be captured by clustering populations in metabolically homogenous subgroups, called metabotypes. Our aim was to examine the relationship between habitual consumption of various food groups and DNA methylation as well as to test for effect modification by metabotype. A cross-sectional analysis of participants (median age 58 years) of the population-based prospective KORA FF4 study, habitual dietary intake was modeled based on repeated 24-h diet recalls and a food frequency questionnaire. DNA methylation was measured using the Infinium MethylationEPIC BeadChip providing data on >850,000 sites in this epigenome-wide association study (EWAS). Three metabotype clusters were identified using four standard clinical parameters and BMI. Regression models were used to associate diet and DNA methylation, and to test for effect modification. Few significant signals were identified in the basic analysis while many significant signals were observed in models including food group-metabotype interaction terms. Most findings refer to interactions of food intake with metabotype 3, which is the metabotype with the most unfavorable metabolic profile. This research highlights the importance of the metabolic characteristics of subjects when identifying associations between diet and white blood cell DNA methylation in EWAS.

## 1. Introduction

Epigenetic modifications represent a possible link between dietary intake and disease risk. Several studies support the idea that diet may be actively involved in epigenetic regulation which eventually impacts the development of chronic diseases, including cardiometabolic diseases [1]. Rather than focusing on nutrients, analyzing food groups and dietary patterns is more appropriate, as the results are easier to translate to public health recommendations. DNA methylation is one of the epigenetic regulatory mechanisms that can affect gene expression in one of two ways, either by enhancing or suppressing gene expression, for example by enhancing the binding capacity of transcription factors [2]. About 1% of all nucleic acids in the human genome are methylated cytosines, most of which are preceded by a guanine base, called CpG sites. Enriched CpG regions, called CpG islands (CGIs), are roughly 1000 base pairs long with a higher CpG density than the remaining genome [2].

Epigenome-wide association studies (EWAS) hypothesized a role of folic acid, vitamin B12 and methyl group donors in DNA methylation pattern trajectories [3]. So far, however, study results are not consistent with this theory [4,5], instead supporting the idea that the supply of methyl groups (methionine, betaine, choline) or vitamins involved in the C1 pathway are not a major determinant of CpG site methylation. Another hypothesis refers to the impact of diet on systemic inflammation, acknowledging that inflammatory processes themselves can lead to disturbance in the balance of DNA methylation patterns [3] and therefore might be a pathway of altering DNA methylation through diet. Dietary EWAS mostly focused on nutrients and more recent work analyzed dietary patterns [6]. Only a few EWAS analyzing food groups have so far been performed [7], which leaves a gap to be filled. Differential DNA methylation is strongly associated with metabolic derangements such as cancer or obesity [3,8]. Including information about metabolic status into a diet-DNA methylation analysis can give valuable insight about effect modification by metabolic profiles [9]. The estimation of clusters of subjects with homogenous metabolic characteristics within each cluster (also called subgroup) is a possible approach. Pooling multiple metabolic characteristics into one cluster takes the wide facets of interactions between them into account and thus qualifies as a suitable solution to test for metabolic effect modification. Based on a few standard clinical parameters, the definition of so-called metabotypes by the k-means procedure has been described by both our group and others [9,10]. We hypothesize that there will be different methylation trajectories in reaction to usual dietary intake in distinct metabolic situations as characterized by metabotypes.

In this cross-sectional exploratory analysis of participants in the population-based KORA FF4 study using data from the Infinium MethylationEPIC BeadChip array, our primary research goal was to examine the effect modification of usual dietary food group intake by metabotype on DNA methylation. Further analysis included examining the basic association of food groups with DNA methylation. Various food groups were analyzed, with a particular focus on food groups that provide the nutrients involved in C1 metabolism or inflammatory processes.

## 2. Materials and Methods

The Strengthening the Reporting of Observational Studies in Epidemiology—Nutritional Epidemiology (STROBE-nut) checklist was used to report the findings of the present study [11].

### 2.1. Subjects

The Cooperative Health Research in the Augsburg Region (KORA) FF4 study is the second follow-up of the population-based health survey KORA S4 conducted in the city of Augsburg and two surrounding counties in Germany between 1999 and 2001. Four-thousand, two-hundred and sixty-one randomly selected subjects aged 25–74 years agreed to participate in the S4 baseline study and 2279 of them participated in the FF4 follow-up study in 2013/2014. Details regarding the participation procedures are published elsewhere [12]. Methylation data were available for 1928 KORA FF4 participants—1888 after removing outliers. We excluded cases without nutrition data (541 participants), existing blood disorders (including hematologic cancers, four participants) and participants with very high or very low caloric intake (≤500 kcal; ≥5000 kcal per day, 0 participants). A final count of 1261 participants had full information on all covariates and were included in the EWAS. The investigation was conducted according to the guidelines laid down in the Declaration of Helsinki, including written informed consent of all participants. All study methods involving human subjects were approved by the ethics committee of the Bavarian Chamber of Physicians, Munich (EC No. 06068).

### 2.2. Habitual Dietary Intake

Dietary data were collected via repeated 24-h food lists and food frequency questionnaires (FFQ) with 246 and 148 items, respectively. The 24-h food list was developed for the German National Cohort [13] and participants were asked to report food intake of the past day via web-based forms. The FFQ was based on the German version of the multilingual European Food Propensity Questionnaire and also a web-based form [14]. Usual dietary intake of food items was modeled with the probability of consumption for each subject from at least two non-consecutive 24-h food lists (FFQ was used as a covariate) times the amount consumed, if consumed. Consumption amount was estimated from the Bavarian consumption study II, adjusting for age, sex, BMI, physical activity, and smoking status. This was made to reduce measurement error, which is prominent in dietary data. Supplement intake was not considered for computation of usual dietary intake. Further information on the usual dietary intake calculation is provided elsewhere [15].

The dietary data were categorized into 17 main food groups and 71 subgroups in accordance with the EPIC SOFT classification scheme [16]. Nutrient data were calculated based on the German Nutrient Database (Bundeslebensmittelschlüssel), version 3.01 [17].

We used the residual method to obtain a value for each food group independent of total energy intake [18]. We added the predicted food intake for the mean energy intake of the study population to the residuals for better interpretability. Additionally, we calculated two slightly modified dietary patterns using the usual dietary intake data: Alternate Healthy Eating Index 2010 (AHEI-2010) [19] and the Mediterranean Diet Score (MDS) [20]. The Alternate Healthy Eating index 2010 (AHEI) is a score, which assesses consumption of foods and nutrients predictive of chronic disease risk (e.g., vegetables, fruit, alcohol). A higher score is associated with lower risk of chronic disease risk, with major importance to public health. We had to exclude trans-fats for the AHEI, since these data were not available for the KORA FF4 study, resulting in a maximum of 100 points instead of 110. Since the scoring of the AHEI is based on servings, we transformed our usual dietary intakes from grams/day to servings/day with reported references [19]. The MDS is associated with high adherence to a dietary pattern followed by people living in Mediterranean countries, which emphasizes the consumption of cereals, fish, vegetables, legumes, fruit and nuts, and a high ratio of unsaturated to saturated lipids. The modification of the MDS calculates the fat ratio as a sum of monounsaturated and polyunsaturated fatty acids divided by saturated fatty acids. The MDS is a population-specific dietary score, meaning that the MDS scores reflect the individual consumption relative to the sex-specific population median of the respective food group, except for alcohol, where a moderate amount of consumption is scored as ideal.

### 2.3. Metabotype

Dahal et al. ([10] unpublished) developed a metabotype cluster solution based on glucose, HDL-cholesterol, non-HDL-cholesterol, uric acid and BMI by applying machine-learning methods. Parameter selection was computed based on 14 variables. To select the most fitting parameters for metabotyping, permutation variable importance was applied. It is based on the random forest method to identify the most important variables. To validate the results, two additional methods were applied. First, cross-validated permutation importance measure was applied, which is an average of all k-fold-cross-validation permutation importance. Second, gradient-boosted feature selection was used, which is a boosted tree-based supervised learning algorithm. In this method, importance scores are given to each predictor based on how many times it has been chosen to make a major decision in a given decision tree and averaging these important scores across all decision trees in the end. Finally, metabotype clusters were built by k-means clustering, and a three cluster solution was chosen as the most appropriate, with metabotype 1 inheriting the most favorable and metabotype 3 inheriting the most unfavorable metabolic parameters, while metabotype 2 is in between.

### 2.4. DNA Methylation Data

Genomic DNA (750 ng) from 1928 individuals was bisulfite-converted using the EZ-96 DNA Methylation Kit (Zymo Research, Orange, CA, USA) in two separate batches (N = 488, N = 1440). Subsequent methylation analysis was performed on an Illumina (San Diego, CA, USA) iScan platform using the Infinium MethylationEPIC BeadChip according to standard protocols provided by Illumina. GenomeStudio software version 2011.1 with Methylation Module version 1.9.0 was used for initial quality control of assay performance and for generation of methylation data export files.

Further quality control and preprocessing of the data were performed in R v3.5.1 with the package minfi v1.28.3 and primarily following the CPACOR pipeline [21]. Raw intensities were read into R (command read.metharray) and background corrected (bgcorrect.illumina). Probes with detection *p*-values > 0.01 were set to missing.

Before normalization, problematic samples and probes were removed. Forty samples were removed from the data set: Two samples showed a mismatch between reported sex and that predicted by minfi; 33 had a median intensity <50% of the experiment-wide mean, or <2000 arbitrary units; and nine (four overlapped with previous) had >5% missing values on the autosomes. A total of 59,631 probes were removed (some overlapping multiple categories): cross-reactive probes as given in published lists (N = 44,493) [22,23], probes with SNPs with minor allele frequency > 5% at the CG position (N = 11,370) or the single base extension (N = 5597) as given by minfi, and 5786 with >5% missing values. Finally, probes from the X chromosome (N = 17,743, following quality control) and the Y chromosome (N = 379) were excluded from the analysis. A total of 788,106 probes remained for analysis.

Quantile normalization (QN) was then performed separately on the signal intensities divided into the six probe types: type II red, type II green, type I green unmethylated, type I green methylated, type I red unmethylated, type I red methylated [21]. For the autosomes, QN was performed for all samples together; for the X and Y chromosomes, males and females were processed separately. The transformed intensities were then used to generate methylation beta values, a measure from zero to one indicating the percentage of cells methylated at a given locus. We checked the beta values for outliers with ±3* interquartile range and excluded these data points (40 of 1928 were excluded).

For mapping the probes to genes, we used the Infinium MethylationEPIC Manifest file genome build 37 (available at www.illumina.com via product files, accessed on 14 April 2022), which uses the gene database of the University of California Santa Cruz. Informed consent for genetic studies was obtained from all subjects.

### 2.5. Statistical Analysis

We performed linear regression analysis to explore the association of food intake and DNA methylation and effect modification by metabotype. Therefore, effect size estimates can be read as mean %-methylation change per gram residual intake. The alpha threshold was set at 6.34 × 10^−8^ (Bonferroni—basic model) and at 0.1 (False Discovery Rate (FDR)—interaction model). We chose the FDR correction in the main analysis (effect modification) because of the explorative nature we had in mind when planning this analysis. The FDR correction comes with increased power compared to other adjustment methods [24]. In total, we tested 37 food groups, nutrients and diet quality scores: potatoes, total vegetables, leafy vegetables, fruit vegetables, root vegetables, cruciferous vegetables, mushrooms, onions and garlic, legumes, total fruit, nuts and seeds, milk, yogurt, cheese, cream, grain products, whole grain products, total meat, fresh red meat, processed meat, total fish, eggs, plant oils, butter, margarine, total sweets, cakes, sugar sweetened beverages, coffee, tea, wine, beer, spirits, alcohol, AHEI, MDS and folic acid. Methylation beta values were regarded as the dependent variable. Food group intakes (g/day, continuous), dietary pattern scores (integer) and folic acid intake (µg/day continuous) were used as exposures. Alcohol in g/day and hs-CRP in mg/L were tested in addition to validate our data, as both are known to significantly impact DNA methylation [25,26]. Covariate selection was based on the literature and our own assessment of confounding with the disjunctive cause criterion [27]. The covariates selected for the model were sex, BMI (continuous), BMI squared, age (continuous), age squared, total caloric intake (continuous), alcohol in g/day (continuous—not included in the model for wine, beer, spirits, AHEI and MDS), metabotype (categorical variable), smoking behavior (regular, former, never), measured cell counts (monocytes, basophiles, eosinophils and lymphocytes) and plate as a technical variable. An interaction term for exposure and metabotype was added to the model in the interaction analysis. Relevant *p*-values were the ones for the interaction terms. Marginal effect sizes, standard errors for metabotype 2 and 3 were calculated based on emtrends() function in the emmeans package and *p*-values were calculated using the t-distribution and *t*-values. Marginal effect sizes and standard errors are shown in tables and plots for the interaction analysis. Examination of multicolinearity of covariates was made by a correlation matrix and neutrophil granulocytes were excluded as a covariate. We accounted for genomic inflation in a sensitivity analysis for the most prominent food groups in the interaction analysis, in terms of significant signals, with the bacon package [28]. An additional sensitivity analysis, also for the most prominent food groups, included leisure time physical activity (active and not active, assessed by means of a questionnaire, see [29]) and menopausal status (≤50 years of age and >50 years of age, see [30]). Only complete cases for all covariates were included in the model. All statistical analyses were carried out with R statistical software version 4.0.4 [31].

### 2.6. Availability of Data and Materials

The informed consent given by KORA study participants does not cover data posting in public databases. However, data are available on request from KORA-gen (http://www.helmholtz-muenchen.de/kora-gen, accessed on 1 January 2022). Data requests can be submitted online and are subject to approval by the KORA Board.

## 3. Results

Overall, 595 male and 666 female study participants were included. The participants had a median age of 58 years, a median BMI of 26.8 kg/m^2^ and had a total daily energy intake of ~1800 kcal (Table 1 and Table 2). Metabotypes differed by sex and age. As indicated in Table 1 and Table 2, participants in metabotype subgroup 3 were older and more likely to be male, while a higher proportion of women and younger participants were assigned to metabotype 1. Table 3 shows the median usual dietary intake of food groups and nutrients stratified by sex and metabotype. We analyzed several food groups for an association with DNA methylation in the basic analysis. With a Bonferroni-adjusted *p*-value (and an alpha threshold of 0.05), we found 1 statistically significant signal for the dietary intake of leafy green vegetables, one signal for root vegetables, three signals for cruciferous vegetables, one signal for onions and garlic, one signal for wine and nine signals for beer (Table 4). Genes annotated to these CpGs were SLC7A11, PHGDH, CCDC149 and KIFC1, among others. Methylation of cg06690548 (SLC7A11) was associated with wine. The product of this gene is a sodium-independent amino acid transport system that is highly specific for cysteine and glutamate. Two CpGs, which were significantly associated with beer consumption are located in the PHGDH gene, which is translated to phosphoglycerate dehydrogenase and is involved in L-serine synthesis, an amino acid part of the C1-metabolism. We found no significant associations for other exposures. Volcano plots and a table with the results of all analyzed exposures (including hs-CRP, alcohol in g/day and folic acid, which we did for quality checking of our results) can be found in Table S1 and Figures S1–S38. Table S1 contains all significant signals and 10 CpGs with the lowest *p*-values for food groups where no significant signals were observed at all. For a legend of all supplementary tables, see Tables S1–S3 legend.

**Figure 1:**
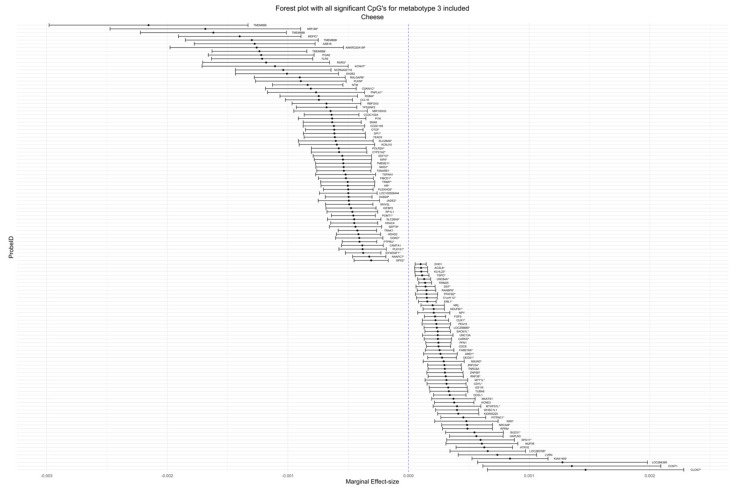
Forest plot for cheese consumption. Y-Axis includes all CpG sites for which there was a significant interaction between cheese and metabotype and had genes annotated to it. Only interactions for metabotype 3 are included. X-axis are marginal effect sizes based on emtrends() function in the emmeans package. Error bars indicate 95% confidence intervals. * Indicate splice variants.

**Figure 2:**
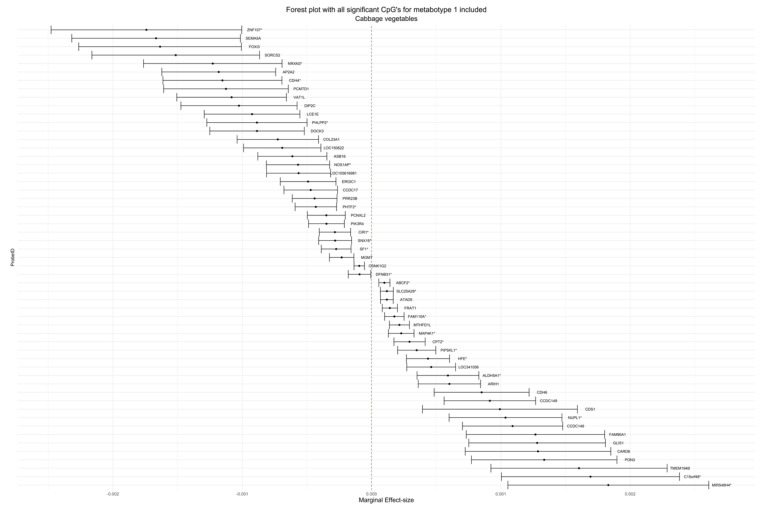
Forest plot for cruciferous vegetables consumption. Y-Axis includes all CpG sites for which there was a significant interaction between cruciferous vegetables and metabotype and had genes annotated to it. Only interactions for metabotype 1 are included. X-axis shows marginal effect sizes based on emtrends() function in the emmeans package. Error bars indicate 95% confidence intervals. * Indicates splice variants.

**Figure 3:**
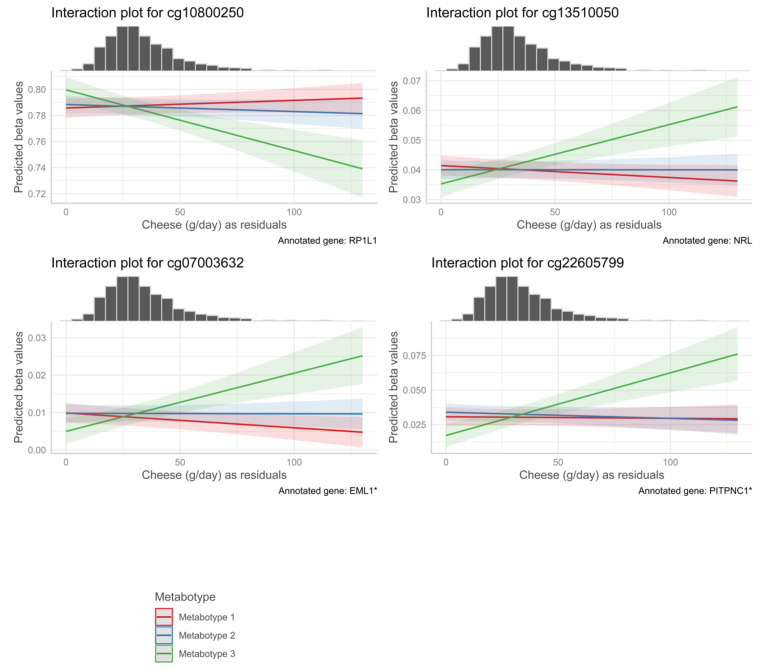
Interaction plot of cheese intake residuals, metabotype and DNA methylation as predicted methylation beta values with marginal histogram. Y-Axis indicates the predicted methylation level based on calculated marginal effect size based on emtrends() function in the emmeans package, by metabotype. X-Axis indicates cheese consumption as residuals and is the same for the histogram and the interaction plot. Interpretability of residuals is possible as, how many grams of cheese is eaten more than average with a given calorie consumption. Marginal histograms show the distribution of the variable plotted on the X-Axis. Shaded areas indicate 95% confidence intervals. * Indicates splice variants.

In the analysis for effect modification of food groups by metabotypes, we evaluated the *p*-value of the metabotype interaction terms. Table 5 contains *p*-values for the calculated marginal effect size, the marginal standard error and the interaction *p*-value adjusted for genomic inflation and corrected by the FDR—for the 10 lowest *p*-values per food group, if available. A table with all statistically significant results of the interaction analysis is provided in Table S2, including data of the analysis of hs-CRP and the nutrients alcohol and folic acid. FDR-corrected *p*-values below an alpha of 0.1 were regarded as statistically significant. We observed much evidence for an effect modification with metabotype for some food groups. These food groups were cruciferous vegetables with 83 signals for mainly metabotype 1 (Figure 1), cheese with 164 signals for metabotype 3 (Figure 2), whole grain products with 17 signals for metabotype 3, total meat with seven signals for metabotype 2, eggs with nine signals for metabotypes 2 and 3 and margarine with 81 signals for metabotype 3. Cruciferous and cheese forest plots were produced to show the wide distribution of effect sizes. See Figures S39–S42 for the remaining forest plots. We checked for genes that appeared multiple times across food groups or in the analysis of one food group. These were ASB16, CCDC149, TMEM88B, KRTAP9-6, and MTHFD1L, which can be found in Table S2 with color codes and as interaction plots in Figures S43–S51 section. The most interesting finding of genes that appeared multiple times is MTHFD1L, which translates to the protein methylenetetrahydrofolate dehydrogenase-1 similar to and essentially part of the regeneration of methionine from homocysteine. We found significant signals for CpGs that were annotated to genes that are associated with eye health: RP1L1, EML1, PITPNC1, NRL (see Figure 3 for interaction plots with calculated marginal effect sizes). Other gene annotations were retinoid X receptor gamma (RXRG), which is a nuclear receptor reacting to retinoic acid, glutathione peroxidase 2 (GPX2), which is a crucial part of the human being’s antioxidant-system and paraoxonase 3 (PON3), which inhibits the oxidation of low-density lipoprotein. The results from the sensitivity analysis, where we accounted for genomic inflation, showed that several associations were no longer statistically significant, although many persisted (see Table S3 for all results and Figures S52–S57 for t- and *p*-value distribution). For the six food groups that we examined for stability of results, 22 associations remained significant for cruciferous vegetables, 33 associations for cheese, 16 associations for whole grain products, zero associations for total meat and eggs, and all associations remained for margarine. None of the CpG-annotated genes associated with eye health persisted. Some examples of CpG sites that were still significant are those annotated to MTHFD1L, HFE, CDH4, TLR5 and 3 of 4 CpGs that were annotated to TMEM88B. In the sensitivity analysis accounting for physical activity and menopause, the *p*-value for all signals remained <0.05, except for one in the food group cruciferous for metabotype 3, see Table S4.

## 4. Discussion

This is the first comprehensive diet EWAS investigating usual food group consumption and effect modification by the participants’ metabolic status as reflected by metabotype clusters. Independent of metabotype, we discovered only very few associations for leafy green vegetables, root vegetables, cruciferous vegetables, onions and garlic, and wine and beer. The main findings of this study are, however, the many interaction effects between food groups and CpG methylation for different metabotype clusters. This highlights the importance of the metabolic characteristics of participants in studies of diet and EWAS.

In our basic model (without metabotype), the most signals were found for beer. Alcohol could be one driving factor for these associations. High consumption of beer in Bavaria, Germany leads to high statistical power, which could explain why there are very few associations in wine and spirits. Additionally, the complex composition of beer, with metabolites generated by yeast and bioactive compounds of hops could be a driving factor for the many associations of DNA methylation and beer [25]. We also tested for folic acid and showed once again that an association of folic acid and DNA methylation in EWAS is not clear [4]. In comparison to Karabegovic et al. [7] we found no association of DNA methylation and either coffee or tea. It is worth noting that they found 11 significant signals with a sample size tenfold of ours, therefore it is clear that the power of our study could be too small to observe these signals as well. The gene SCRIN1, annotated for a CpG signals associated with cruciferous consumption with a positive direction of the effect estimate, encodes a protein which can lead to impaired cell spreading and migration due to inhibiting SRC activity [33]. We use the CpG features and prior knowledge to interpret this finding in this work. Since cruciferous vegetables are often associated with anti-cancer properties [34], it can be assumed that methylation of this gene would lead to enhanced expression. In contrast, the region lies within a CGI, which is often interpreted as an indication of gene suppression if located near a transcription start site (TSS) [35]. However, this locus lies within the gene body, and DNA methylation at loci in the gene body are sometimes interpreted as a gene expression-enhancing factor [36]. The significant signal associated with the food group onions and garlic is cg10399824, which is annotated with the GRK5 gene. This gene is associated with different conditions, such as cartilage degradation [37], cardiac hypertrophy [38], and renal cell carcinoma [39]. The literature supports the idea that components of this food group can be connected to all of these conditions [40,41,42].

In our interaction model, statistically significant interactions with metabotype are apparent for many food group-CpG associations. Most of the significant findings refer to the food groups cruciferous, cheese, whole grain products, margarine, eggs, and total meat. The effect of cruciferous vegetables on changes in DNA methylation can be partially explained by the nutrient and phytochemical profile. Almost all significant signals we found for cruciferous intake were significant in metabotype cluster 1. Cg05305046 lies near the TSS of the CARD6 gene and its methylation level is positively associated with cruciferous consumption, most likely leading to repression of gene expression. Notably, the CARD6 gene can lead to activation of the transcription factor NF-kappa B [43], which often leads to activation of genes involved in inflammation. Among the phytochemicals that could affect DNA methylation, the isothiocyanate sulforaphane was shown to down-regulate DNA methyltransferase (DNMT) activity, resulting in promoter demethylation and enhanced expression (to normal concentration) of antioxidative metabolites such as glutathione S-transferase pi 1 or erythroid 2-related factor 2 [34,44].

Interaction analysis of the food group cheese obtained significant signals associated with the following genes RP1L1, NRL, EML1, and PITPNC1. These are involved in the function of photoreceptors in the eye or diseases affecting the eye, such as diabetic retinopathy (DR) [33]. Yan et al. showed an inverse association of cheese consumption with DR [45]. Additionally, high cheese consumption is associated with lower serum UA concentration [46] and studies have shown a direct association between UA concentration and diabetic retinopathy [47,48]. The NRL gene was also observed to be associated with proliferative DR [49]. Interestingly, the study of Yan et al. has shown that the association of cheese intake and DR is enhanced in a subgroup analysis in participants with a BMI > 25, which supports our result of the interaction effects in participants attributed to metabotype cluster 3. Cheese contains a variety of nutrients and bioactive substances, including fat-soluble vitamins, minerals (especially calcium), and mostly casein as the form of protein [50]. However, few significant signals were identified for consumption of dairy products (with a similar nutrient profile). It is possible that the antioxidative potential of cheese [51], in combination with the high casein content, is a major component driving the significant CpG associations.

Similar to the interpretation of the associations with cheese consumption, the statistically significant findings for margarine, meat, eggs, or whole grain product consumption with DNA methylation may be driven by their effects on metabolic derangements. The consumption of meat could exert effects on DNA methylation via effects on elevated plasma metabolites, for example, and red meat may modulate plasma LDL-cholesterol and HDL cholesterol concentrations [52].

**Figure 4:**
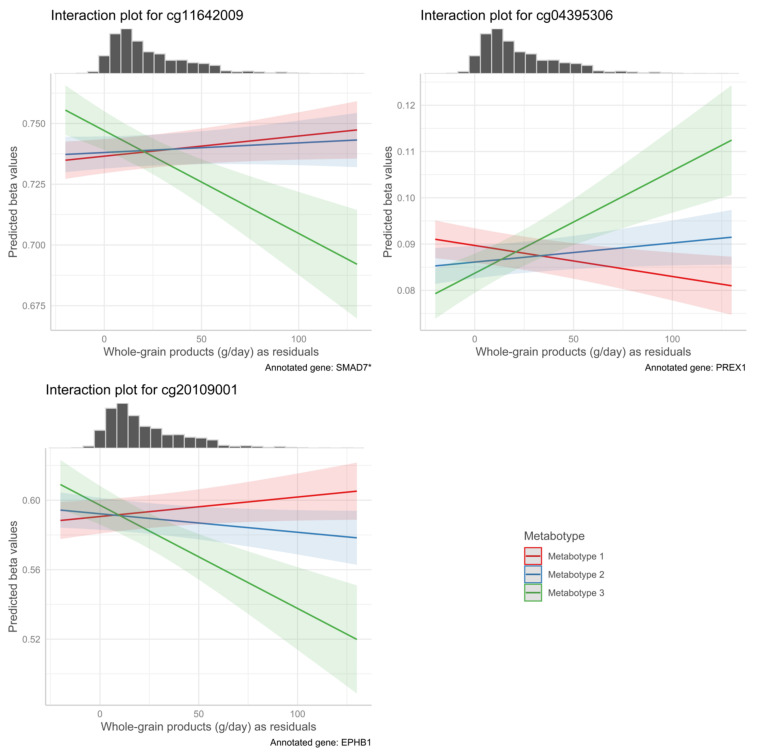
Interaction plot of whole grain product intake residuals, metabotype and DNA methylation as predicted methylation beta values with marginal histogram. Y-axis indicates the predicted methylation level based on calculated marginal effect size based on emtrends() function in the emmeans package, given metabotype. X-Axis indicates whole grain product consumption as residuals and is the same for the histogram and the interaction plot. Interpretability is possible, as written in Figure 3. Marginal Histograms show the distribution of the variable plotted on the X-Axis. Shaded areas indicate 95% confidence intervals. * indicates splice variants.

In addition, associations between DNA methylation status and HDL cholesterol have been reported previously [53]. Whole grain products were associated with a CpG, annotated to SMAD7 (see Figure 4), which has a mechanistic role in the protection of the kidney in participants with diabetes [54]. Additionally, reverse causality is possible, for example, as participants with known elevated blood non-HDL cholesterol concentrations may follow a diet rich in polyunsaturated fatty acids and low in saturated fatty acids in response. Margarine could be either part of or a proxy of such a diet. Margarine intake in our study population is low and it seems surprising that such minor intake could have effects on DNA methylation.

We did not find any significant signals for the dietary scores AHEI and MDS. We assumed substantial effects on DNA methylation based on the epidemiological evidence regarding these scores and chronic diseases [19], especially in participants at high risk [55]. One explanation could be that their favorable effects are not mediated by modification of DNA methylation. Since the metabotype clustering is based on five metabolic parameters, i.e., fasting serum HDL cholesterol, non-HDL cholesterol, plasma glucose, UA and BMI, the question of whether there are some driving variables is valid. We did another interaction analysis using a metabotype clustering approach, replacing UA by triglycerides. The obtained results (not shown) support the idea that UA is a driver of some of the effect modification by metabotype on DNA methylation as observed here. However, the clusters represent the complex metabolic characteristic of the individuals.

Our study has several strengths. First, we calculated habitual dietary data using a blended approach, i.e., combining repeated 24-h recalls and FFQ data, leading to more valid and precise intake estimates as compared to FFQ data alone [56]. Second, we investigated interaction terms instead of stratification of our population, resulting in increased statistical power due to the inclusion of the data of all selected confounders for all participants. Third, to avoid effects being present due to genomic inflation, we used the bacon package in the sensitivity analysis to estimate the empirical null distribution and reduce bias and inflation [28]. Fourth, we saw very consistent results in our interaction analysis, with almost exclusively one metabotype per food group.

Our study also presents some limitations. Our dietary data did not include information about the origin of the food; therefore, it is possible that there is noise in the data regarding organic or conventional origins of our vegetable and meat food groups. Pesticides or different nutrient composition could affect the association of the food groups with DNA methylation [57]. We cannot rule out that some remaining bias arose from diet affecting leukocyte composition and therefore led to changed DNA methylation patterns due to different cell type composition, though we adjusted for cell counts. Since gene expression data are lacking, observed changes in DNA methylation cannot clearly be translated to metabolic changes. We also only had access to whole blood cells, so we cannot draw any tissue-specific conclusions. On a statistical note, despite the debate about the focus on *p*-values [58], we followed this concept because of the explorative nature of this study to have an absolute threshold to decide if we should follow-up on a signal or not. Due to the cross-sectional nature of our study, we cannot conclude on causality and residual confounding cannot be excluded.

In conclusion, the effect modification by metabotype is apparent for various food groups, which underlines the importance of including information on the metabolic state of participants in diet EWAS, though different metabotype definitions may achieve different results. We tested for several food groups and there were few significant signals obtained in the analysis without metabotype. Based on the findings of the interaction analysis, many gene annotations regarding eye health, inflammation and antioxidative system were observed and should be followed up in further studies—especially longitudinal, replication and experimental studies addressing functional consequences of methylation status.

## Figures and Tables

**Table 1:** Population characteristics of male participants stratified by metabotype.

	Overall	Male			
	Overall	Overall	Metabotype 1	Metabotype 2	Metabotype 3
n	1261	595	122	393	80
Age in years (median [IQR])	58.0 [49.0, 66.0]	59.0 [49.0, 68.0]	55.0 [49.0, 65.0]	58.0 [48.0, 67.0]	66.0 [61.0, 73.0]
BMI (WHO-Class.) (%)					
Underweight (x < 18.5)	5 (0.4)	0 (0)	0 (0)	0 (0)	0 (0)
Normal weight (18.5 ≥ x < 25)	407 (32.3)	142 (23.9)	75 (61.5)	59 (15.0)	8 (10.0)
Pre-obesity (25 ≥ x < 30)	520 (41.2)	288 (48.4)	43 (35.2)	224 (57.0)	21 (26.2)
Obesity class I (30 ≥ x < 35)	230 (18.2)	124 (20.8)	3 (2.5)	96 (24.4)	25 (31.2)
Obesity class II (35 ≥ x < 40)	67 (5.3)	29 (4.9)	0 (0.0)	12 (3.1)	17 (21.2)
Obesity class III (x > 40)	32 (2.5)	12 (2.0)	1 (0.8)	2 (0.5)	9 (11.2)
Total energy intake in Kcal/d (median [IQR])	1825.5 [1551.1, 2117.3]	2094.2 [1889.1, 2337.1]	2159.7 [1931.1, 2407.7]	2080.8 [1859.8, 2308.1]	2100.5 [1954.8, 2391.6]
Alcohol g/day (median [IQR])	5.0 [2.4, 13.9]	13.1 [5.1, 24.6]	15.6 [7.4, 26.7]	12.4 [4.6, 23.5]	13.3 [3.9, 23.4]
Smoking behavior (%)					
Regular smoker	178 (14.1)	89 (15.0)	20 (16.4)	62 (15.8)	7 (8.8)
Former smoker	486 (38.5)	273 (45.9)	42 (34.4)	172 (43.8)	59 (73.8)
Never smoker	597 (47.3)	233 (39.2)	60 (49.2)	159 (40.5)	14 (17.5)
Physical activity = Active (%)	777 (61.6)	350 (58.8)	87 (71.3)	231 (58.8)	32 (40.0)
Education in years = < 13 years (%)	790 (62.6)	347 (58.3)	61 (50.0)	235 (59.8)	51 (63.7)

Values are presented as median [Interquartilerange].

**Table 2:** Population characteristics of female participants stratified by metabotype.

	Overall	Female			
	Overall	Overall	Metabotype 1	Metabotype 2	Metabotype 3
n		666	459	167	40
Age in years (median [IQR])		58.0 [48.2, 66.0]	56.0 [47.0, 63.0]	63.0 [55.0, 72.0]	64.0 [60.0, 71.2]
BMI (WHO-Class.) (%)					
Underweight (x < 18.5)		5 (0.8)	5 (1.1)	0 (0.0)	0 (0.0)
Normal weight (18.5 ≥ x < 25)		265 (39.8)	244 (53.2)	20 (12.0)	1 (2.5)
Pre-obesity (25 ≥ x < 30)		232 (34.8)	161 (35.1)	68 (40.7)	3 (7.5)
Obesity class I (30 ≥ x < 35)		106 (15.9)	38 (8.3)	53 (31.7)	15 (37.5)
Obesity class II (35 ≥ x < 40)		38 (5.7)	10 (2.2)	21 (12.6)	7 (17.5)
Obesity class III (x > 40)		20 (3.0)	1 (0.2)	5 (3.0)	14 (35.0)
Total energy intake in Kcal/d (median [IQR])		1578.2 [1428.6, 1793.6]	1607.7 [1441.0, 1816.8]	1534.2 [1419.8, 1716.8]	1526.9 [1397.4, 1752.2]
Alcohol g/day (median [IQR])		2.7 [1.7, 5.3]	3.4 [2.0, 6.1]	1.8 [1.3, 3.5]	1.3 [1.0, 2.3]
Smoking behavior (%)					
Regular smoker		89 (13.4)	64 (13.9)	24 (14.4)	1 (2.5)
Former smoker		213 (32.0)	146 (31.8)	50 (29.9)	17 (42.5)
Never smoker		364 (54.7)	249 (54.2)	93 (55.7)	22 (55.0)
Physical activity = Active (%)		427 (64.1)	320 (69.7)	92 (55.1)	15 (37.5)
Education in years =< 13 years (%)		443 (66.5)	283 (61.7)	127 (76.0)	33 (82.5)

Values are presented as median [Interquartilerange].

**Table 3:** Habitual daily food consumption stratified for sex and metabotype.

	Overall	Male			
	Overall	Overall	Metabotype 1	Metabotype 2	Metabotype 3
n	1261	595	122	393	80
Median [Interquartilerange]					
Protein	67.8 [58.8, 78.6]	76.9 [69.2, 86.5]	77.7 [70.2, 86.8]	75.9 [68.2, 86.0]	80.2 [72.4, 90.2]
Carbohydrates	193.0 [162.0, 228.7]	218.5 [188.6, 250.8]	227.5 [193.9, 263.3]	216.5 [187.2, 251.0]	206.6 [179.6, 238.7]
Fats	75.9 [65.4, 88.6]	87.2 [77.9, 97.7]	87.5 [77.9, 97.6]	86.2 [77.6, 96.3]	92.7 [82.3, 101.5]
Potatoes (g/day)	54.7 [44.4, 68.5]	59.5 [49.1, 73.8]	58.0 [48.3, 68.7]	59.5 [49.2, 74.1]	63.2 [52.6, 82.4]
Total Vegetables (g/day)	163.3 [132.7, 204.0]	147.4 [121.2, 184.3]	159.4 [127.3, 195.1]	145.6 [121.6, 181.6]	142.5 [112.9, 168.4]
Leafy Vegetables (g/day)	23.1 [15.3, 31.7]	24.0 [15.7, 31.7]	25.0 [15.6, 31.7]	23.9 [16.0, 32.6]	20.6 [14.7, 30.1]
Fruit vegetables (g/day)	71.6 [54.5, 96.4]	62.6 [49.0, 84.2]	69.7 [56.0, 96.2]	60.8 [48.6, 81.1]	54.9 [41.5, 78.9]
Root vegetables (g/day)	15.4 [10.7, 25.2]	12.4 [9.5, 19.4]	14.7 [10.0, 19.7]	12.7 [9.7, 19.5]	9.6 [8.1, 14.1]
Cruciferous vegetables (g/day)	14.5 [11.3, 19.2]	14.1 [11.3, 18.7]	13.4 [11.5, 17.3]	13.9 [10.7, 18.2]	16.6 [13.5, 21.9]
Mushrooms (g/day)	2.3 [1.6, 3.7]	2.2 [1.4, 3.4]	2.4 [2.0, 4.2]	2.1 [1.5, 3.4]	1.8 [1.1, 2.5]
Onions & garlic (g/day)	6.4 [4.4, 9.0]	6.1 [4.0, 8.7]	4.9 [3.3, 7.3]	6.1 [4.1, 8.9]	7.3 [5.8, 9.9]
Legumes (g/day)	4.8 [3.6, 6.8]	4.1 [3.3, 6.1]	4.2 [3.5, 6.0]	4.0 [3.2, 6.1]	4.4 [3.5, 6.2]
Total fruit (g/day)	141.2 [87.9, 201.6]	133.9 [75.2, 196.3]	136.9 [76.6, 190.3]	132.4 [71.9, 199.1]	135.6 [87.8, 215.1]
Nuts & seeds (g/day)	4.2 [3.0, 8.9]	4.5 [3.3, 8.9]	5.1 [3.4, 9.0]	4.3 [3.2, 8.8]	4.8 [3.3, 11.0]
Milk (g/day)	73.8 [27.6, 140.5]	59.0 [19.5, 122.2]	61.0 [20.5, 125.4]	62.2 [19.8, 130.8]	45.5 [17.3, 83.7]
Yogurt (g/day)	30.7 [14.0, 66.8]	21.1 [11.9, 52.8]	31.6 [12.5, 64.7]	20.8 [11.9, 49.2]	15.6 [11.8, 43.1]
Cheese (g/day)	30.2 [21.4, 41.9]	30.4 [21.4, 42.5]	33.0 [22.1, 47.4]	29.6 [21.0, 40.5]	31.4 [22.1, 44.9]
Cream (g/day)	1.4 [1.2, 2.2]	1.4 [1.2, 1.8]	1.4 [1.1, 1.6]	1.4 [1.2, 1.9]	1.4 [1.2, 1.7]
Grain products (g/day)	161.6 [133.5, 195.4]	187.8 [162.2, 218.6]	203.5 [168.5, 239.7]	184.7 [159.5, 215.0]	178.3 [163.8, 213.6]
Whole grain products (g/day)	16.5 [7.3, 34.5]	14.2 [6.9, 36.5]	17.6 [7.6, 38.7]	14.2 [6.8, 36.5]	10.6 [5.4, 27.6]
Total meat (g/day)	107.2 [83.4, 142.6]	142.0 [119.2, 166.3]	131.2 [105.9, 152.1]	143.1 [120.6, 165.1]	158.7 [132.0, 198.4]
Fresh red meat (g/day)	42.6 [33.2, 54.6]	54.0 [46.5, 64.6]	51.8 [45.0, 59.0]	55.1 [47.6, 65.9]	54.9 [44.3, 67.0]
Processed meat (g/day)	42.5 [29.3, 62.7]	61.0 [47.0, 79.2]	53.9 [37.2, 71.0]	60.3 [47.8, 77.7]	74.5 [57.2, 102.1]
Total fish (g/day)	16.3 [11.8, 24.6]	18.2 [13.1, 28.0]	19.8 [13.6, 29.3]	18.0 [13.0, 27.0]	19.0 [13.0, 28.3]
Eggs (g/day)	13.4 [10.2, 19.2]	14.1 [10.5, 20.9]	13.0 [10.5, 18.7]	13.8 [10.3, 20.9]	16.9 [12.3, 24.3]
Plant oils (g/day)	5.3 [3.6, 8.0]	5.6 [3.6, 8.5]	5.4 [3.6, 8.2]	5.5 [3.5, 8.3]	6.2 [4.1, 9.6]
Butter (g/day)	13.7 [7.8, 17.4]	16.5 [9.1, 21.8]	18.7 [10.6, 23.1]	16.0 [8.9, 21.4]	16.2 [8.4, 21.3]
Margarine (g/day)	0.6 [0.3, 1.8]	0.8 [0.5, 2.8]	0.7 [0.4, 2.0]	0.8 [0.5, 2.7]	0.9 [0.7, 3.9]
Total sweets (g/day)	35.1 [25.9, 46.0]	37.7 [27.0, 49.9]	44.2 [31.8, 54.2]	37.5 [27.2, 49.9]	29.4 [22.2, 40.3]
Cakes (g/day)	48.8 [38.6, 63.8]	53.9 [40.2, 70.3]	58.5 [41.0, 73.0]	53.5 [39.9, 68.5]	52.4 [40.5, 71.0]
Sugar sweetened beverages (g/day)	6.7 [3.6, 24.6]	10.8 [6.1, 67.2]	8.0 [4.7, 19.0]	11.4 [6.7, 64.4]	14.7 [6.8, 104.2]
Coffee (g/day)	435.0 [365.1, 478.3]	445.1 [375.0, 497.7]	443.4 [389.9, 503.7]	445.1 [371.9, 494.0]	450.6 [369.5, 501.2]
Tea (g/day)	63.4 [27.6, 322.5]	35.7 [22.0, 223.3]	64.1 [25.2, 364.3]	34.6 [22.0, 201.1]	30.4 [18.9, 199.8]
Wine (g/day)	17.6 [11.9, 39.4]	18.4 [12.7, 44.6]	24.4 [17.5, 63.9]	17.5 [12.7, 38.3]	11.8 [8.2, 37.3]
Beer (g/day)	39.7 [6.5, 204.4]	208.2 [50.8, 482.6]	223.6 [55.4, 560.6]	204.4 [51.5, 472.2]	210.4 [43.3, 474.1]
Spirits (g/day)	0.3 [0.2, 0.5]	0.4 [0.3, 0.7]	0.5 [0.3, 0.8]	0.4 [0.3, 0.7]	0.3 [0.2, 0.4]
Alcohol (g/day)	5.0 [2.4, 13.9]	13.1 [5.1, 24.6]	15.6 [7.4, 26.7]	12.4 [4.6, 23.5]	13.3 [3.9, 23.4]
AHEI	42.5 [36.2, 48.9]	41.1 [34.7, 46.8]	42.8 [37.1, 49.6]	40.8 [34.8, 45.9]	40.4 [33.6, 46.6]
MDS	4.0 [3.0, 6.0]	5.0 [3.0, 6.0]	5.0 [4.0, 6.0]	4.0 [3.0, 6.0]	5.0 [3.8, 6.0]
Folic acid (µg/d)	200.1 [169.7, 237.7]	212.6 [179.2, 249.5]	223.6 [185.6, 257.2]	208.6 [176.7, 245.6]	215.1 [180.7, 257.5]
	**Overall**	**Female**			
	**Overall**	**Overall**	**Metabotype 1**	**Metabotype 2**	**Metabotype 3**
n		666	459	167	40
Median [Interquartilerange]					
Protein		76.9 [69.2, 86.5]	77.7 [70.2, 86.8]	75.9 [68.2, 86.0]	80.2 [72.4, 90.2]
Carbohydrates		218.5 [188.6, 250.8]	227.5 [193.9, 263.3]	216.5 [187.2, 251.0]	206.6 [179.6, 238.7]
Fats		87.2 [77.9, 97.7]	87.5 [77.9, 97.6]	86.2 [77.6, 96.3]	92.7 [82.3, 101.5]
Potatoes (g/day)		50.4 [40.6, 63.9]	49.0 [39.5, 61.0]	55.8 [42.1, 71.6]	52.1 [43.0, 66.9]
Total Vegetables (g/day)		178.1 [146.2, 218.7]	182.1 [150.4, 224.4]	176.3 [139.1, 215.8]	163.4 [146.1, 191.0]
Leafy Vegetables (g/day)		22.8 [14.9, 32.0]	22.9 [14.8, 32.4]	22.8 [15.3, 31.5]	21.9 [14.8, 26.7]
Fruit vegetables (g/day)		81.2 [61.6, 106.7]	82.8 [63.2, 110.9]	77.1 [58.1, 104.0]	75.9 [54.5, 90.5]
Root vegetables (g/day)		18.9 [13.1, 30.6]	20.9 [14.1, 33.3]	15.1 [11.4, 26.1]	12.2 [10.4, 16.7]
Cruciferous vegetables (g/day)		14.8 [11.4, 20.1]	14.1 [10.9, 18.8]	16.4 [12.4, 21.8]	15.9 [12.3, 22.6]
Mushrooms (g/day)		2.4 [1.7, 3.9]	2.6 [2.0, 4.5]	2.1 [1.2, 2.7]	1.9 [1.4, 2.4]
Onions & garlic (g/day)		6.7 [4.7, 9.3]	6.3 [4.5, 8.4]	7.4 [5.2, 9.9]	9.5 [7.2, 12.9]
Legumes (g/day)		5.2 [4.2, 7.5]	5.3 [4.2, 8.0]	5.3 [4.2, 7.1]	5.0 [3.7, 6.4]
Total fruit (g/day)		145.4 [96.5, 203.3]	143.0 [93.3, 201.6]	154.4 [100.2, 212.3]	144.3 [95.8, 192.5]
Nuts & seeds (g/day)		4.0 [2.6, 8.7]	4.3 [2.8, 9.4]	3.3 [2.4, 6.0]	3.5 [2.3, 7.4]
Milk (g/day)		86.8 [42.8, 150.6]	92.6 [45.0, 160.3]	80.5 [37.3, 129.8]	83.4 [26.8, 122.2]
Yogurt (g/day)		38.6 [17.9, 76.1]	40.4 [18.7, 79.9]	36.4 [15.9, 70.8]	23.4 [17.0, 49.9]
Cheese (g/day)		29.8 [21.5, 41.8]	30.5 [21.8, 42.0]	27.5 [20.1, 39.8]	26.1 [20.1, 38.8]
Cream (g/day)		1.5 [1.2, 2.5]	1.5 [1.2, 2.6]	1.5 [1.2, 2.5]	1.4 [1.2, 2.1]
Grain products (g/day)		138.1 [121.1, 163.9]	143.0 [123.6, 169.3]	129.6 [117.0, 153.3]	129.8 [109.8, 141.2]
Whole grain products (g/day)		18.0 [8.3, 34.1]	19.2 [8.7, 35.2]	15.7 [7.0, 29.3]	16.2 [9.7, 29.5]
Total meat (g/day)		86.0 [72.9, 101.7]	81.9 [69.9, 96.6]	90.6 [78.5, 108.8]	104.8 [93.2, 134.2]
Fresh red meat (g/day)		34.0 [29.5, 40.1]	33.7 [29.5, 39.4]	35.6 [29.0, 41.0]	33.5 [30.4, 43.0]
Processed meat (g/day)		31.0 [24.0, 41.6]	29.1 [22.8, 37.4]	34.4 [27.4, 46.8]	49.7 [40.2, 69.4]
Total fish (g/day)		14.2 [10.9, 21.8]	13.7 [10.7, 21.7]	15.0 [11.9, 22.1]	12.8 [11.1, 19.0]
Eggs (g/day)		13.0 [9.9, 17.9]	12.9 [9.9, 18.0]	13.2 [10.1, 17.9]	13.0 [9.5, 16.4]
Plant oils (g/day)		5.2 [3.6, 7.6]	5.1 [3.5, 7.6]	5.3 [3.7, 7.7]	4.4 [3.6, 6.3]
Butter (g/day)		12.0 [7.0, 15.3]	12.5 [7.4, 15.4]	11.0 [6.2, 15.0]	9.9 [6.1, 14.5]
Margarine (g/day)		0.4 [0.2, 1.0]	0.3 [0.2, 0.8]	0.5 [0.3, 1.6]	0.8 [0.4, 2.3]
Total sweets (g/day)		33.4 [24.9, 43.1]	34.3 [25.8, 44.8]	31.7 [23.7, 39.8]	30.2 [23.1, 40.4]
Cakes (g/day)		46.2 [37.5, 57.9]	47.4 [38.5, 58.8]	43.9 [36.1, 55.3]	39.4 [34.5, 47.9]
Sugar sweetened beverages (g/day)		4.2 [2.8, 8.4]	3.9 [2.6, 7.1]	4.5 [3.0, 14.2]	6.6 [4.4, 65.6]
Coffee (g/day)		419.5 [356.7, 465.6]	412.0 [351.8, 467.0]	430.0 [366.8, 464.2]	430.4 [365.5, 455.1]
Tea (g/day)		135.7 [38.2, 372.5]	151.4 [41.8, 377.5]	124.5 [34.2, 343.8]	53.5 [27.6, 278.4]
Wine (g/day)		17.0 [11.0, 36.1]	19.4 [14.4, 43.4]	11.6 [8.2, 19.8]	7.4 [5.3, 9.9]
Beer (g/day)		6.7 [5.7, 8.2]	7.2 [6.0, 8.8]	6.0 [5.0, 7.0]	5.2 [4.6, 6.1]
Spirits (g/day)		0.2 [0.1, 0.3]	0.2 [0.2, 0.3]	0.1 [0.1, 0.2]	0.1 [0.1, 0.1]
Alcohol (g/day)		2.7 [1.7, 5.3]	3.4 [2.0, 6.1]	1.8 [1.3, 3.5]	1.3 [1.0, 2.3]
AHEI		43.9 [37.7, 50.5]	45.2 [39.4, 51.7]	42.0 [35.6, 48.0]	36.2 [31.3, 40.8]
MDS		4.0 [3.0, 6.0]	4.0 [3.0, 6.0]	4.0 [3.0, 5.0]	3.0 [3.0, 4.0]
Folic acid (µg/d)		190.3 [162.7, 224.7]	194.3 [166.8, 230.5]	182.0 [155.2, 216.8]	179.3 [155.0, 199.4]

Values are presented as median [Interquartilerange].

**Table 4:** Results for basic model epigenome-wide association study.

ProbeID	Sample Size	Effect Size **	Standard Error	*p*-Value	Foodgroup	Chr	RefGene Name	RefGene Group	Relation to CpG Island
cg01838728	1319	−8.91 × 10^−4^	1.60 × 10^−4^	0.0268	Leafy vegetables	15	N/A	N/A	N/A
cg15351590	1321	−1.82 × 10^−4^	3.16 × 10^−5^	0.00809	Root vegetables	6	KIFC1	TSS1500	N_Shore
cg14698575	1319	8.51 × 10^−4^	1.37 × 10^−4^	6.27 × 10^−4^	Cruciferous vegetables	9	N/A	N/A	S_Shore
cg23709902	1310	4.40 × 10^−4^	7.90 × 10^−5^	0.0243	Cruciferous vegetables	17	SRCIN1	Body	Island
cg06102690	1319	6.72 × 10^−4^	1.24 × 10^−4^	0.0494	Cruciferous vegetables	4	CCDC149	TSS200	N/A
cg10399824	1322	−6.43 × 10^−4^	1.11 × 10^−4^	0.00596	Onions-garlic	10	GRK5	Body	N/A
cg06690548	1277	−1.04 × 10^−4^	1.88 × 10^−5^	0.0269	Wine	4	SLC7A11	Body	N/A
cg06690548	1277	−5.10 × 10^−5^	5.21 × 10^−6^	6.01 × 10^−16^	Beer	4	SLC7A11	Body	N/A
cg26457483	1319	−6.03 × 10^−5^	8.37 × 10^−6^	7.99 × 10^−7^	Beer	1	PHGDH	Body	S_Shore
cg14476101	1320	−6.32 × 10^−5^	9.30 × 10^−6^	1.31 × 10^−5^	Beer	1	PHGDH	Body	S_Shore
cg06088069	1319	−2.71 × 10^−5^	4.32 × 10^−6^	3.74 × 10^−4^	Beer	14	JDP2 *	5′UTR *	S_Shore
cg16246545	1320	−4.68 × 10^−5^	7.85 × 10^−6^	0.00250	Beer	1	PHGDH	Body	S_Shore
cg15837522	1322	−6.45 × 10^−5^	1.09 × 10^−5^	0.00324	Beer	8	N/A	N/A	N/A
cg18120259	1320	−3.23 × 10^−5^	5.59 × 10^−6^	0.00755	Beer	6	LOC100132354	Body	N/A
cg08228578	1322	−2.39 × 10^−5^	4.21 × 10^−6^	0.0125	Beer	12	SHMT2 *	Body *	S_Shore
cg10223198	1322	−2.88 × 10^−5^	5.27 × 10^−6^	0.0427	Beer	11	N/A	N/A	N/A

Shown are all significant signals with bonferroni corrected *p*-values < 0.05; ** Effect sizes are %-methylation change per gram residual intake; UCSC RefGene Name—Target gene names from the UCSC database; UCSC RefGene Group—Describing CpG position. TSS1500 = 200–1500 bases upstream of the Transcription start site (TSS); 5-UTR = Within the 5′ untranslated region, between the TSS and the ATG start site; Body = Between the ATG and stop codon, irrespective of the presence of introns, exons, TSS or promoters; 3′UTR = Between the stop codon and the poly A signal Relation to UCSC CpG Island—The location of the CpG relative to the CpG Island. Shore = 0–2 kb from Island; Shelf = 2–4 kb from Island; N = Upstream (5′) of CpG Island; S = Downstream (3′) of CpG Island [32]; * indicates available splice variants. N/A—Not available.

**Table 5:** Results for interaction of food group with metabotype epigenome-wide association study.

ProbeID	Effect Size **	Standard Error	*p*-Value	*p*-Value (Bacon)	Foodgroup	Cluster	Chr	RefGene Name	RefGene Group	Relation to CpG Island
cg00067414	2.15 × 10^−4^	3.94 × 10^−5^	0.01538	0.03974	Cruciferous	Metabotype 1	6	MTHFD1L	Body	Island
cg20561564	−1.31 × 10^−3^	2.40 × 10^−4^	0.01538	0.03974	Cruciferous	Metabotype 1	9	N/A	N/A	N/A
cg11945292	1.09 × 10^−3^	1.98 × 10^−4^	0.01538	0.03974	Cruciferous	Metabotype 1	4	CCDC149	TSS200	N/A
cg22614518	−4.31 × 10^−4^	8.16 × 10^−5^	0.02687	0.06638	Cruciferous	Metabotype 1	7	PHTF2 *	Body *	N/A
cg04183158	−1.18 × 10^−3^	2.25 × 10^−4^	0.02687	0.06638	Cruciferous	Metabotype 1	11	AP2A2	3′UTR	S_Shore
cg06892726	4.37 × 10^−4^	8.50 × 10^−5^	0.04280	0.09608	Cruciferous	Metabotype 1	6	HFE *	1stExon *	N/A
cg23160569	−3.49 × 10^−4^	7.07 × 10^−5^	0.04454	0.09608	Cruciferous	Metabotype 1	3	PIK3R4	Body	N/A
cg23923117	−8.52 × 10^−4^	1.74 × 10^−4^	0.04454	0.09608	Cruciferous	Metabotype 1	2	N/A	N/A	N/A
cg01841471	−1.21 × 10^−3^	2.43 × 10^−4^	0.04454	0.09608	Cruciferous	Metabotype 1	13	N/A	N/A	S_Shelf
cg08921926	6.02 × 10^−4^	1.23 × 10^−4^	0.04454	0.09608	Cruciferous	Metabotype 1	15	ARIH1	TSS1500	N_Shore
cg00073181	−1.21 × 10^−3^	2.14 × 10^−4^	0.00116	0.00167	Cheese	Metabotype 3	1	TLR5	5′UTR	N/A
cg23795938	−1.24 × 10^−3^	2.00 × 10^−4^	0.00249	0.00350	Cheese	Metabotype 3	1	TMEM88B	TSS200	N_Shore
cg04045906	−6.27 × 10^−4^	1.08 × 10^−4^	0.00555	0.00766	Cheese	Metabotype 3	4	N/A	N/A	N/A
cg10888278	−8.36 × 10^−4^	1.49 × 10^−4^	0.01856	0.02485	Cheese	Metabotype 3	11	NTM	Body	N/A
cg15379294	−5.78 × 10^−4^	1.16 × 10^−4^	0.02049	0.03083	Cheese	Metabotype 3	3	POLR2H *	TSS1500 *	N_Shore
cg00741624	8.41 × 10^−4^	1.61 × 10^−4^	0.02049	0.04099	Cheese	Metabotype 3	14	KIAA1409	5′UTR	Island
cg18244100	−4.92 × 10^−4^	1.00 × 10^−4^	0.02049	0.03083	Cheese	Metabotype 3	6	SKIV2L	Body	N_Shelf
cg21880900	−7.15 × 10^−4^	1.60 × 10^−4^	0.02049	0.02728	Cheese	Metabotype 3	3	N/A	N/A	N/A
cg12274082	−5.76 × 10^−4^	1.17 × 10^−4^	0.02423	0.03569	Cheese	Metabotype 3	6	CYP21A2 *	Body *	N/A
cg05531689	−6.16 × 10^−4^	1.22 × 10^−4^	0.03207	0.04485	Cheese	Metabotype 3	2	OTOF	Body	S_Shelf
cg00039945	−7.68 × 10^−4^	1.24 × 10^−4^	0.05176	0.03773	Whole grain	Metabotype 3	1	LGR6 *	Body *	N/A
cg12515635	−7.85 × 10^−4^	1.79 × 10^−4^	0.05176	0.03773	Whole grain	Metabotype 3	15	KLF13	Body	N_Shelf
cg16687213	−1.78 × 10^−3^	3.74 × 10^−4^	0.05176	0.03773	Whole grain	Metabotype 3	7	TRIM4 *	TSS1500 *	S_Shore
cg07268926	−6.94 × 10^−4^	1.50 × 10^−4^	0.05351	0.05357	Whole grain	Metabotype 3	11	IGSF9B	Body	N/A
cg04395306	2.21 × 10^−4^	5.12 × 10^−5^	0.05351	0.06912	Whole grain	Metabotype 3	20	PREX1	Body	Island
cg10143811	4.40 × 10^−4^	1.06 × 10^−4^	0.05351	0.07192	Whole grain	Metabotype 3	12	LMO3 *	5′UTR *	N/A
cg10762466	7.30 × 10^−4^	1.42 × 10^−4^	0.06360	0.07745	Whole grain	Metabotype 3	19	N/A	N/A	N_Shore
cg01755100	−8.44 × 10^−4^	1.82 × 10^−4^	0.07429	0.06912	Whole grain	Metabotype 3	17	N/A	N/A	S_Shelf
cg15200604	−7.81 × 10^−4^	1.84 × 10^−4^	0.07429	0.06912	Whole grain	Metabotype 3	13	N/A	N/A	N/A
cg00880872	−5.80 × 10^−4^	1.34 × 10^−4^	0.07429	0.06912	Whole grain	Metabotype 3	9	N/A	N/A	N_Shore
cg18029285	2.67 × 10^−4^	6.16 × 10^−5^	0.00771	0.43405	Total meat	Metabotype 2	17	KRTAP9-6	TSS1500	N/A
cg06713760	1.37 × 10^−4^	4.30 × 10^−5^	0.02080	0.81005	Total meat	Metabotype 2	10	N/A	N/A	S_Shelf
cg05581388	2.19 × 10^−4^	5.30 × 10^−5^	0.03327	0.95588	Total meat	Metabotype 2	17	KRTAP9-6	TSS1500	N/A
cg08991742	6.48 × 10^−5^	1.72 × 10^−5^	0.04204	0.95588	Total meat	Metabotype 2	2	ARHGAP25 *	5′UTR *	N/A
cg27582585	6.24 × 10^−5^	2.93 × 10^−5^	0.07862	0.95588	Total meat	Metabotype 2	1	KLHDC9 *	Body *	S_Shore
cg05831315	1.15 × 10^−4^	3.55 × 10^−5^	0.08613	0.95588	Total meat	Metabotype 2	8	N/A	N/A	N/A
cg10919344	1.35 × 10^−4^	4.72 × 10^−5^	0.08613	0.95588	Total meat	Metabotype 2	11	OR5A1	TSS200	N/A
cg07454320	3.95 × 10^−4^	7.43 × 10^−5^	0.08825	0.68647	Eggs	Metabotype 3	1	WNT2B *	TSS200 *	Island
cg17634390	−1.74 × 10^−3^	3.24 × 10^−4^	0.08825	0.68647	Eggs	Metabotype 3	4	COX7B2	5′UTR	N/A
cg13202871	−2.15 × 10^−3^	4.30 × 10^−4^	0.08825	0.68647	Eggs	Metabotype 3	12	SLCO1B7 *	ExonBnd *	N/A
cg23049758	−7.36 × 10^−4^	1.62 × 10^−4^	0.08825	0.68647	Eggs	Metabotype 3	17	SPAG9 *	Body *	N/A
cg09034467	−1.83 × 10^−3^	4.23 × 10^−4^	0.08825	0.68647	Eggs	Metabotype 3	21	N/A	N/A	N/A
cg00857137	−1.06 × 10^−3^	2.46 × 10^−4^	0.09857	0.73530	Eggs	Metabotype 3	19	TLE2 *	Body *	Island
cg16181002	3.62 × 10^−3^	5.97 × 10^−4^	0.01779	0.01344	Margarine	Metabotype 3	6	PARK2 *	Body *	N/A
cg05534678	4.58 × 10^−4^	1.25 × 10^−4^	0.07021	0.06355	Margarine	Metabotype 3	16	ZNF688 *	5′UTR *	Island
cg23229016	1.10 × 10^−3^	2.05 × 10^−4^	0.07021	0.06355	Margarine	Metabotype 3	1	RPS6KA1 *	1stExon *	N/A
cg08027748	−9.80 × 10^−4^	2.01 × 10^−4^	0.07021	0.06355	Margarine	Metabotype 3	3	UROC1 *	TSS1500 *	N/A
cg07199337	2.10 × 10^−3^	4.67 × 10^−4^	0.07021	0.06355	Margarine	Metabotype 3	11	PRMT3 *	TSS1500 *	N_Shore
cg25141008	1.67 × 10^−3^	4.51 × 10^−4^	0.07021	0.06355	Margarine	Metabotype 3	20	C20orf27 *	TSS1500 *	S_Shore
cg08644318	5.61 × 10^−4^	1.60 × 10^−4^	0.07021	0.06355	Margarine	Metabotype 3	3	YEATS2	TSS1500	N_Shore
cg02958895	1.92 × 10^−3^	4.41 × 10^−4^	0.07021	0.06355	Margarine	Metabotype 3	1	N/A	N/A	S_Shore
cg25356086	6.57 × 10^−4^	1.39 × 10^−4^	0.07021	0.06355	Margarine	Metabotype 3	21	C21orf119 *	TSS1500 *	N_Shore
cg26536849	−6.74 × 10^−4^	2.06 × 10^−4^	0.07021	0.07213	Margarine	Metabotype 3	20	DDX27	Body	N/A

** Effect sizes are %-methylation change per gram residual intake in comparison to the reference (metabotype 1) Shown are marginal effect size and standard errors and resulting *p*-values (FDR-corrected) for the 10 lowest significant *p*-values per food group, if available. Only food groups with at least four significant signals were selected. Sample size is not shown due to size limits, but is at minimum n = 1238. UCSC RefGene Group—Describing CpG position. TSS1500 = 200–1500 bases upstream of the Transcription start site (TSS); 5-UTR = Within the 5′ untranslated region, between the TSS and the ATG start site; Body = Between the ATG and stop codon, irrespective of the presence of introns, exons, TSS or promoters; 3′UTR = Between the stop codon and the poly A signal UCSC RefGene Name—Target gene names from the UCSC database. Relation to UCSC CpG Island—The location of the CpG relative to the CpG Island. Shore = 0–2 kb from Island; Shelf = 2–4 kb from Island; N = Upstream (5′) of CpG Island; S = Downstream (3′) of CpG Island [32]; * indicates available splice variants. N/A—Not available.

## Data Availability

The data are subject to national data protection laws and restrictions were imposed by the Ethics Committee of the Bavarian Chamber of Physicians to ensure data privacy of the study participants. Therefore, data cannot be made freely available in a public repository. However, data can be requested through an individual project agreement with KORA via the online portal KORA.passt (https://www.helmholtz-munich.de/epi/index.html, accessed on 14 June 2022).

## Supplementary Materials

The following supporting information can be downloaded at: https://www.mdpi.com/article/10.3390/life12071064/s1, Table S1: Results of the general analysis for all food groups; Table S2: Results of interaction analysis for all food groups; Table S3: Results of genomic inflation sensitivity interaction analyses; Table S4: Results of sensitivity analysis for all food groups 1; Figures S1–S38: Volcano plots of basic EWAS; Figures S39–S42: Forest plots for EWAS with eggs, margarine, total meat and whole grain; Figures S43–S51: Interaction plots; Figures S52–S57: Plots displaying effect of bacon correction on genomic inflation: A QQ-plot of unaltered *p*-values, B Histogram of T-statistics, C Histogram of *p*-values.

## Abbreviations

AHEI	Alternate Healthy Eating Index
CGI	CpG Island
DNMT	DNA methyltransferase
DR	Diabetic retinopathy
EWAS	Epigenome-wide association study
FDR	False Discovery Rate
FFQ	Food frequency questionnaire
MDS	Mediterranean Diet Score
QN	Quantile normalization
TSS	Transcription start site
UA	Uric acid
