# Supplementary material for: Association between Usual Dietary Intake of Food Groups and DNA Methylation and Effect Modification by Metabotype in the KORA FF4 Cohort
Source: Life (Basel). 2022 Jul 15;12(7):1064. doi: 10.3390/life12071064 (PMC9318948; doi:10.3390/life12071064)

Forest plot description 39-42: Y-Axis are all CpG sites for whose there was a significant interaction between respective food group and metabotype and had genes annotated to it. Only interactions for metabotype cluster (x) are included. X-axis are marginal effect sizes based on emtrends() function in the emmeans package.

Figure S39

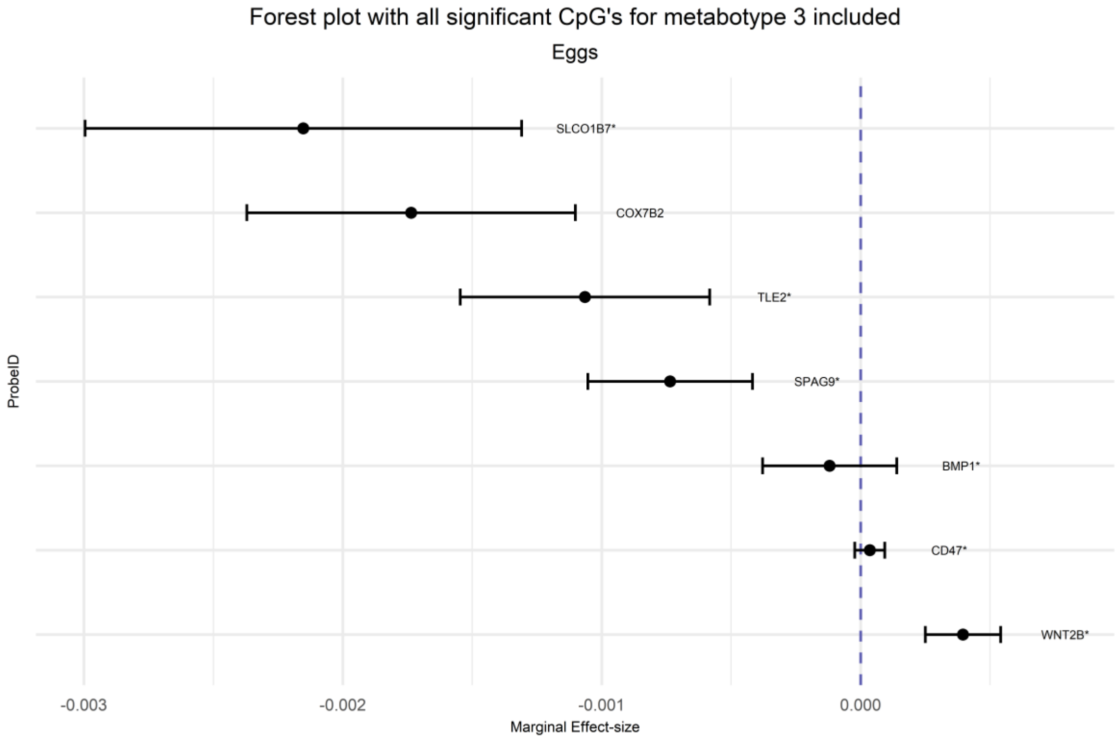

Figure S40

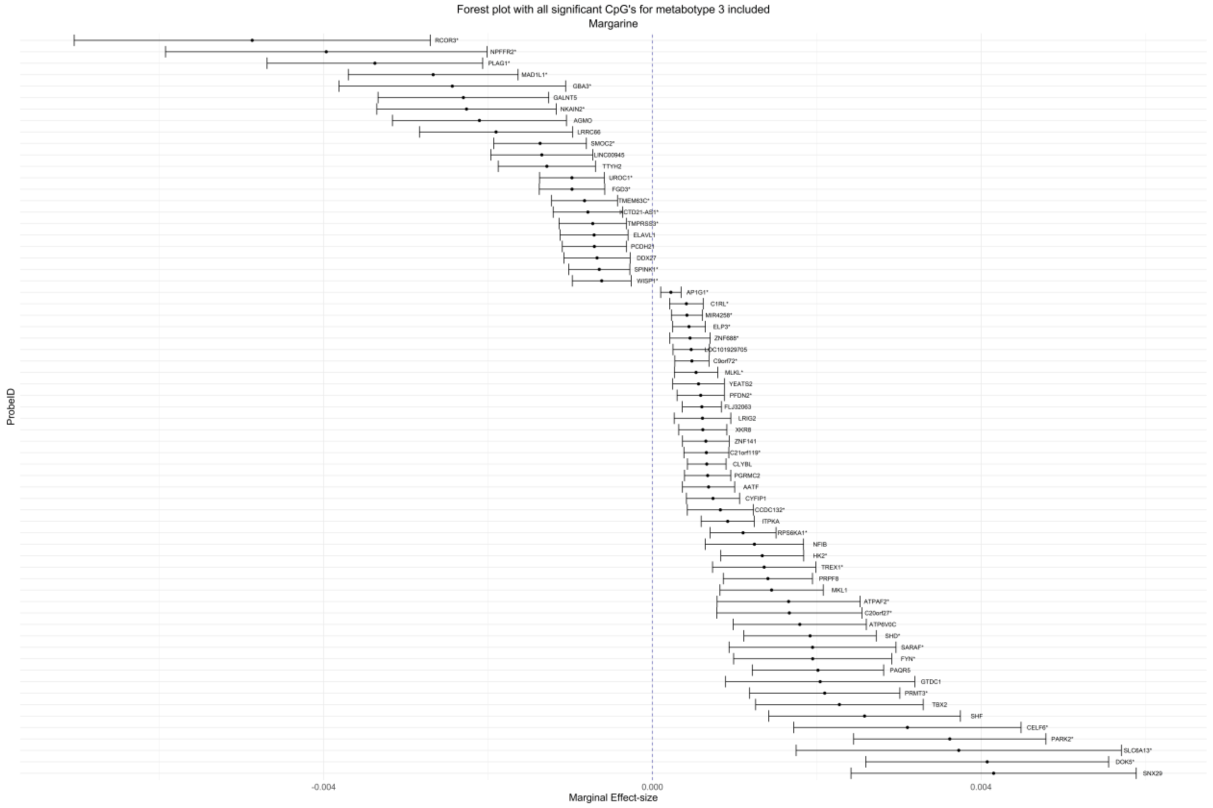

Figure S41

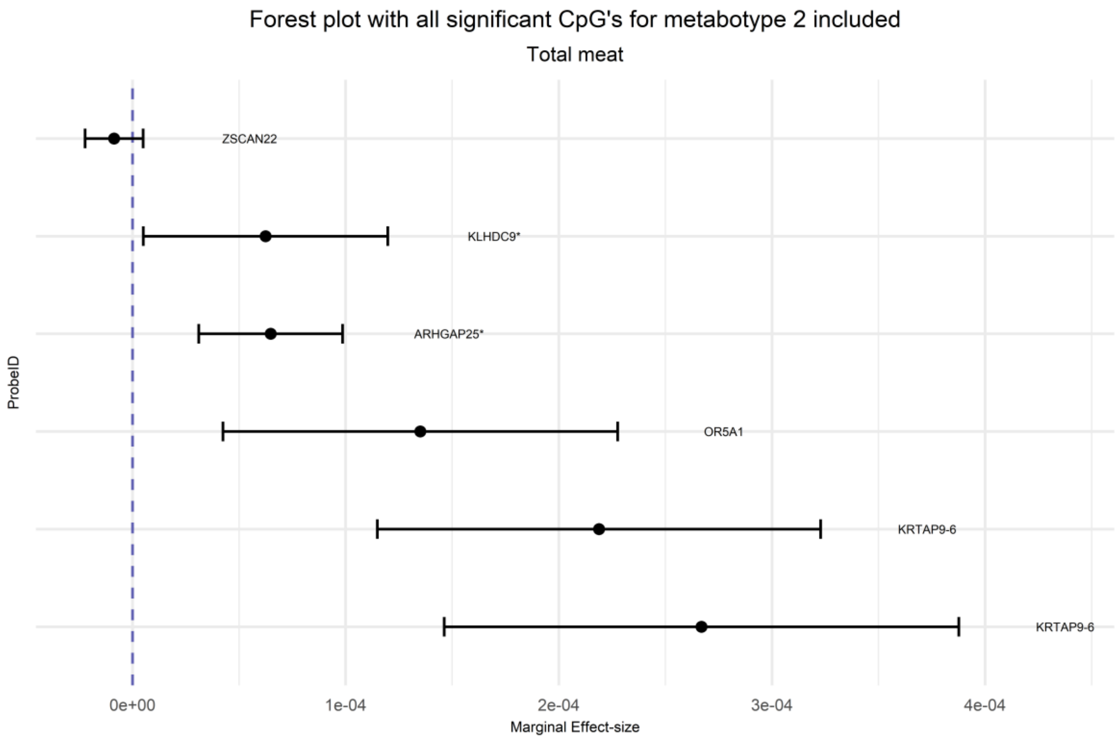

Figure S42

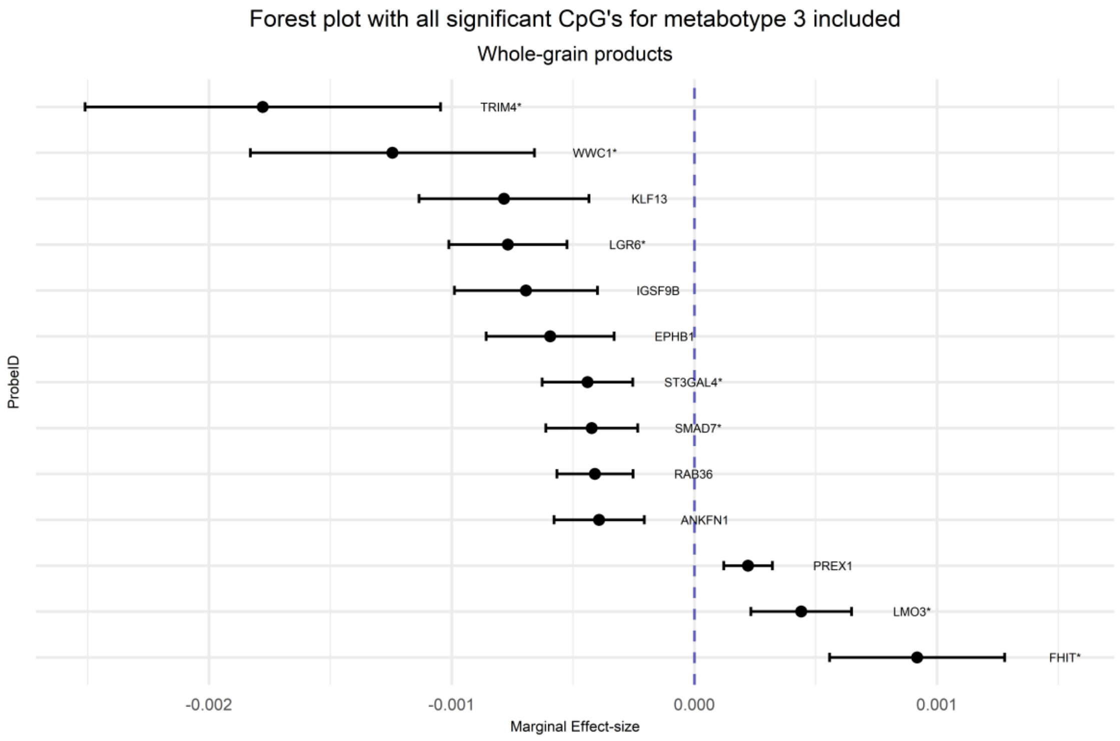

Supplement: Supplementary file 1 [file life-12-01064-s001.zip › life-1794131-supplementary/Suppl_plots/Forest-plots-FigureS39-S42_Supplementary Material.pdf]
