# Supplementary material for: Association between Usual Dietary Intake of Food Groups and DNA Methylation and Effect Modification by Metabotype in the KORA FF4 Cohort
Source: Life (Basel). 2022 Jul 15;12(7):1064. doi: 10.3390/life12071064 (PMC9318948; doi:10.3390/life12071064)

Volcano Plots Figures 52-56: A: Q-Q-plot of unaltered p-values (respective metabotype). **B:** Histogram of T-statistics (respective metabotype) before and after genomic inflation correction with the bacon package. **C:** Histogram of p-values (respective metabotype) before and after genomic inflation correction with the bacon package.

Figure S52

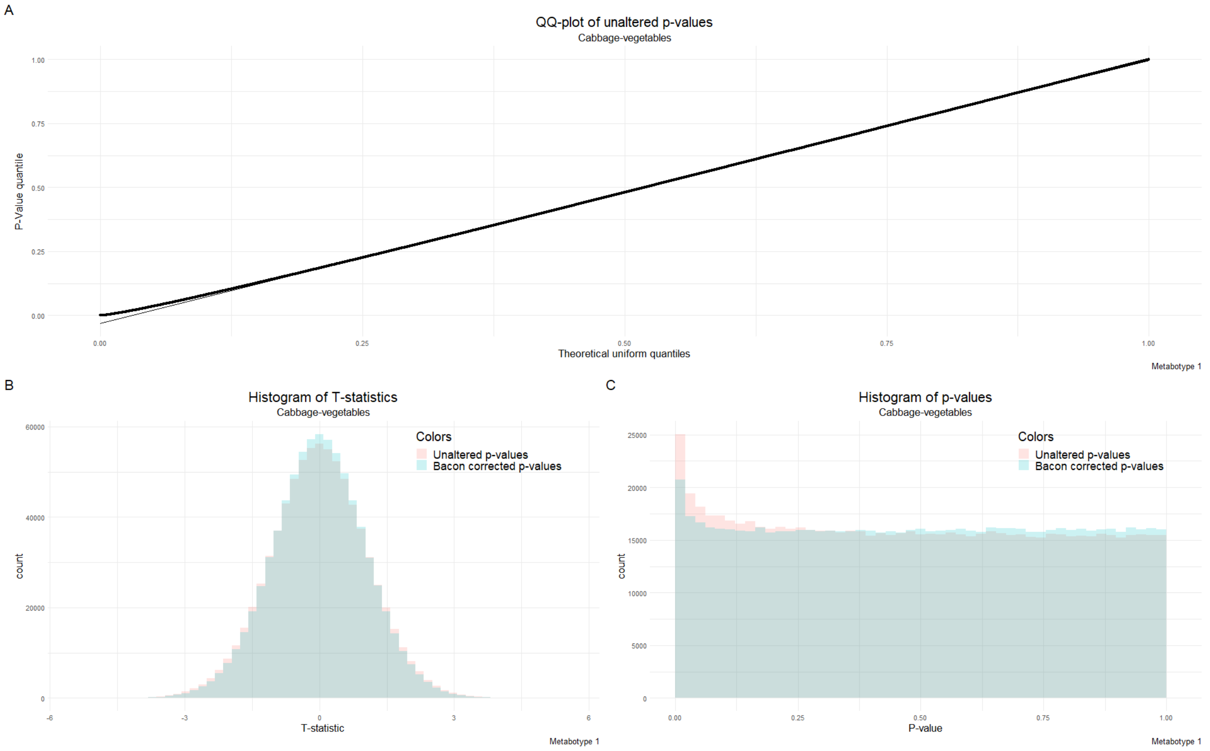

Figure S53

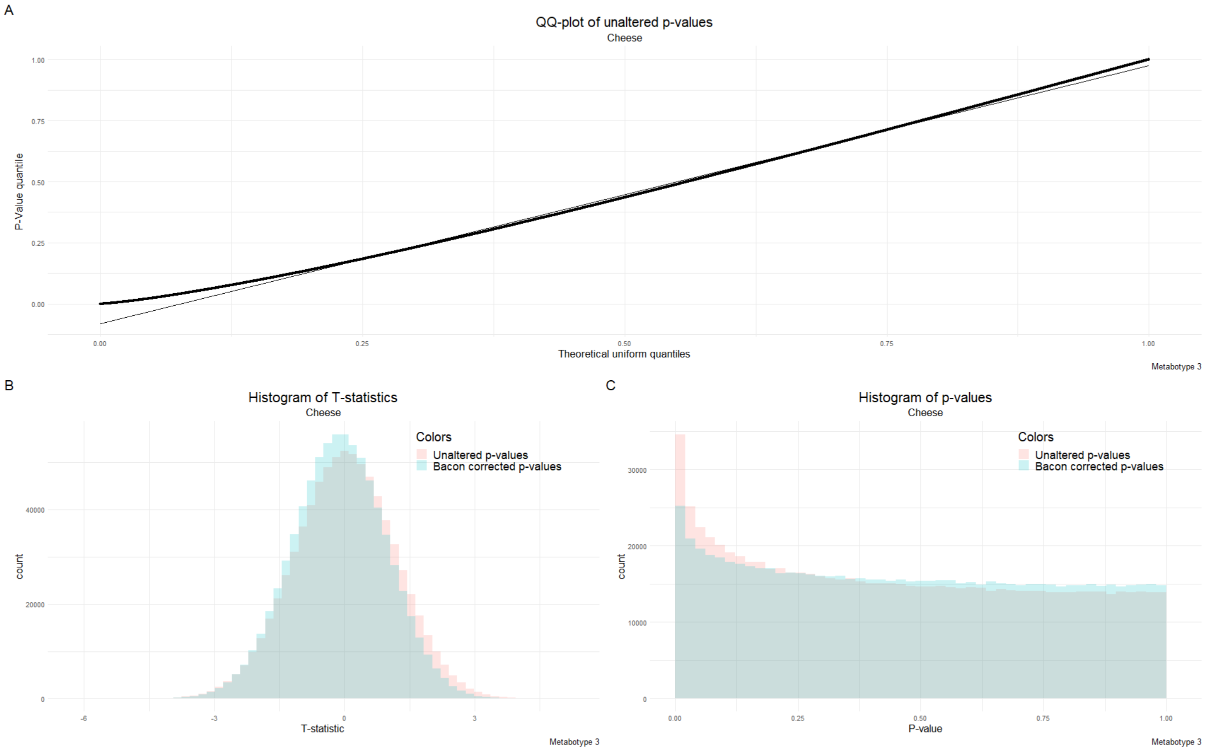

Figure S54

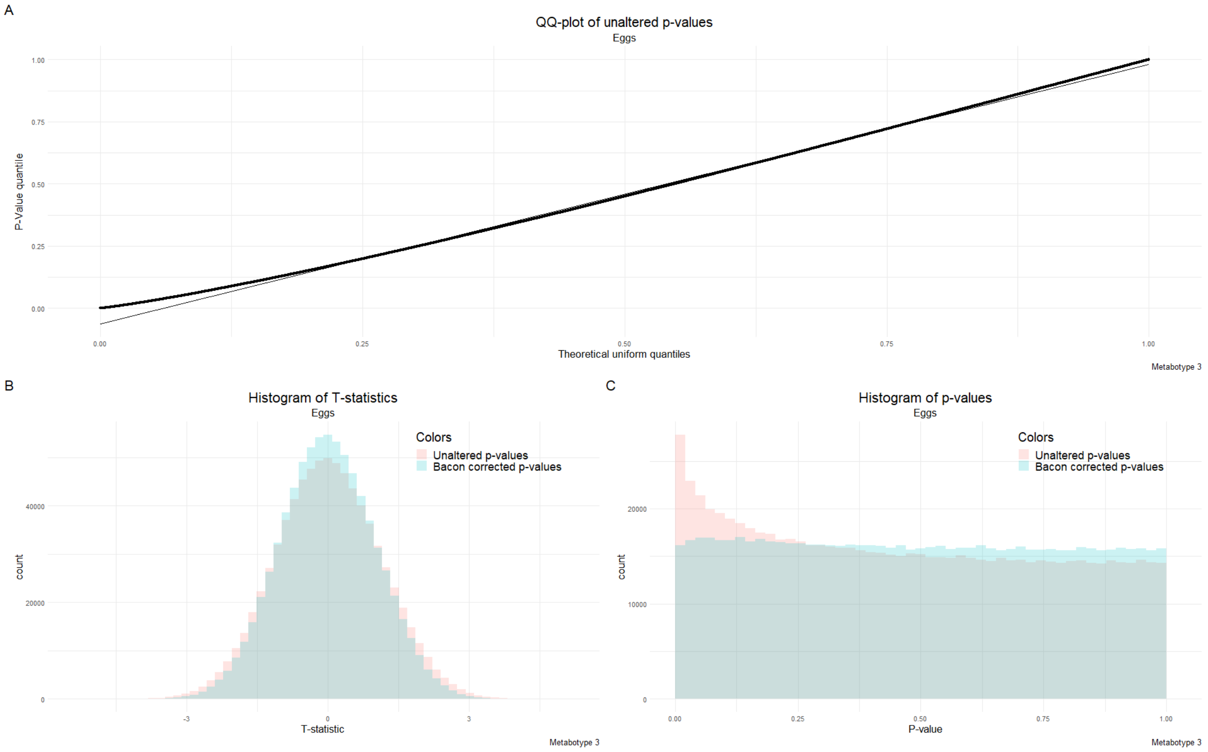

Figure S55

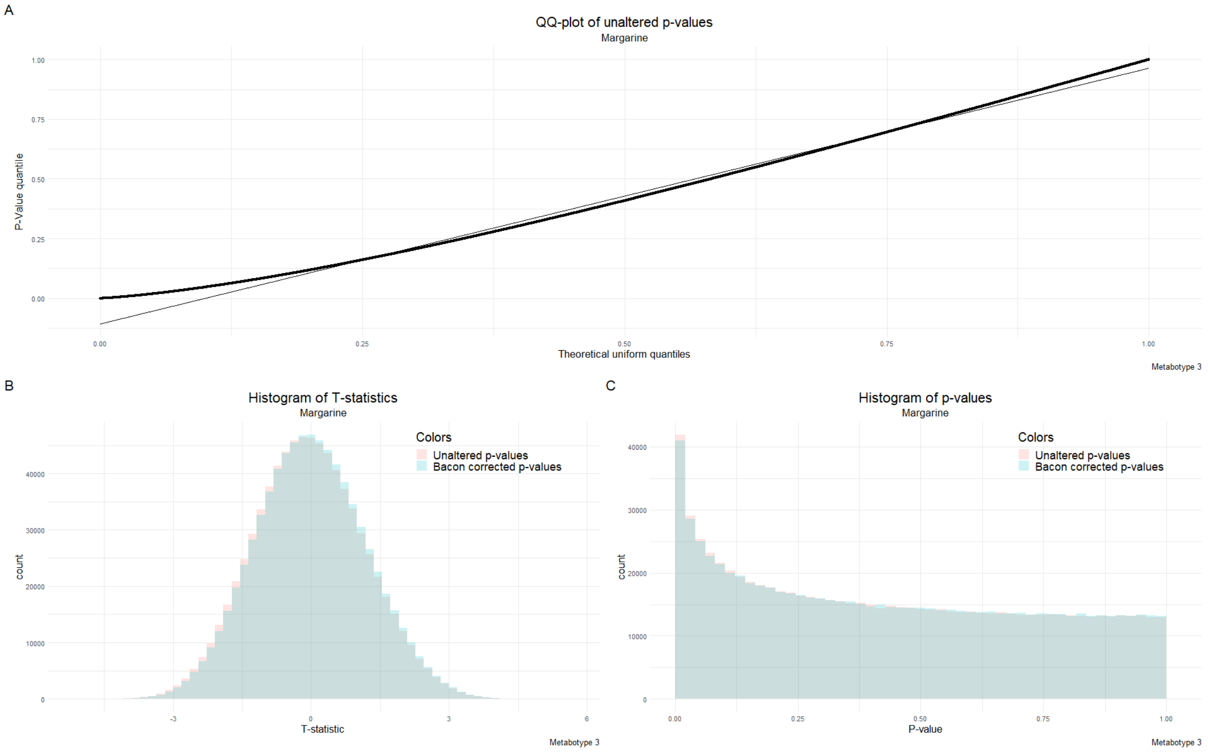

Figure S56

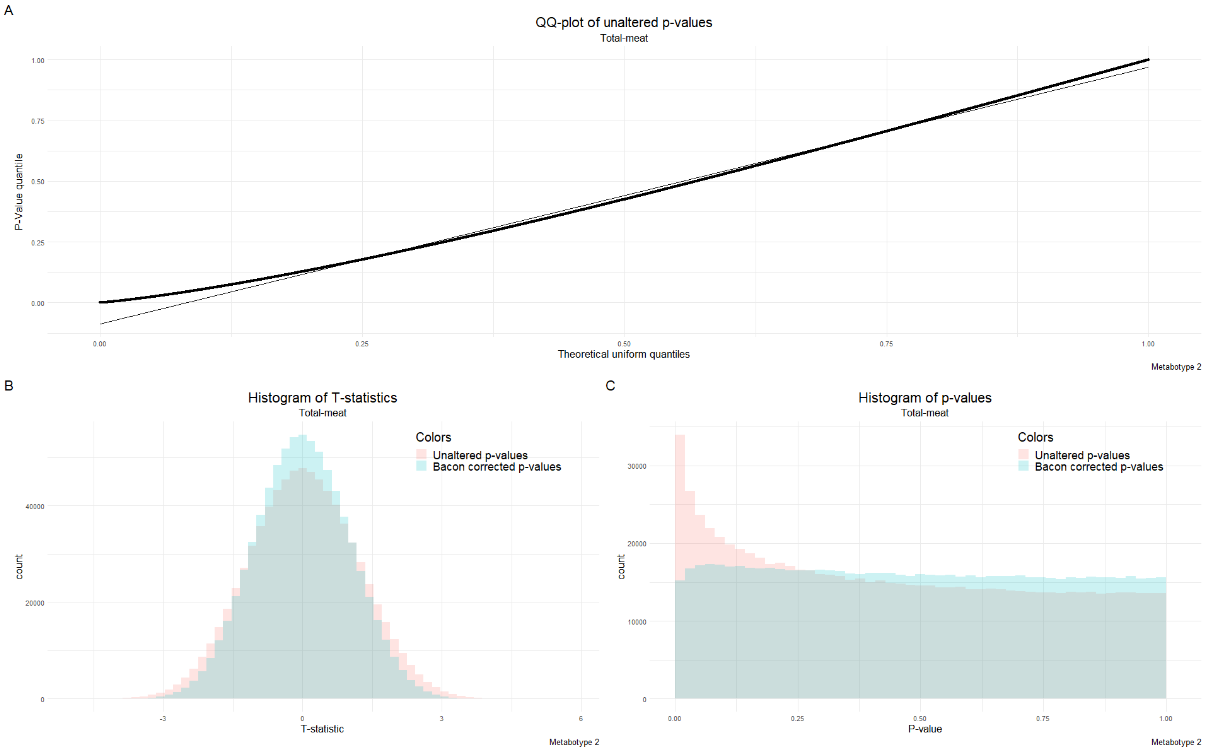

Figure S57

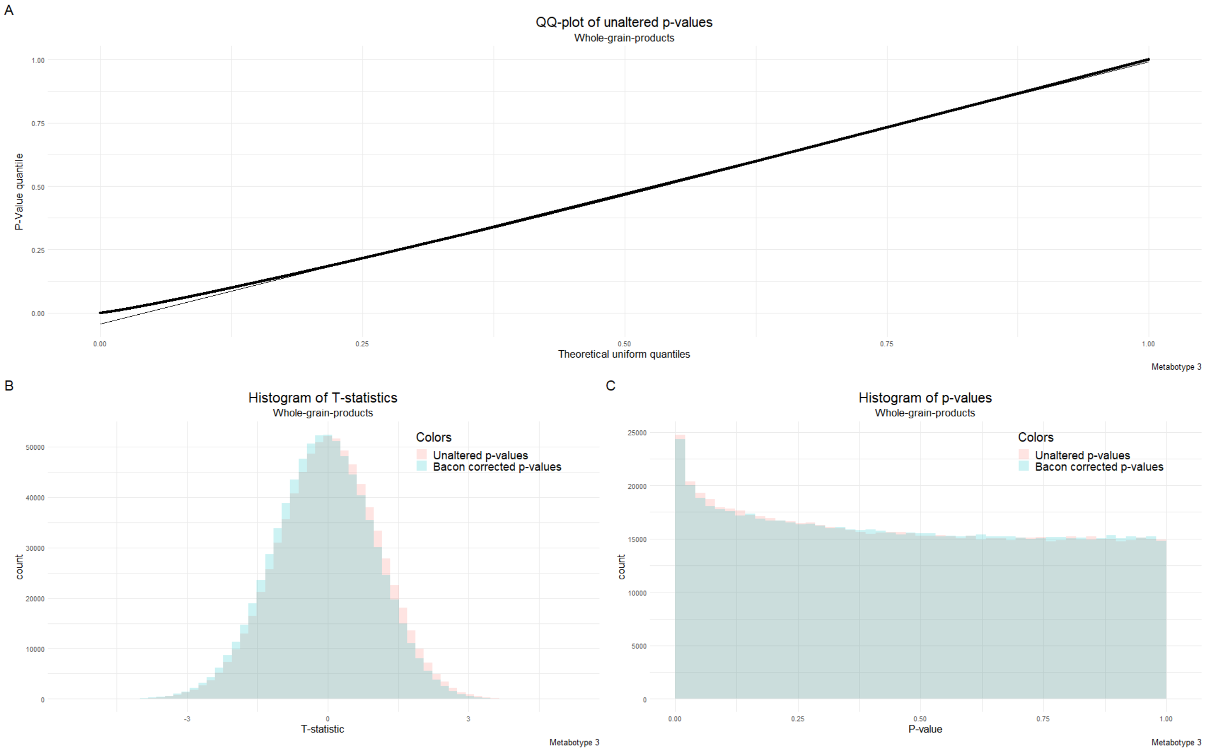

Supplement: Supplementary file 1 [file life-12-01064-s001.zip › life-1794131-supplementary/Suppl_plots/Genomic-inflation-FiguresS52-S57_Supplementary Material.pdf]
