# Supplementary material for: Association between Usual Dietary Intake of Food Groups and DNA Methylation and Effect Modification by Metabotype in the KORA FF4 Cohort
Source: Life (Basel). 2022 Jul 15;12(7):1064. doi: 10.3390/life12071064 (PMC9318948; doi:10.3390/life12071064)
Supplement: Supplementary file 1 [file life-12-01064-s001.zip › life-1794131-supplementary/Suppl_plots/Interaction-plots-FiguresS43-S51_Supplementary Material.pdf]

Interaction plot description 43-51: Y-axis indicates the predicted methylation level based on calculated marginal effect size based on emtrends() function in the emmeans package, given metabotype. X-Axis indicates respective food group consumption as residuals and is the same for the histogram and the interaction plot. Interpretability is possible as, how many grams of respective food group you eat more than the average for your given calorie consumption. Marginal histograms show the distribution of the variable plotted on the X-Axis. Marginal Histograms show the distribution of the variable plotted on the X-Axis.

Interaction plots 43-46– selected genes: Shown are interactions plots with marginal histograms for TLR5, CPT2, PON3 and RXRG.

Interaction plots 47-51- common genes: Shown are interaction plots with marginal histograms for annotated genes that were significant multiple times across different or within the same food group.

Figure S43

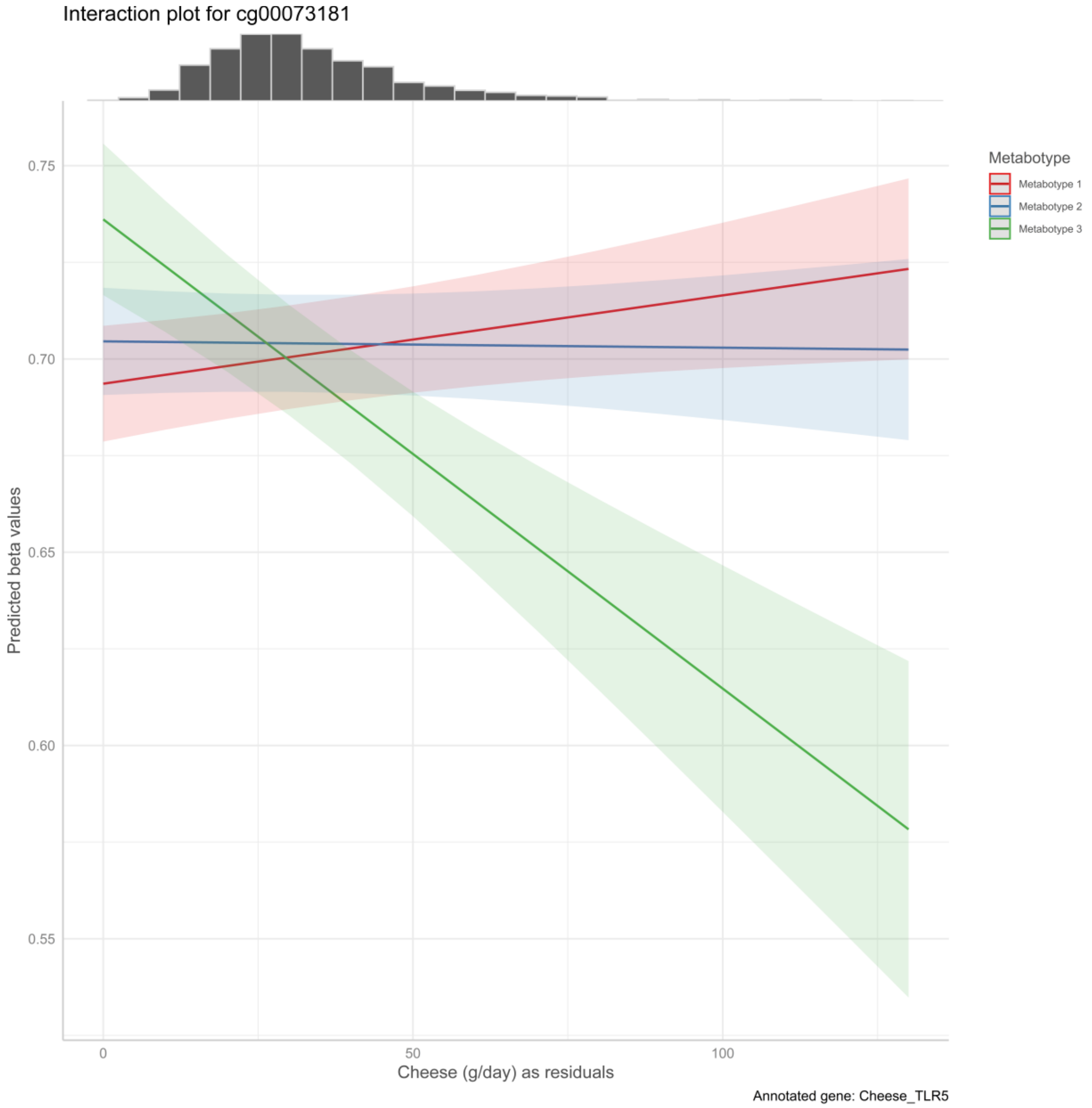

Figure S44

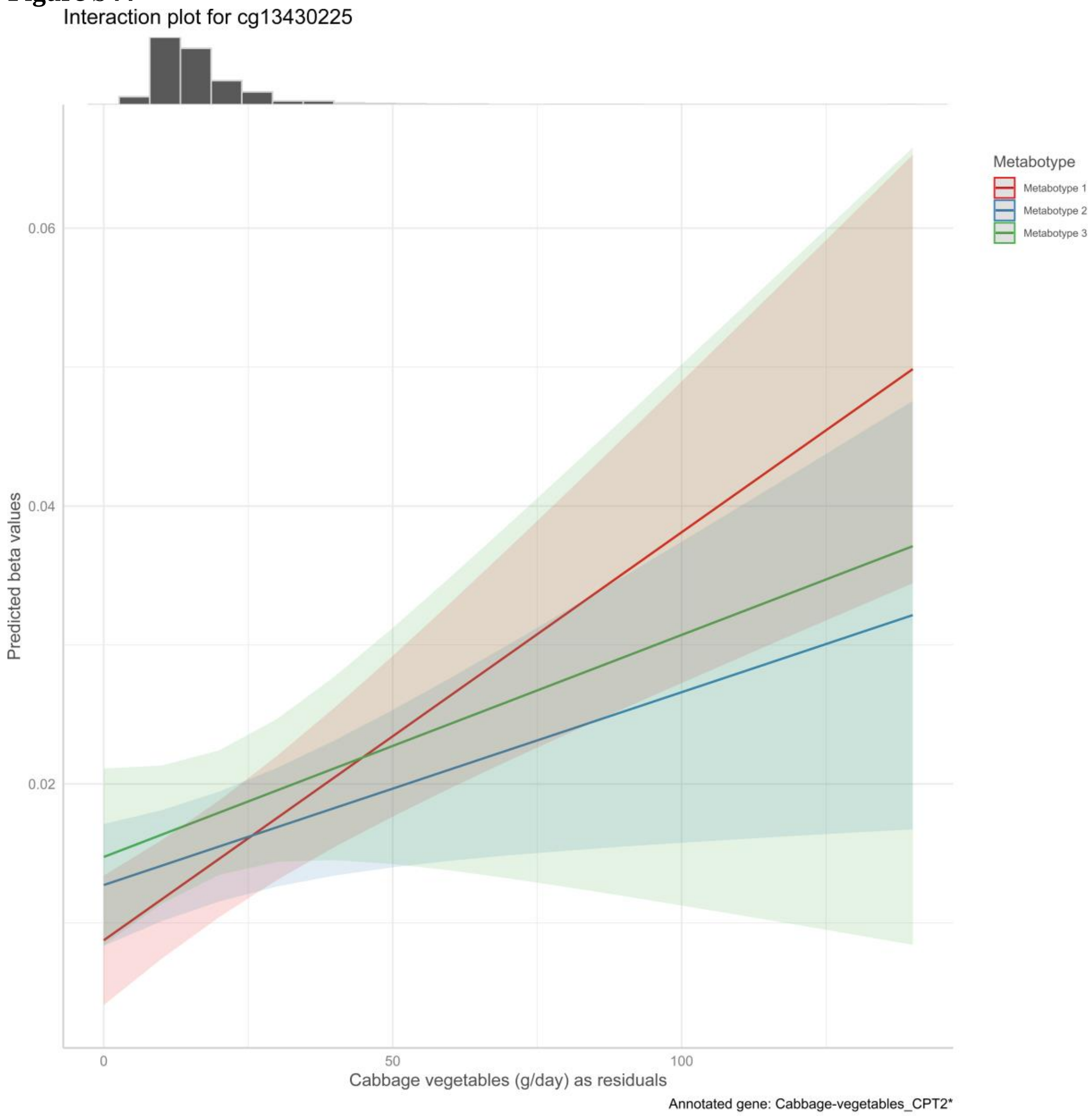
Interaction plot for cg13430225
Metabotype
Metabotype 1
Metabotype 2
Metabotype 3
0.06
0.04
0.02
Predicted beta values
0
50
100
Cabbage vegetables (g/day) as residuals
Annotated gene: Cabbage-vegetables_CPT2*

Figure S45

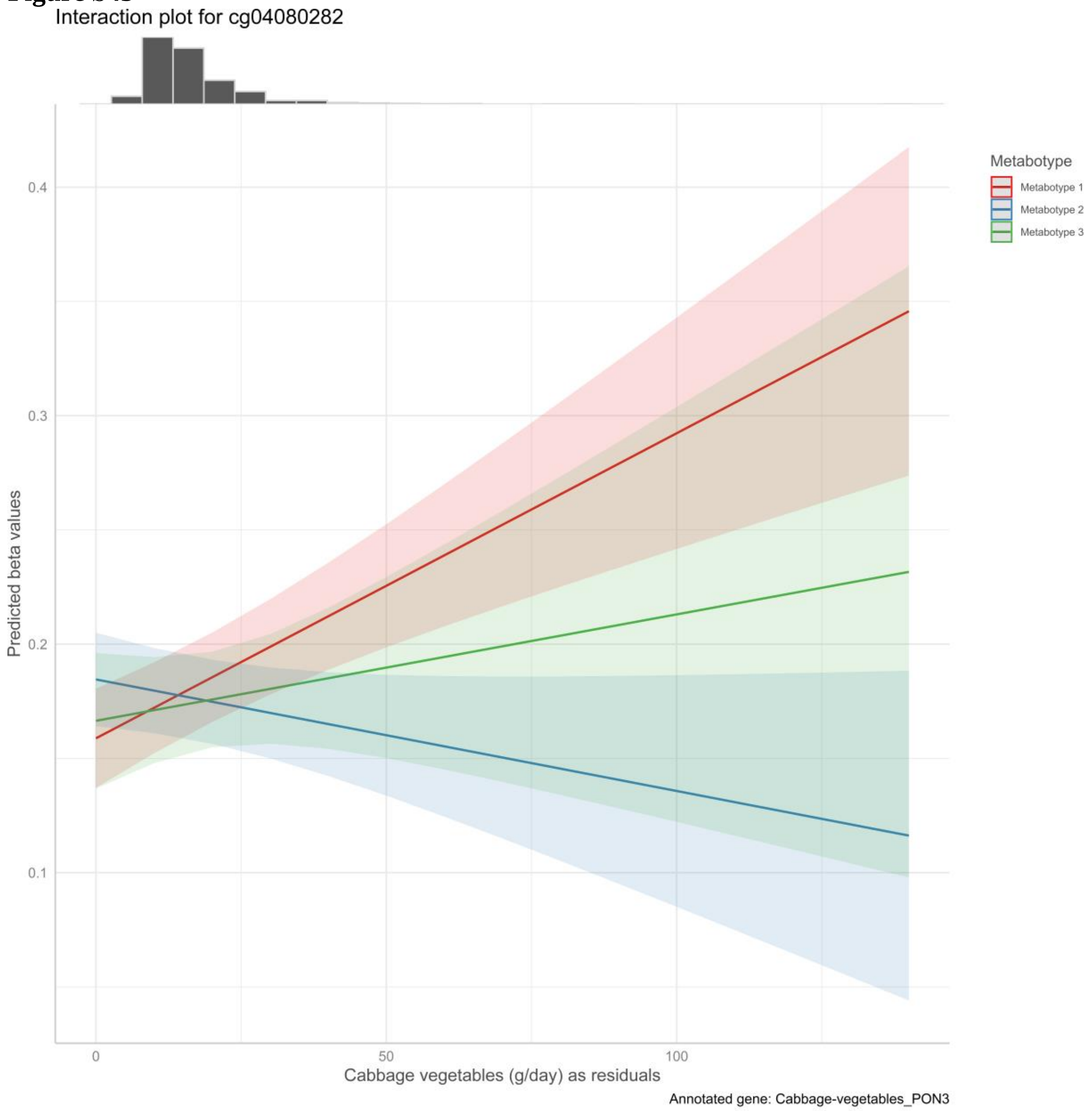
Interaction plot for cg04080282
Metabotype
Metabotype 1
Metabotype 2
Metabotype 3
Predicted beta values
0.4
0.3
0.2
0.1
0
50
100
Cabbage vegetables (g/day) as residuals
Annotated gene: Cabbage-vegetables_PON3

Figure S46

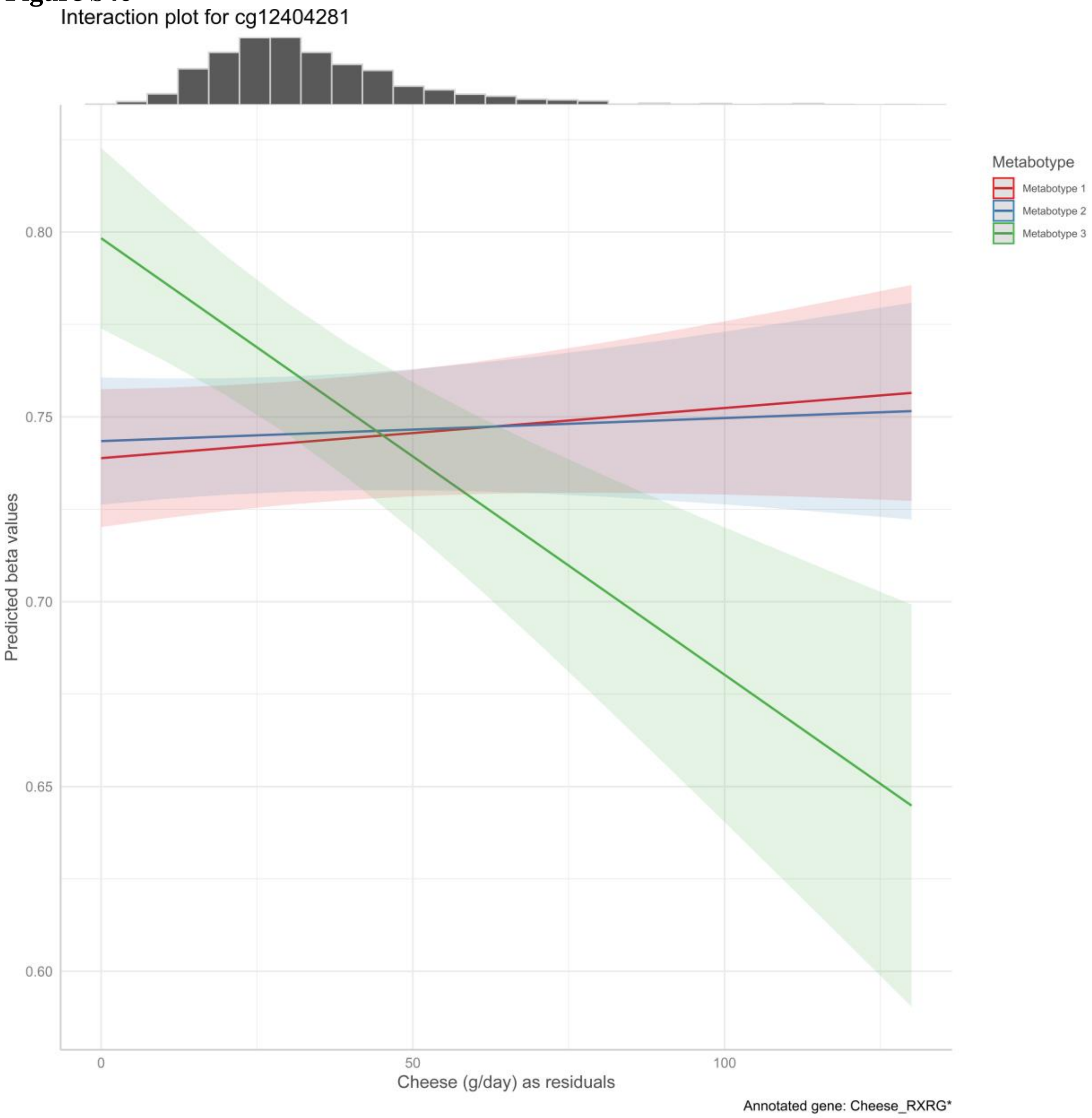
Interaction plot for cg12404281
Metabotype
Metabotype 1
Metabotype 2
Metabotype 3
0.80
0.75
0.70
0.65
0.60
Predicted beta values
0
50
100
Cheese (g/day) as residuals
Annotated gene: Cheese_RXRG*

Figure S47

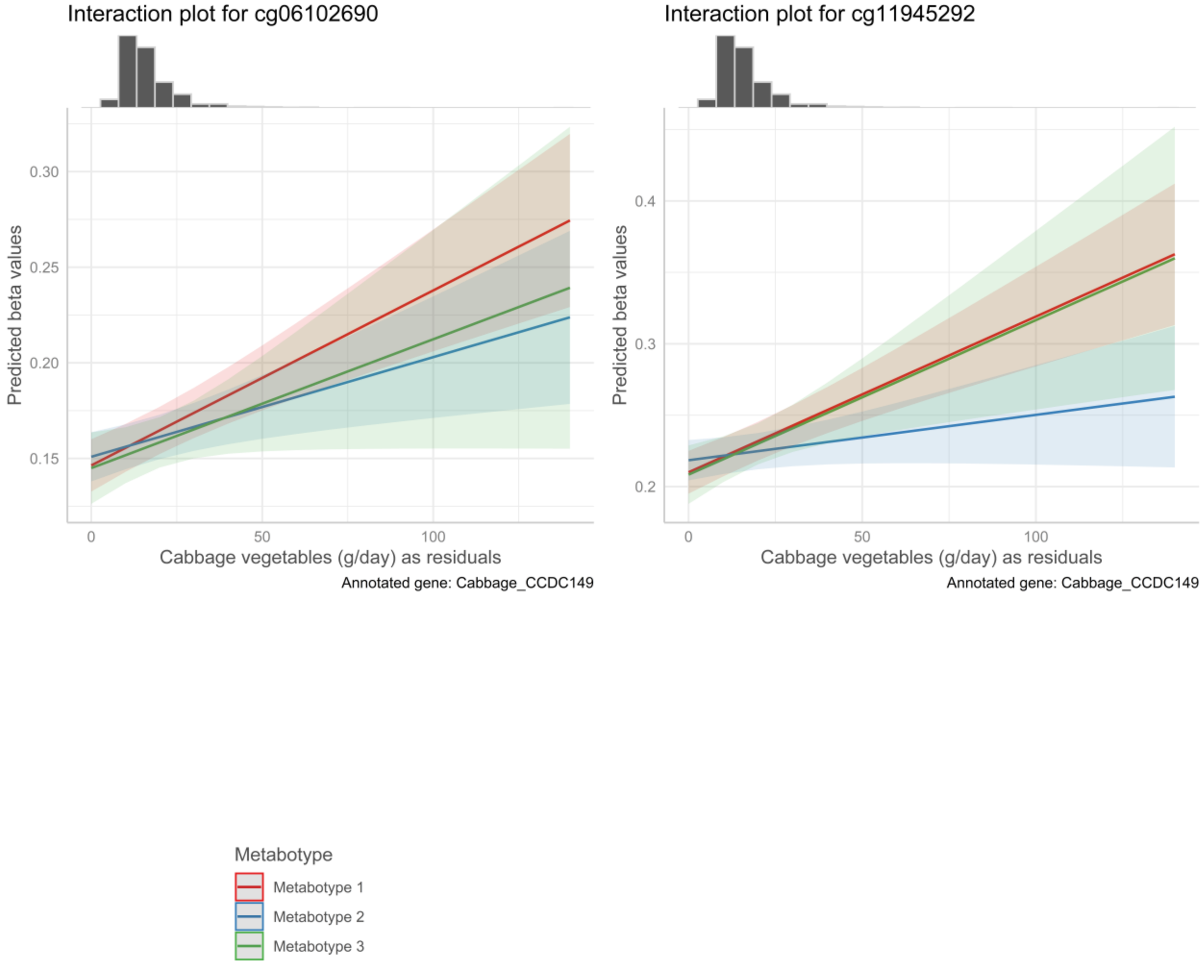

Interaction plot for cg06102690
Predicted beta values
0.30
0.25
0.20
0.15
0
50
100
Cabbage vegetables (g/day) as residuals
Annotated gene: Cabbage_CCDC149
Interaction plot for cg11945292
Predicted beta values
0.4
0.3
0.2
0
50
100
Cabbage vegetables (g/day) as residuals
Annotated gene: Cabbage_CCDC149
Metabotype
Metabotype 1
Metabotype 2
Metabotype 3

Figure S48

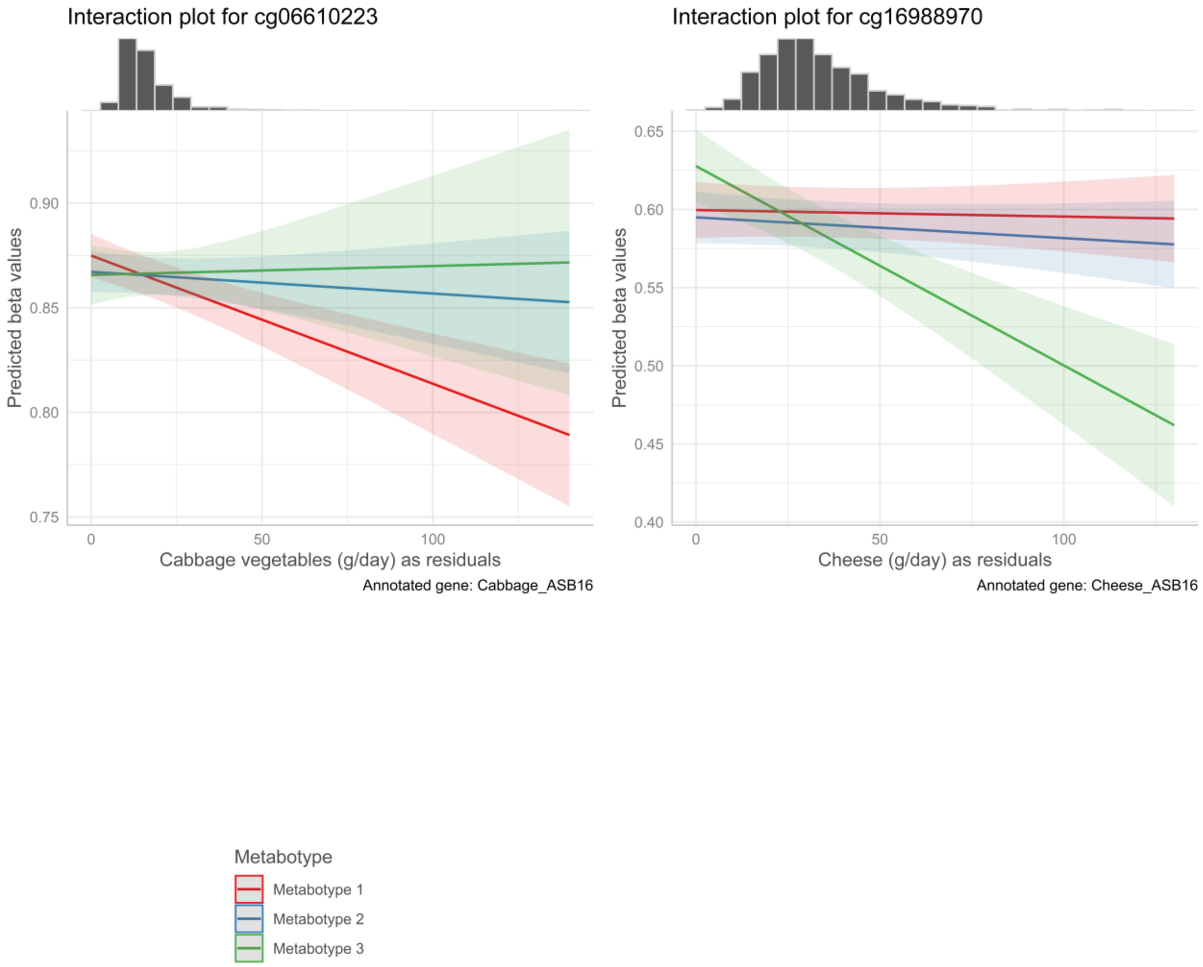
Interaction plot for cg06610223
Predicted beta values
0.90
0.85
0.80
0.75
0
50
100
Cabbage vegetables (g/day) as residuals
Annotated gene: Cabbage_ASB16
Interaction plot for cg16988970
Predicted beta values
0.65
0.60
0.55
0.50
0.45
0.40
0
50
100
Cheese (g/day) as residuals
Annotated gene: Cheese_ASB16
Metabotype
Metabotype 1
Metabotype 2
Metabotype 3

Figure S49

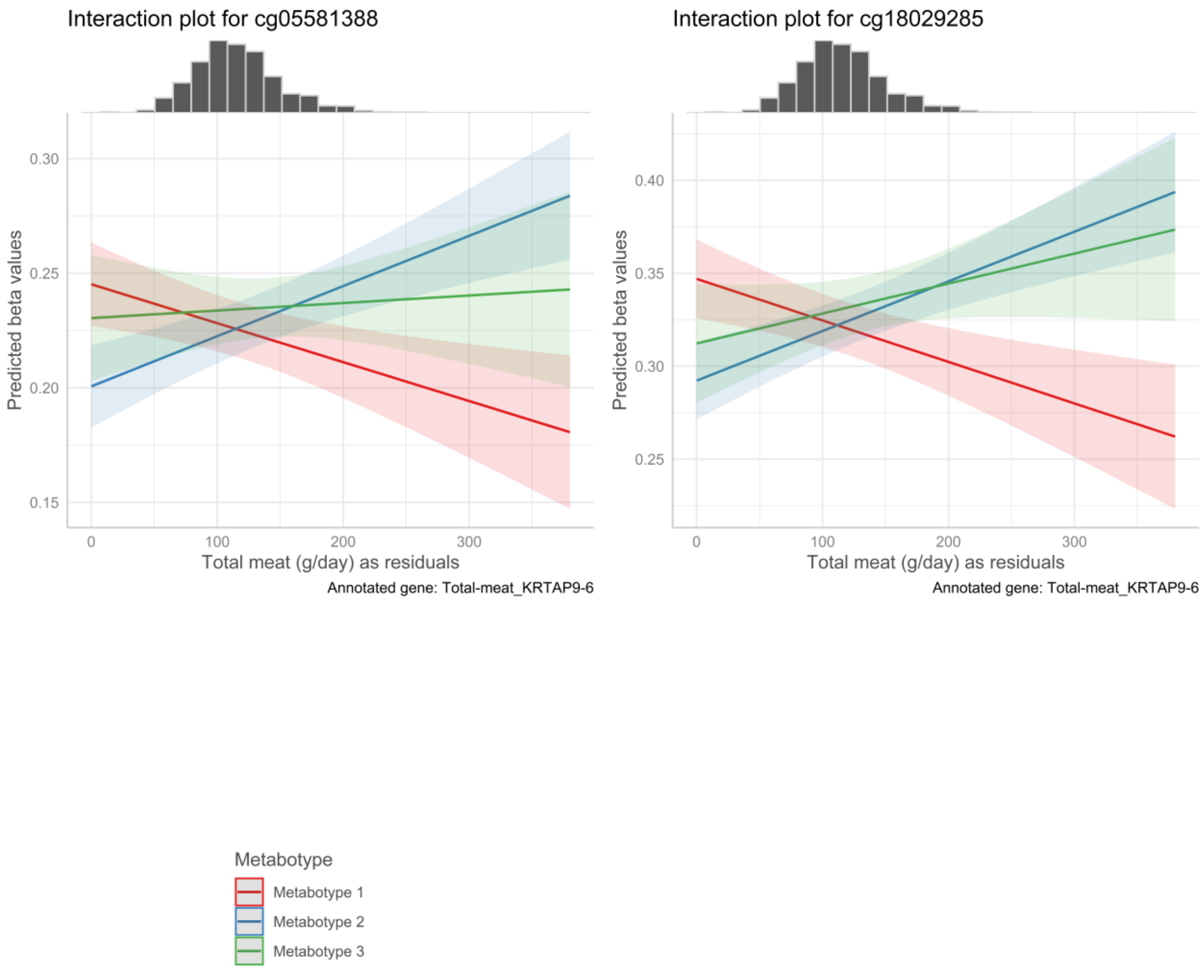
Interaction plot for cg05581388
Interaction plot for cg18029285
Predicted beta values
0.30
0.25
0.20
0.15
0.40
0.35
0.30
0.25
0
100
200
300
Total meat (g/day) as residuals
Annotated gene: Total-meat_KRTAP9-6
Metabotype
Metabotype 1
Metabotype 2
Metabotype 3

Figure S50

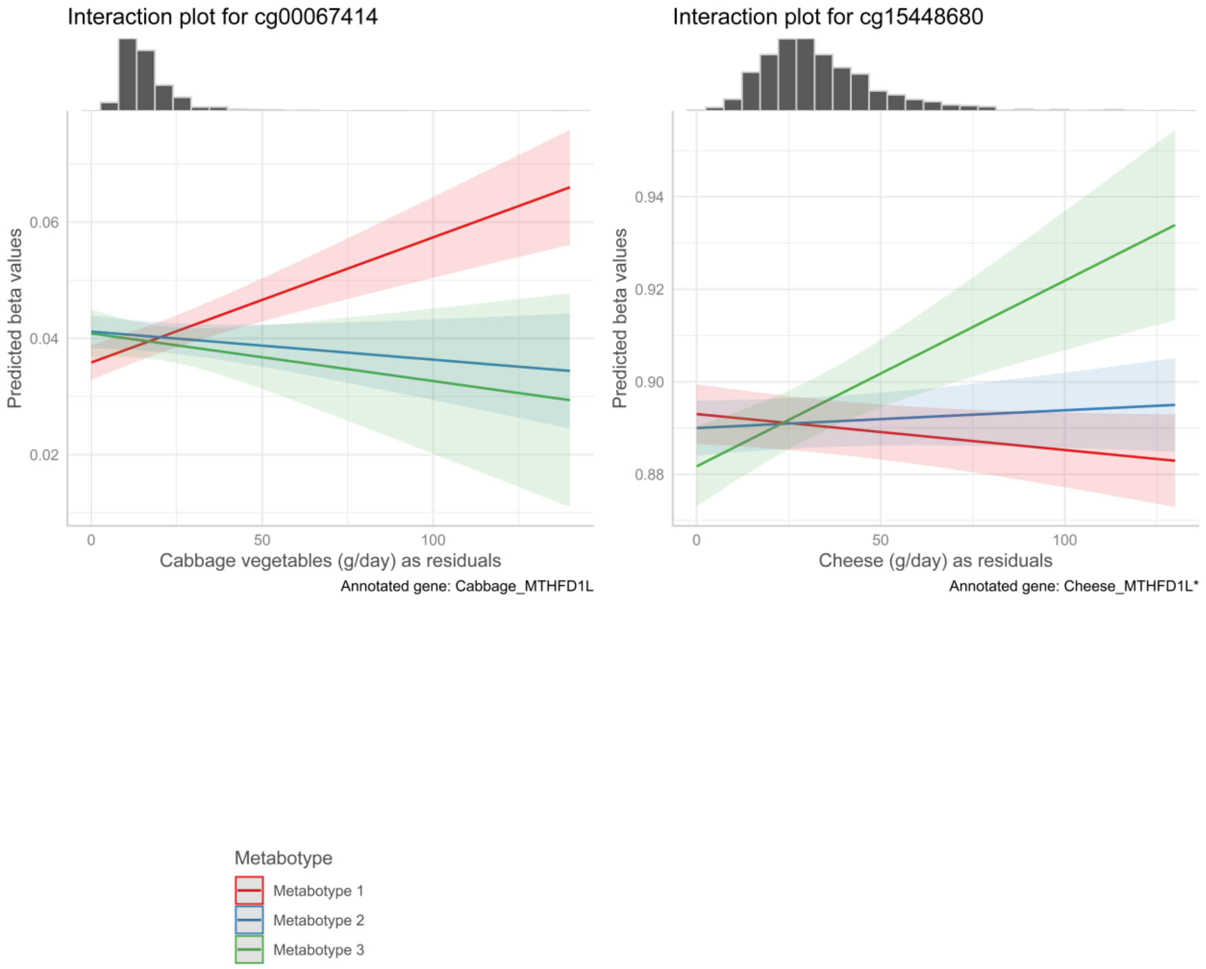
Interaction plot for cg00067414
Predicted beta values
0.02
0.04
0.06
0
50
100
Cabbage vegetables (g/day) as residuals
Annotated gene: Cabbage_MTHFD1L
Interaction plot for cg15448680
Predicted beta values
0.88
0.90
0.92
0.94
0
50
100
Cheese (g/day) as residuals
Annotated gene: Cheese_MTHFD1L*
Metabotype
Metabotype 1
Metabotype 2
Metabotype 3

Figure S51

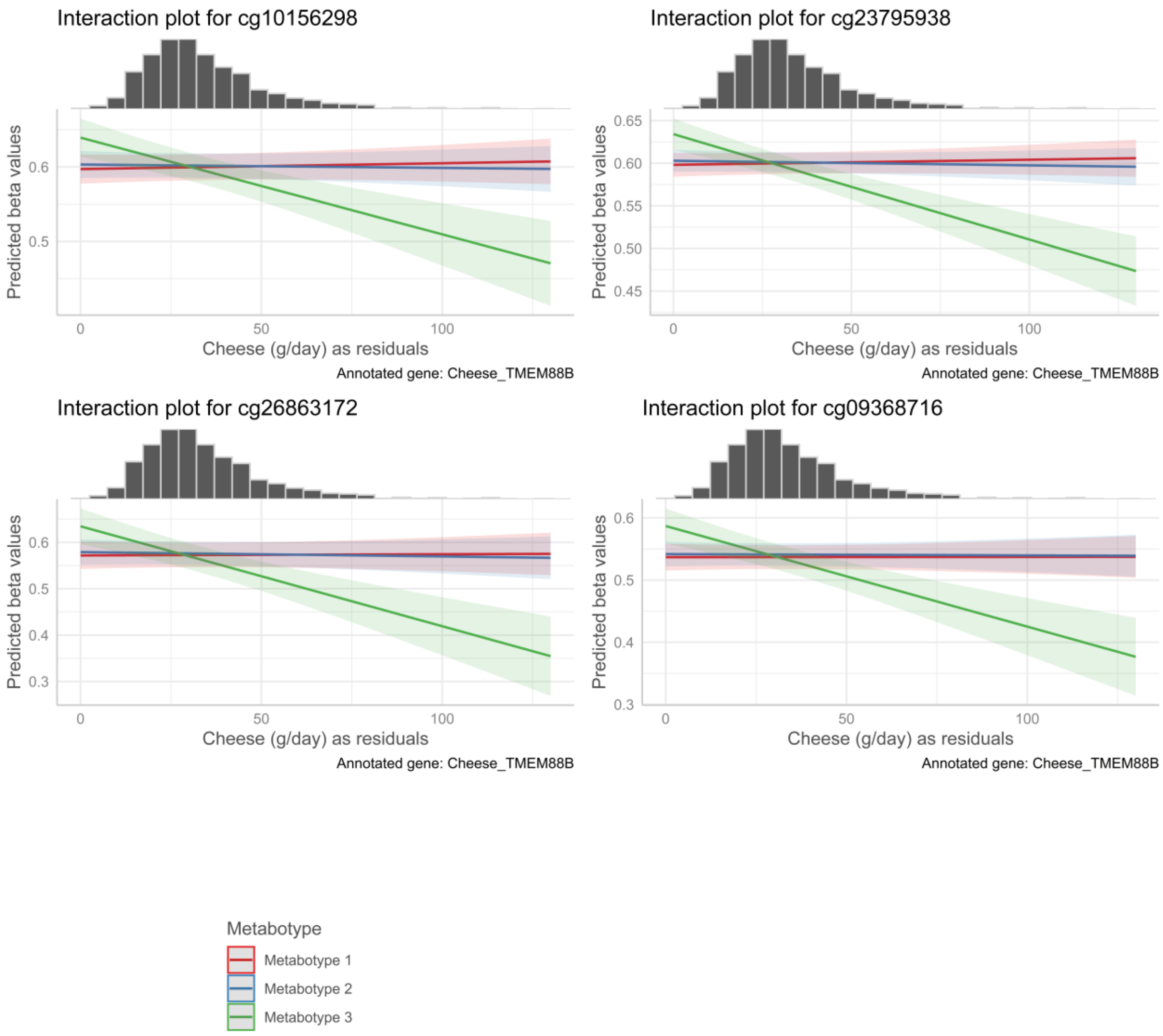
Interaction plot for cg10156298
Predicted beta values
0.6
0.5
0
50
100
Cheese (g/day) as residuals
Annotated gene: Cheese_TMEM88B
Interaction plot for cg23795938
0.65
0.60
0.55
0.50
0.45
Annotated gene: Cheese_TMEM88B
Interaction plot for cg26863172
0.6
0.5
0.4
0.3
Annotated gene: Cheese_TMEM88B
Interaction plot for cg09368716
Annotated gene: Cheese_TMEM88B
Metabotype
Metabotype 1
Metabotype 2
Metabotype 3
