# Supplementary material for: Association between Usual Dietary Intake of Food Groups and DNA Methylation and Effect Modification by Metabotype in the KORA FF4 Cohort
Source: Life (Basel). 2022 Jul 15;12(7):1064. doi: 10.3390/life12071064 (PMC9318948; doi:10.3390/life12071064)
Supplement: Supplementary file 1 [file life-12-01064-s001.zip › life-1794131-supplementary/Suppl_plots/Volcano-plots-FiguresS1-S38_Supplementary Material.pdf]

Volcano plot description: X-axis is estimated effect size and Y-axis is –log(10) p-value for every analyzed CpG in the basic model. Horizontal line indicates significance threshold. Turquoise dots indicates CpG's that exceeded the threshold. Analyzed food group is found in the subtitle of the plot.

Figure S1

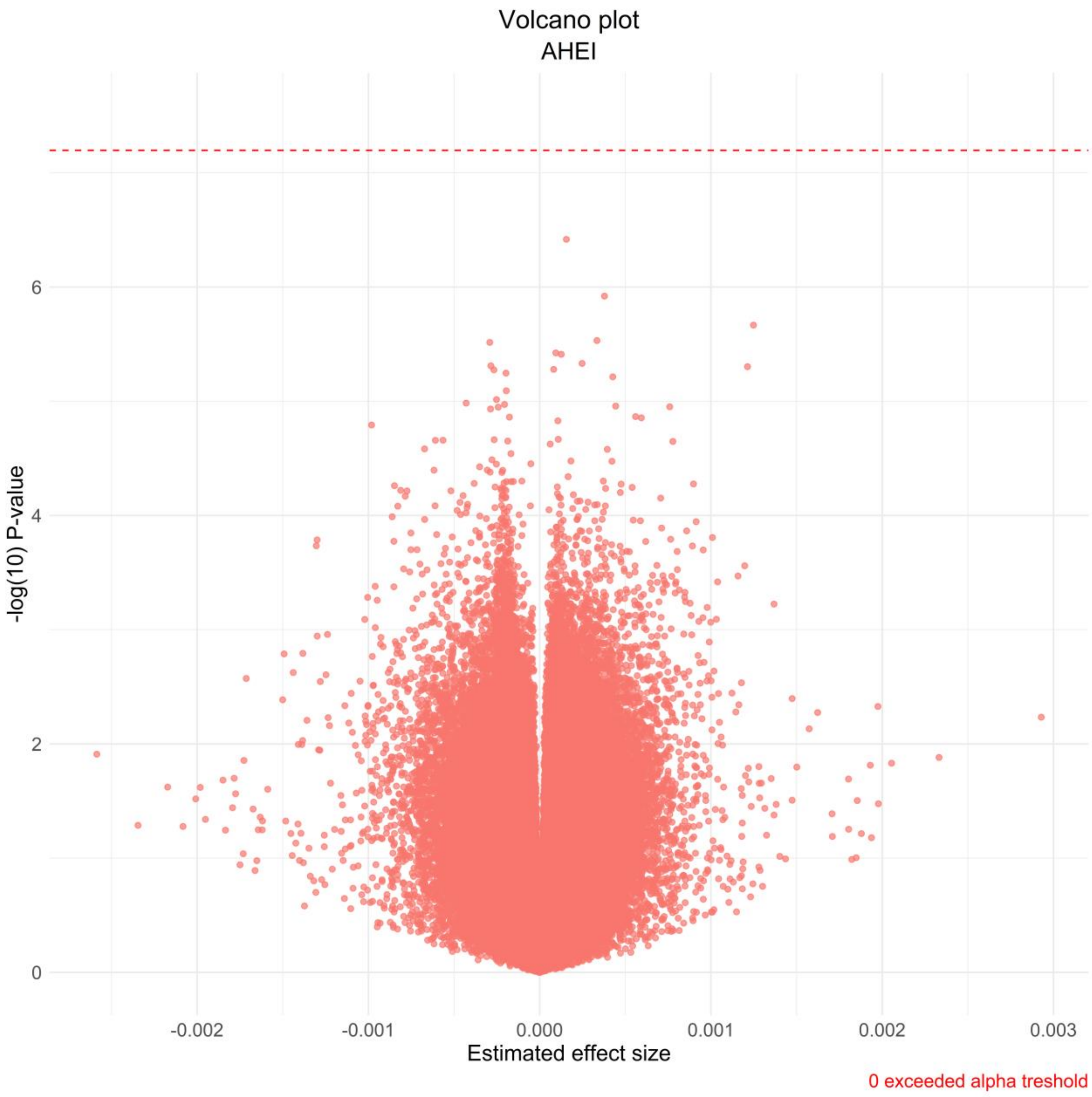

Figure S2

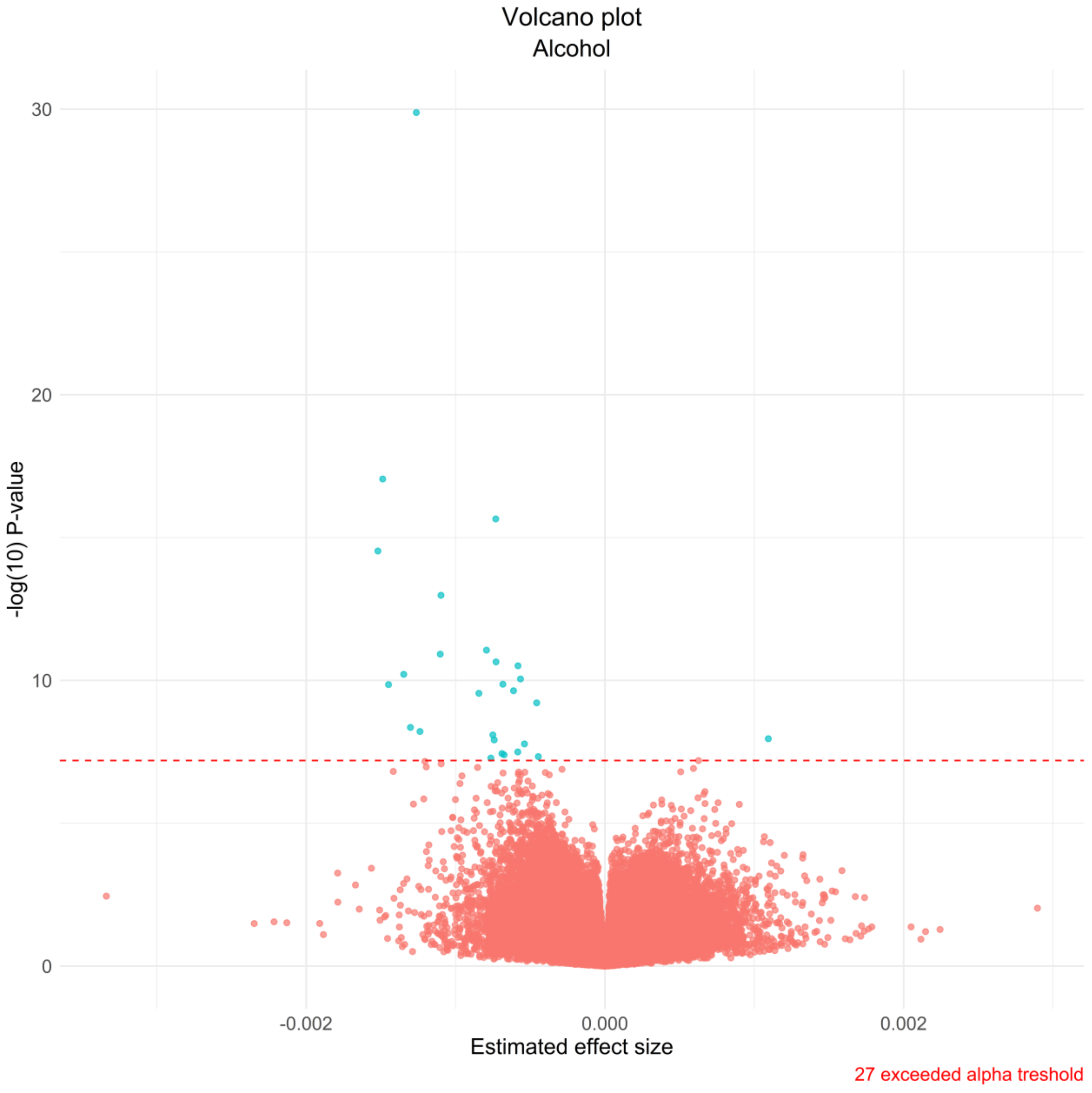

Volcano plot
Alcohol
30
20
10
0
-log(10) P-value
-0.002
0.000
0.002
Estimated effect size
27 exceeded alpha treshold

Figure S3

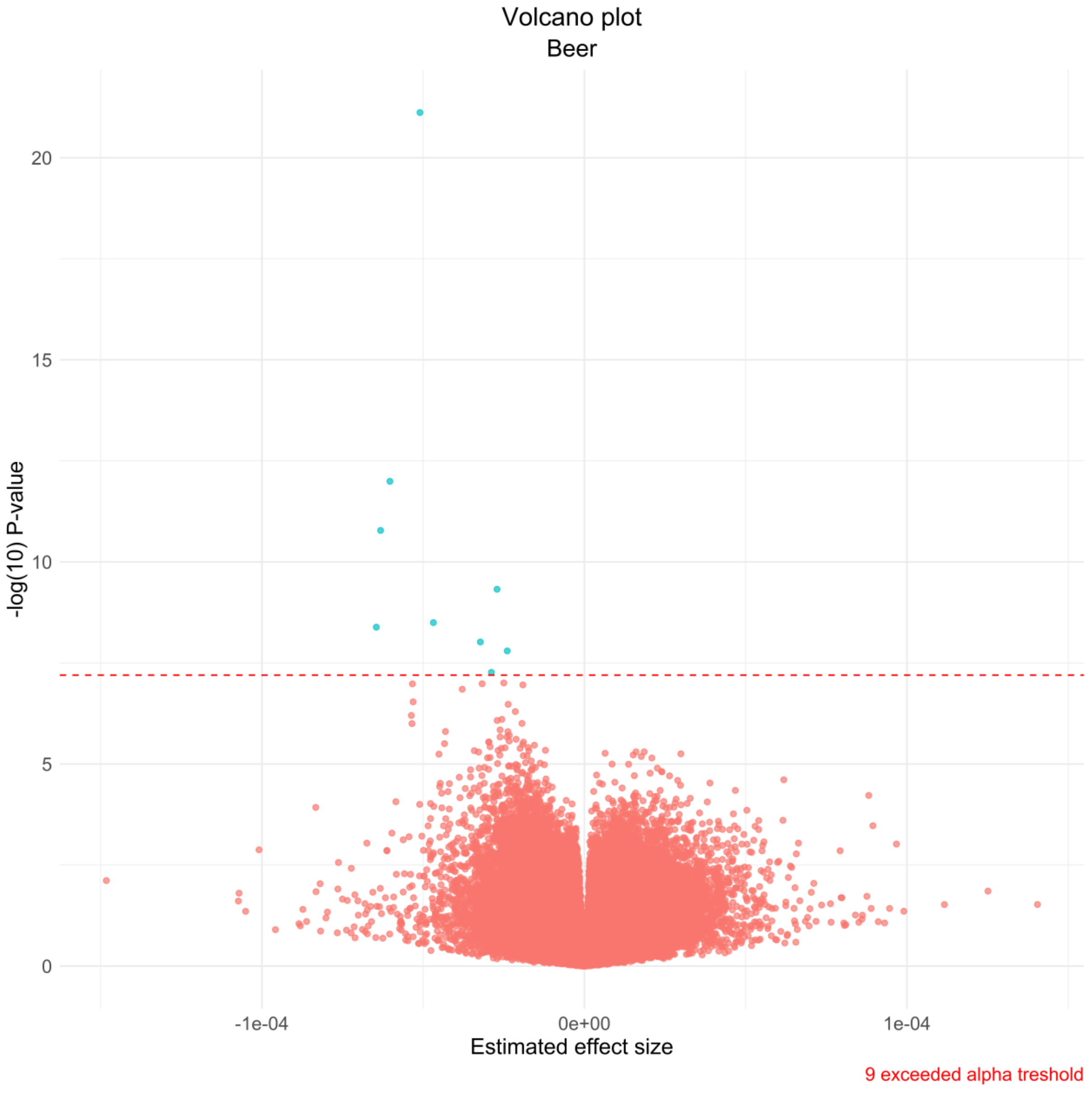
Volcano plot
Beer
20
15
10
5
0
-log(10) P-value
-1e-04
0e+00
1e-04
Estimated effect size
9 exceeded alpha treshold

Figure S4

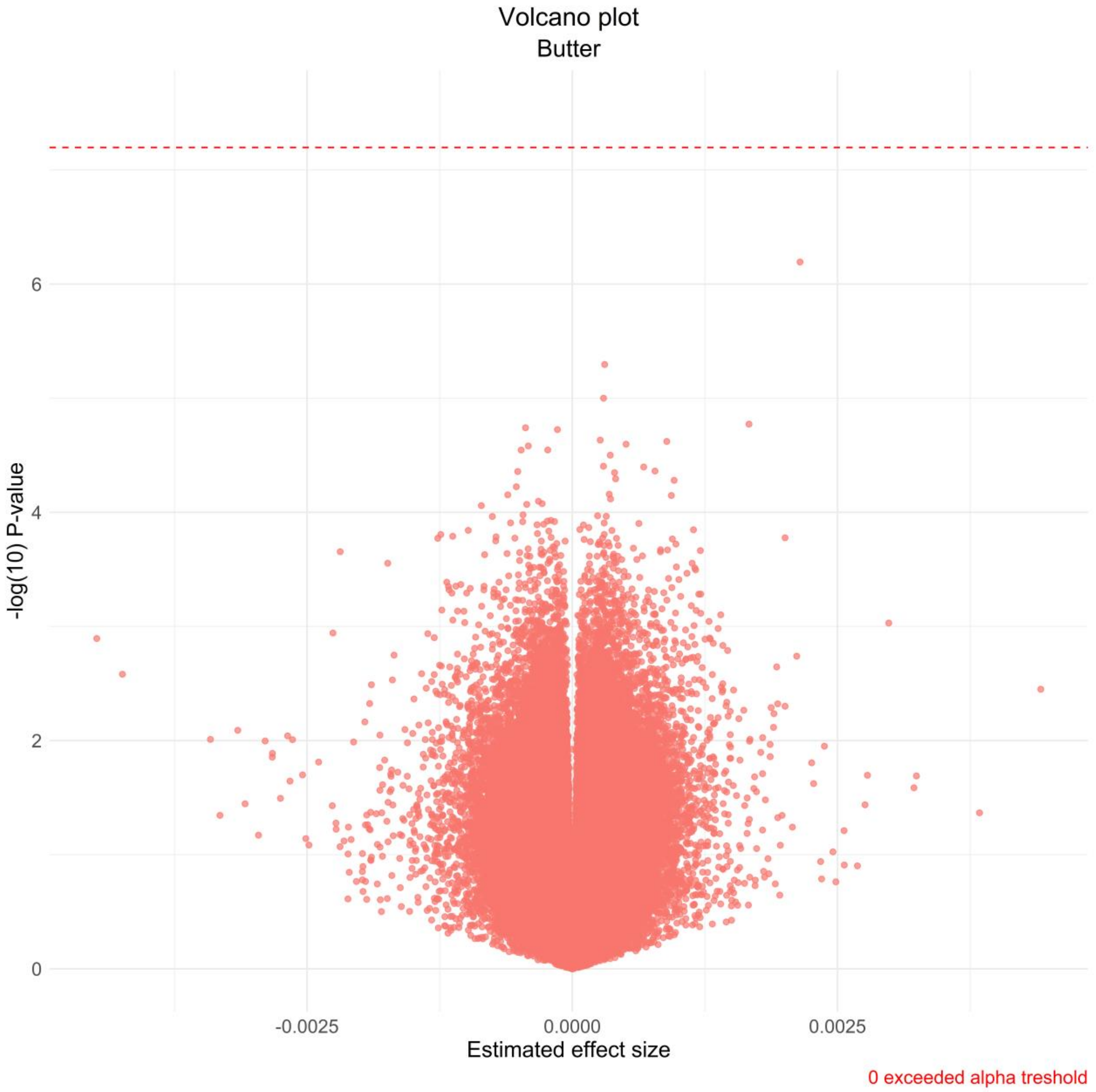

Volcano plot
Butter
6
4
2
0
-log(10) P-value
-0.0025
0.0000
0.0025
Estimated effect size
0 exceeded alpha treshold

Figure S5

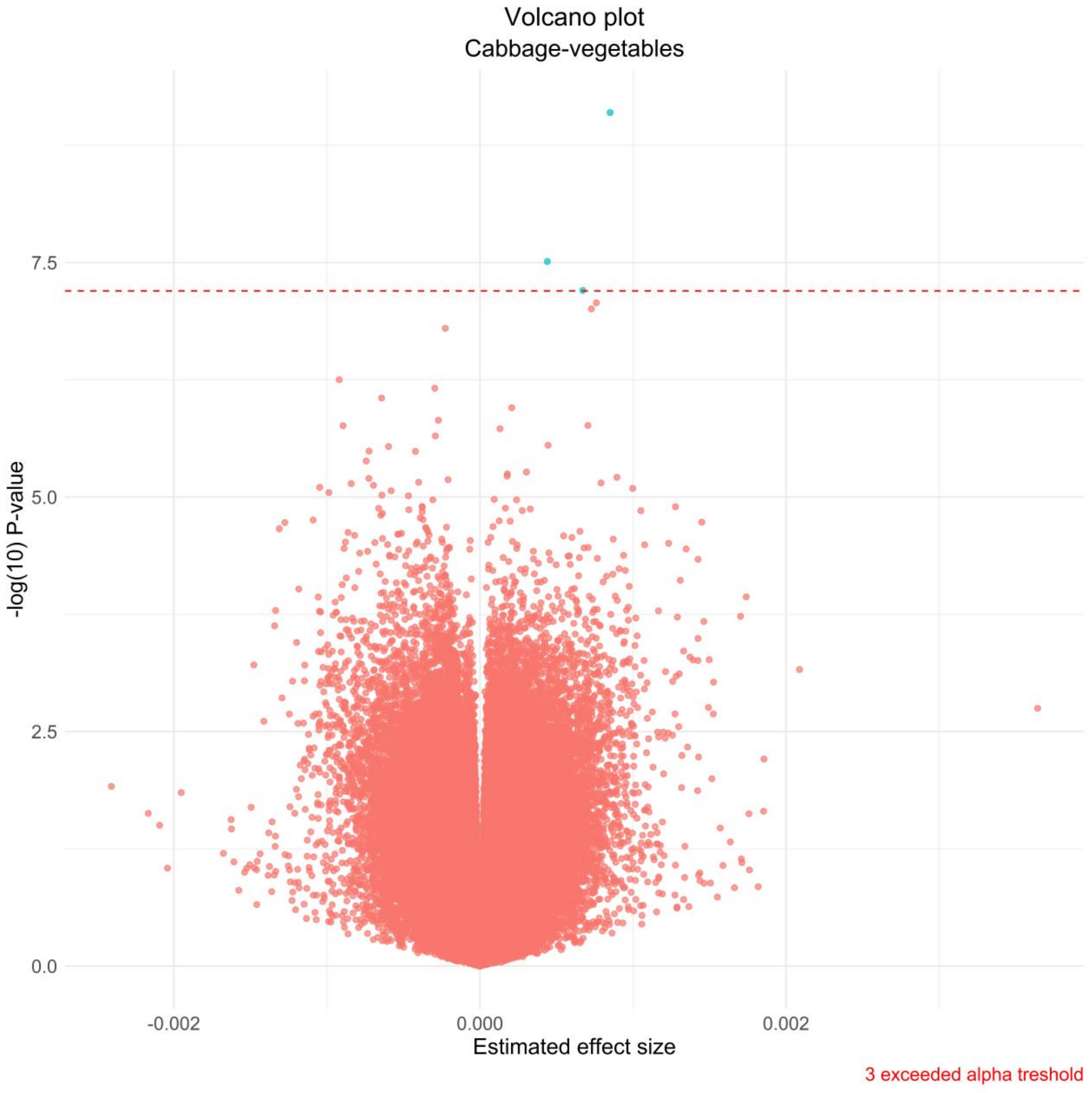
Volcano plot
Cabbage-vegetables
-log(10) P-value
0.0
2.5
5.0
7.5
-0.002
0.000
0.002
Estimated effect size
3 exceeded alpha treshold

Figure S6

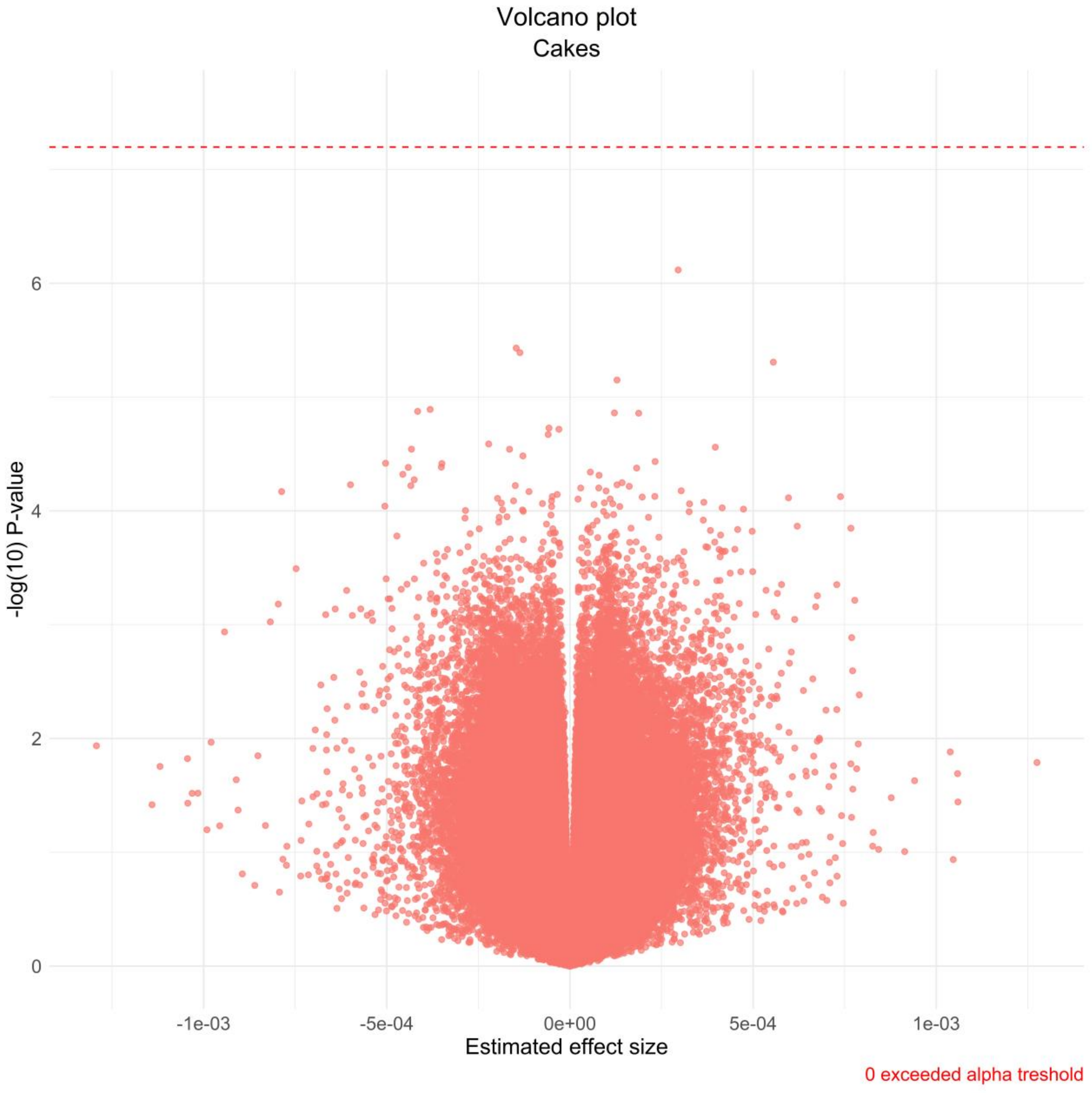

Volcano plot
Cakes
6
4
2
0
-log(10) P-value
-1e-03
-5e-04
0e+00
5e-04
1e-03
Estimated effect size
0 exceeded alpha treshold

Figure S7

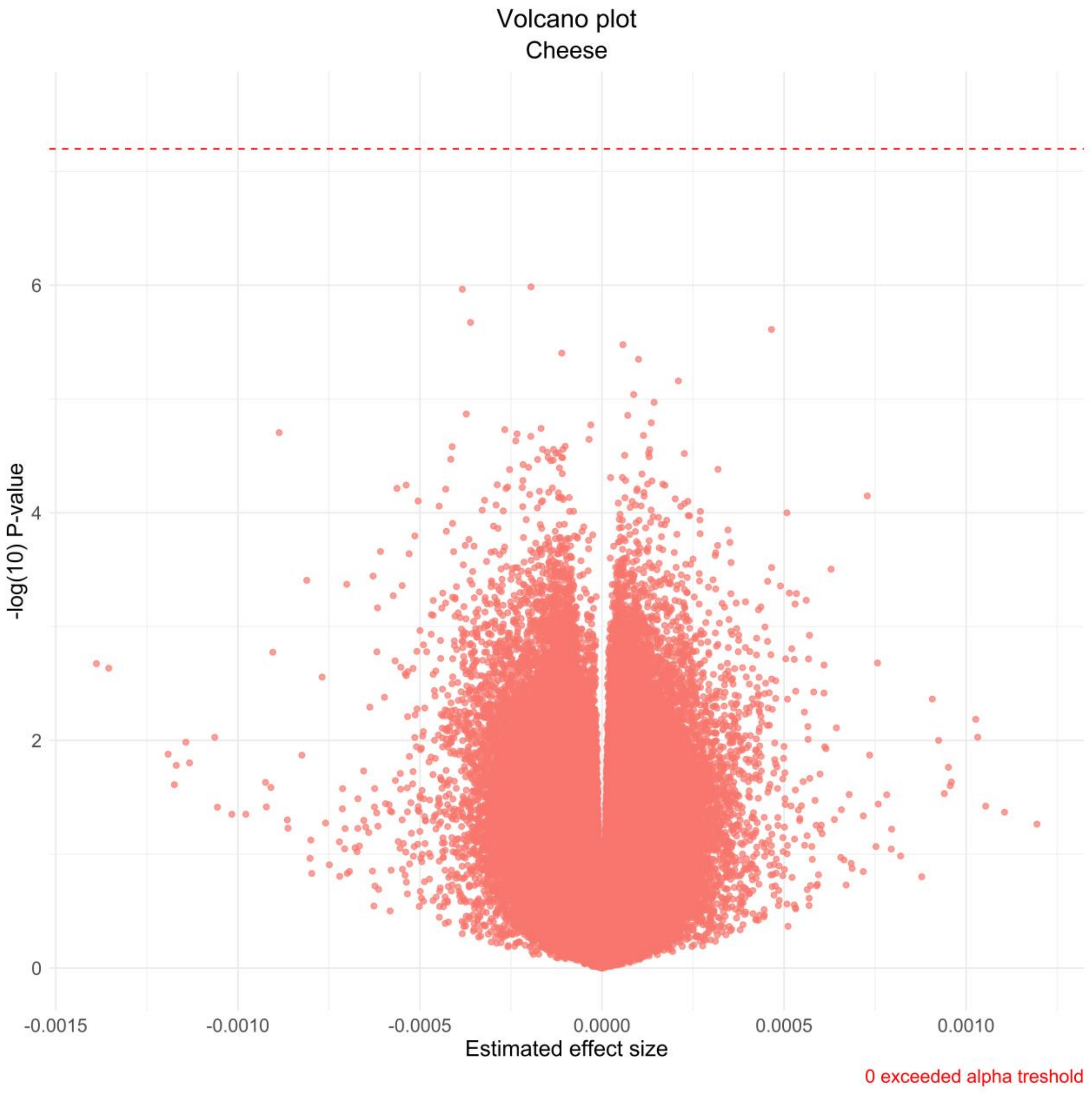

Volcano plot
Cheese
6
4
2
0
-log(10) P-value
-0.0015
-0.0010
-0.0005
0.0000
0.0005
0.0010
Estimated effect size
0 exceeded alpha treshold

Figure S8

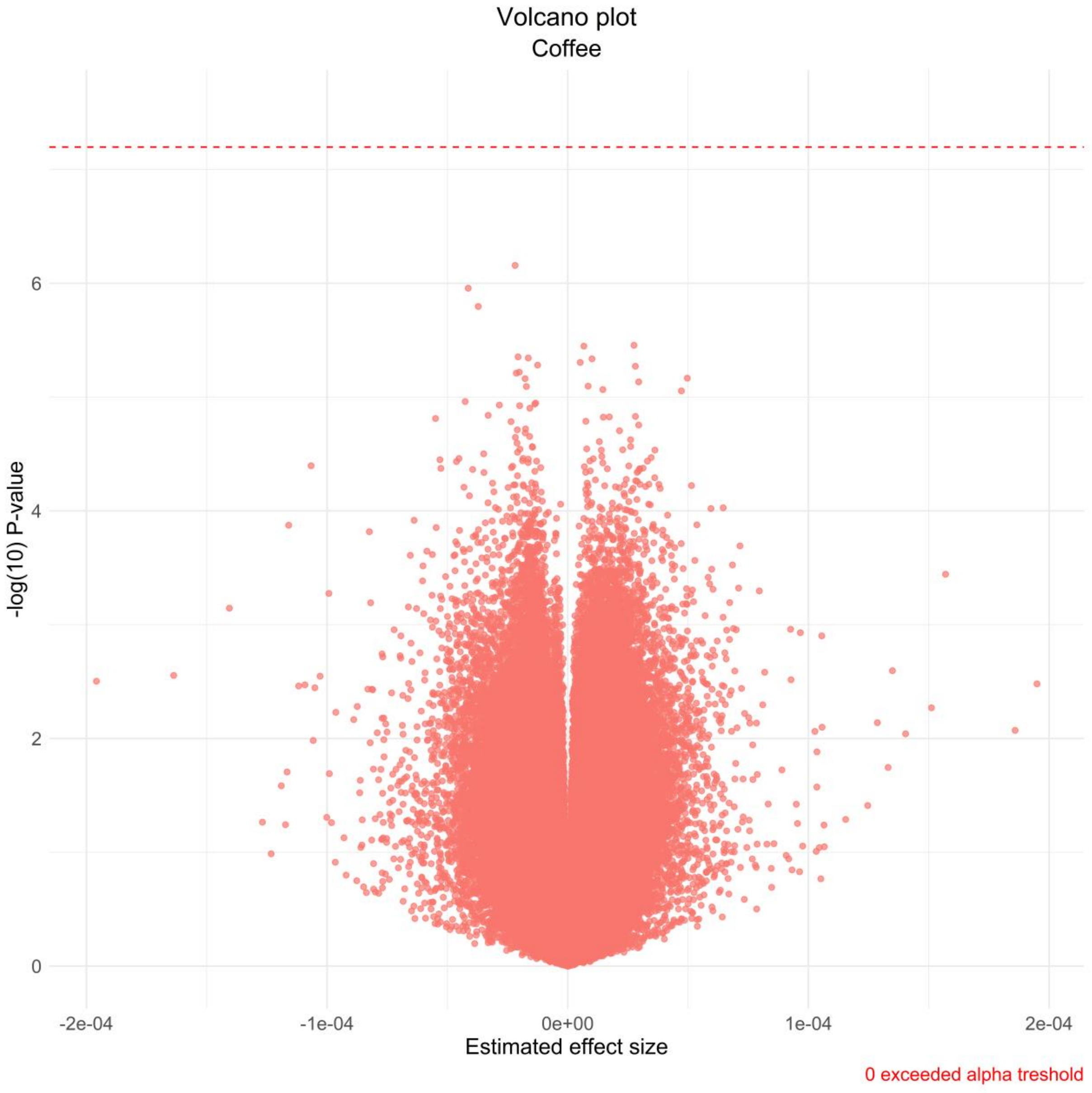

Volcano plot
Coffee
-log(10) P-value
6
4
2
0
-2e-04
-1e-04
0e+00
1e-04
2e-04
Estimated effect size
0 exceeded alpha treshold

Figure S9

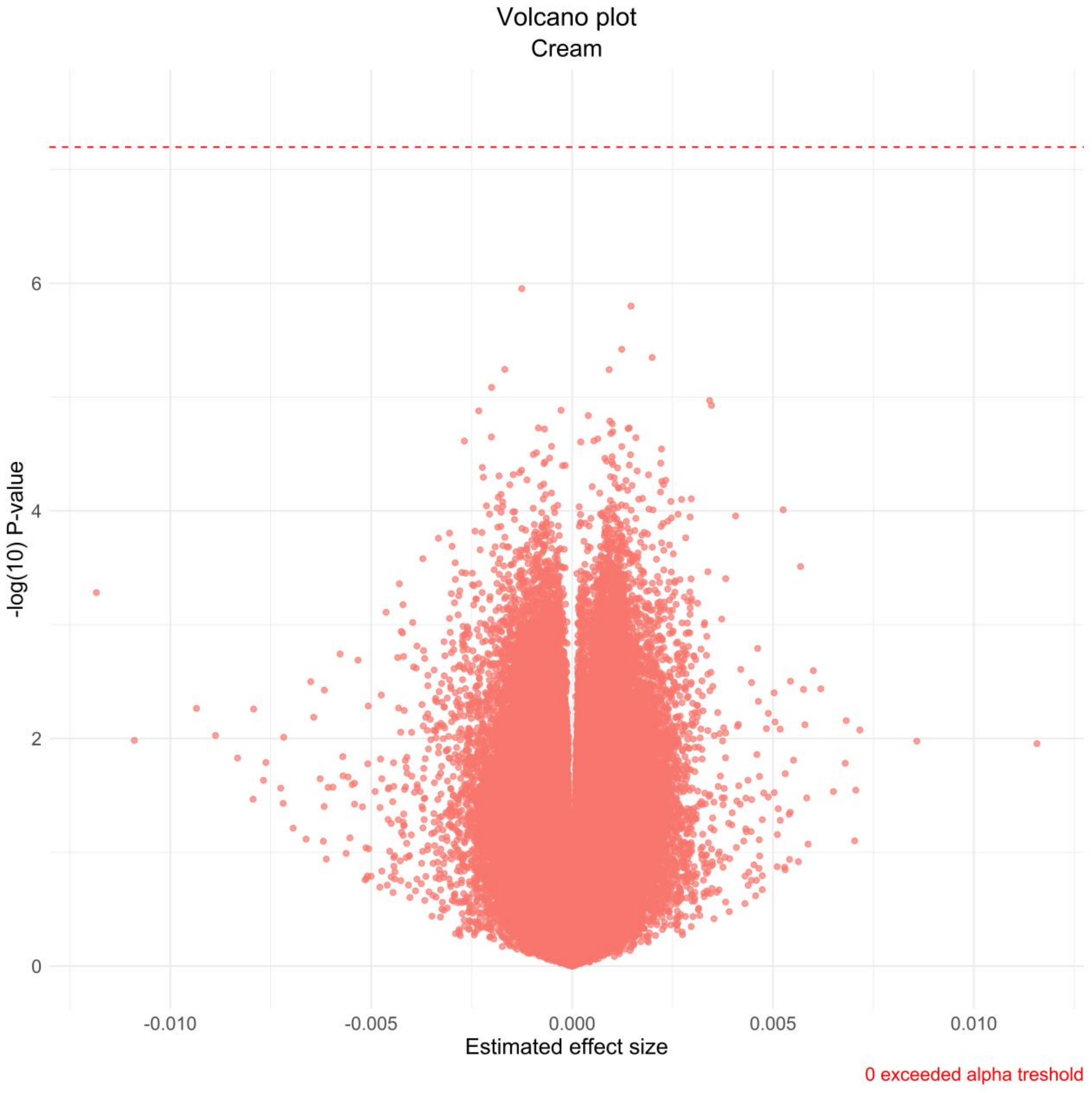

Volcano plot
Cream
-log(10) P-value
0
2
4
6
-0.010
-0.005
0.000
0.005
0.010
Estimated effect size
0 exceeded alpha treshold

Figure S10

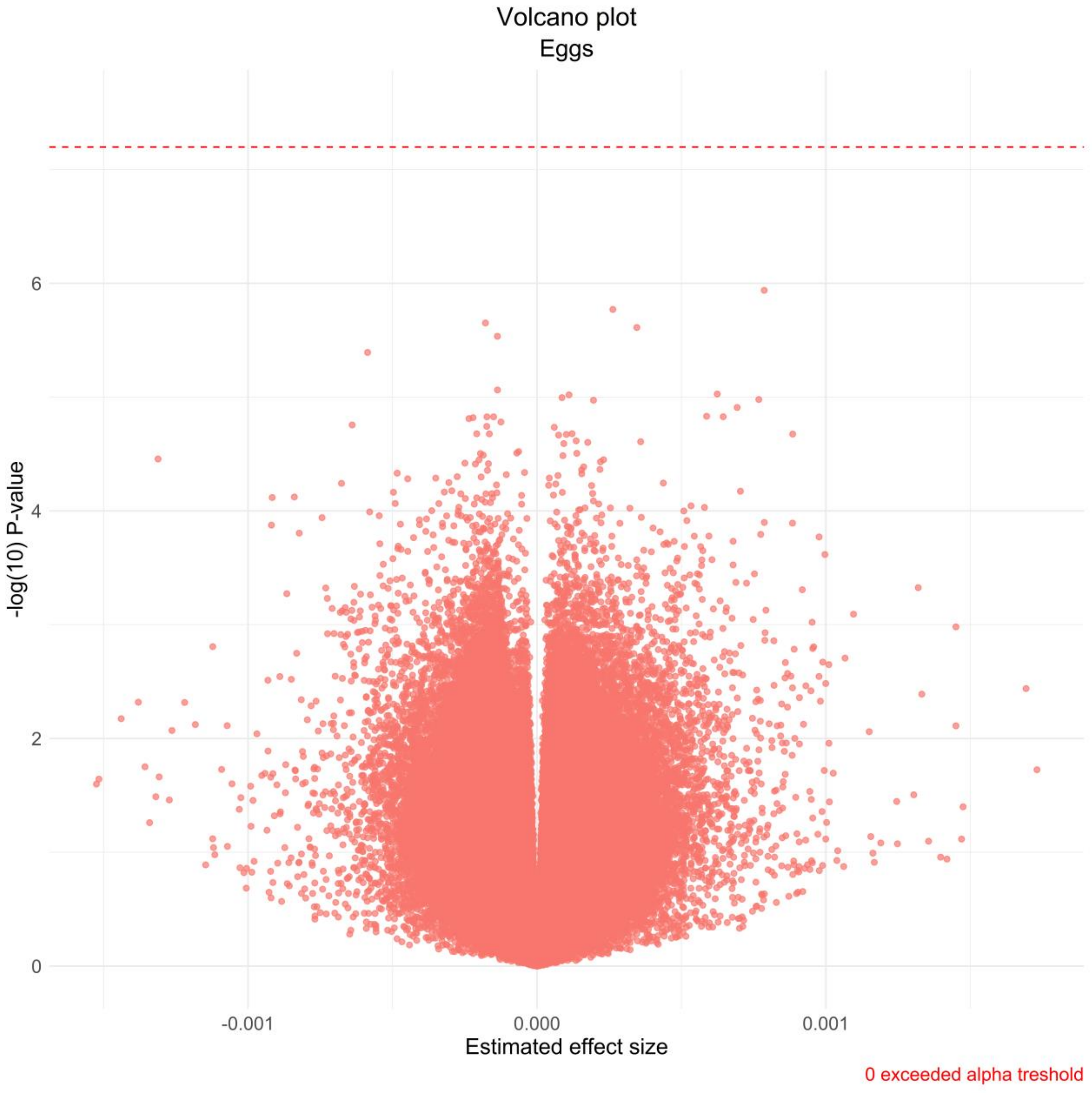
Volcano plot
Eggs
6
4
2
0
-log(10) P-value
-0.001
0.000
0.001
Estimated effect size
0 exceeded alpha treshold

Figure S11

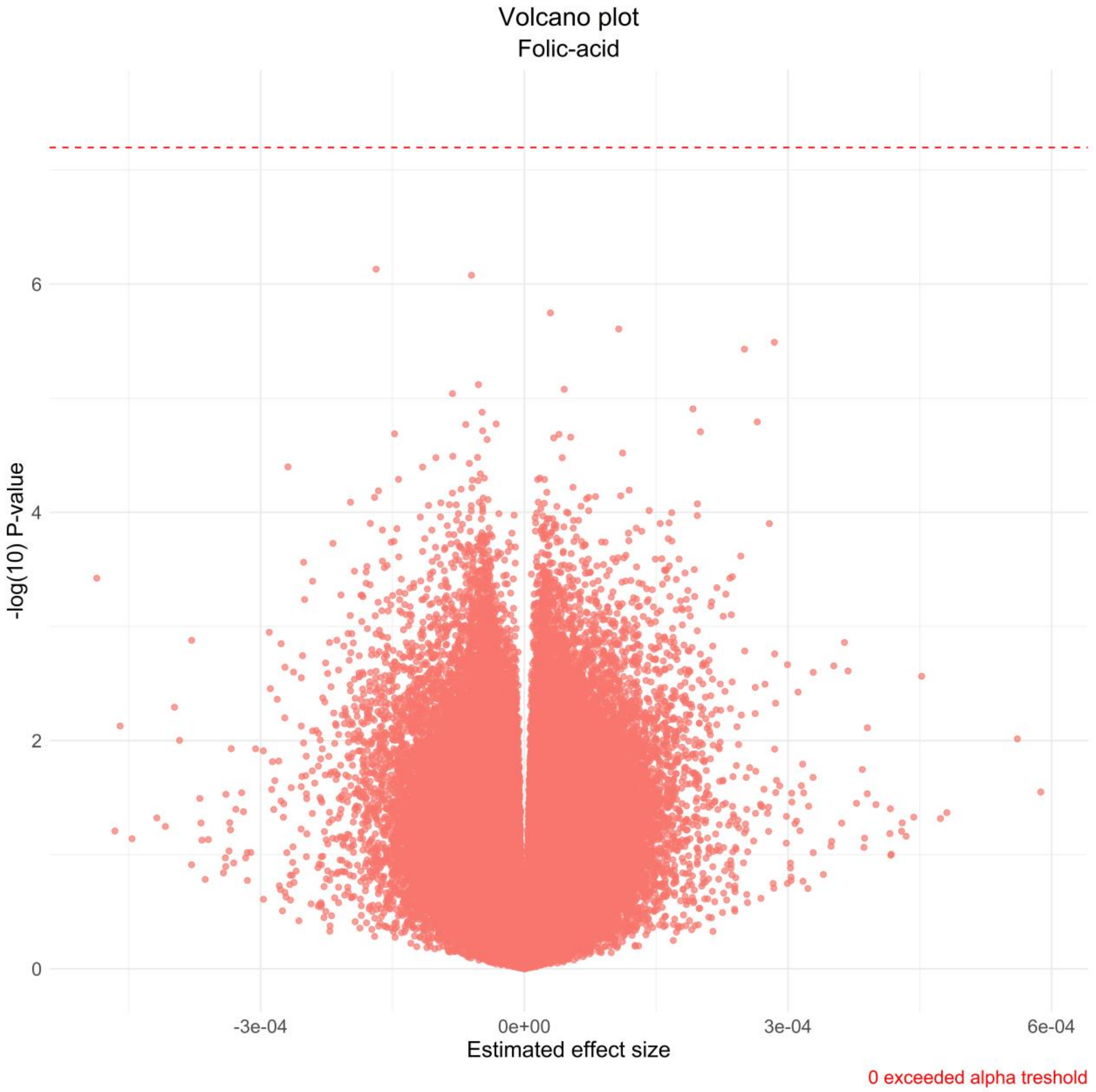
Volcano plot
Folic-acid
6
4
2
0
-log(10) P-value
-3e-04
0e+00
3e-04
6e-04
Estimated effect size
0 exceeded alpha treshold

Figure S12

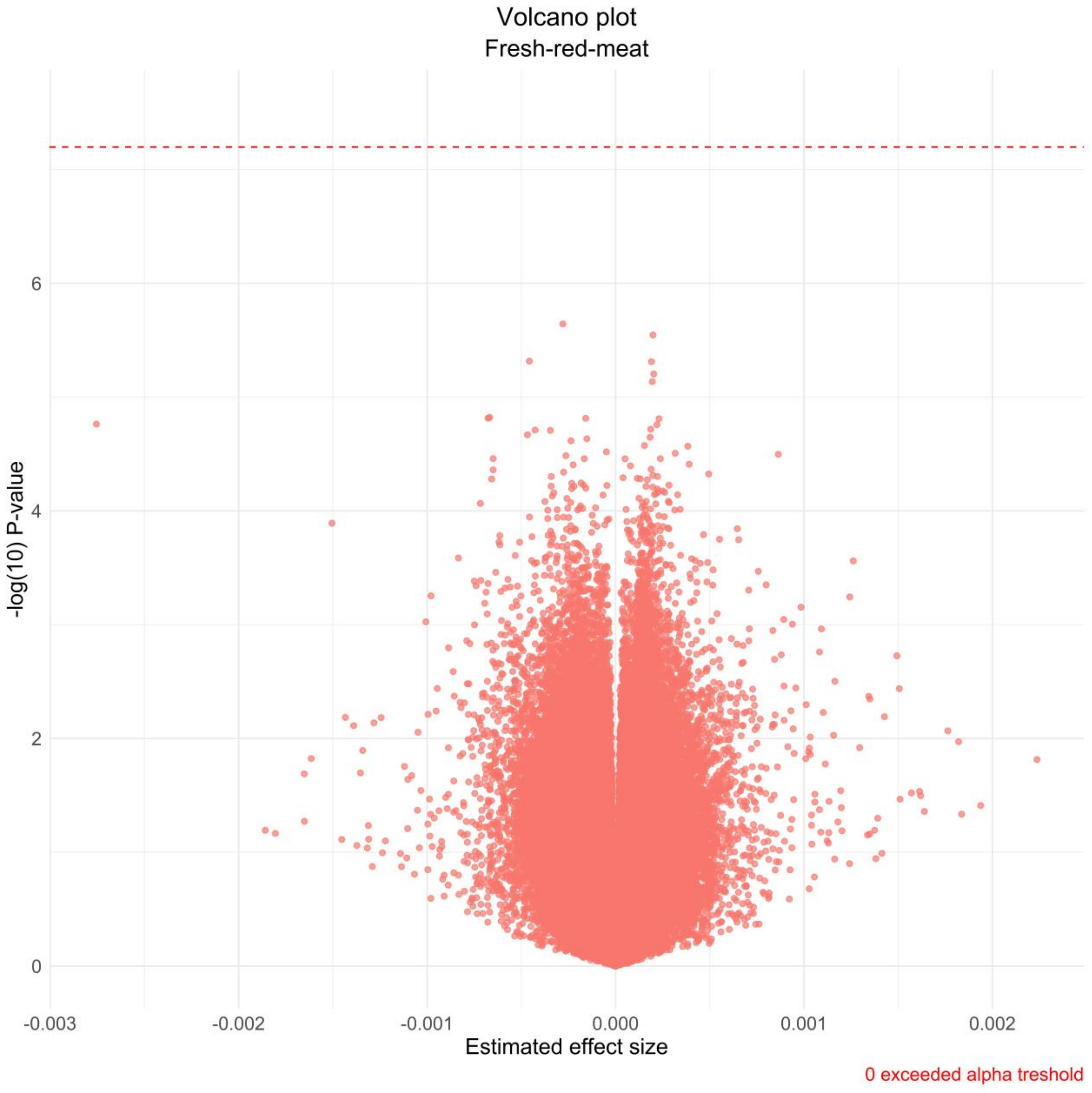
Volcano plot
Fresh-red-meat
-log(10) P-value
6
4
2
0
-0.003
-0.002
-0.001
0.000
0.001
0.002
Estimated effect size
0 exceeded alpha treshold

Figure S13

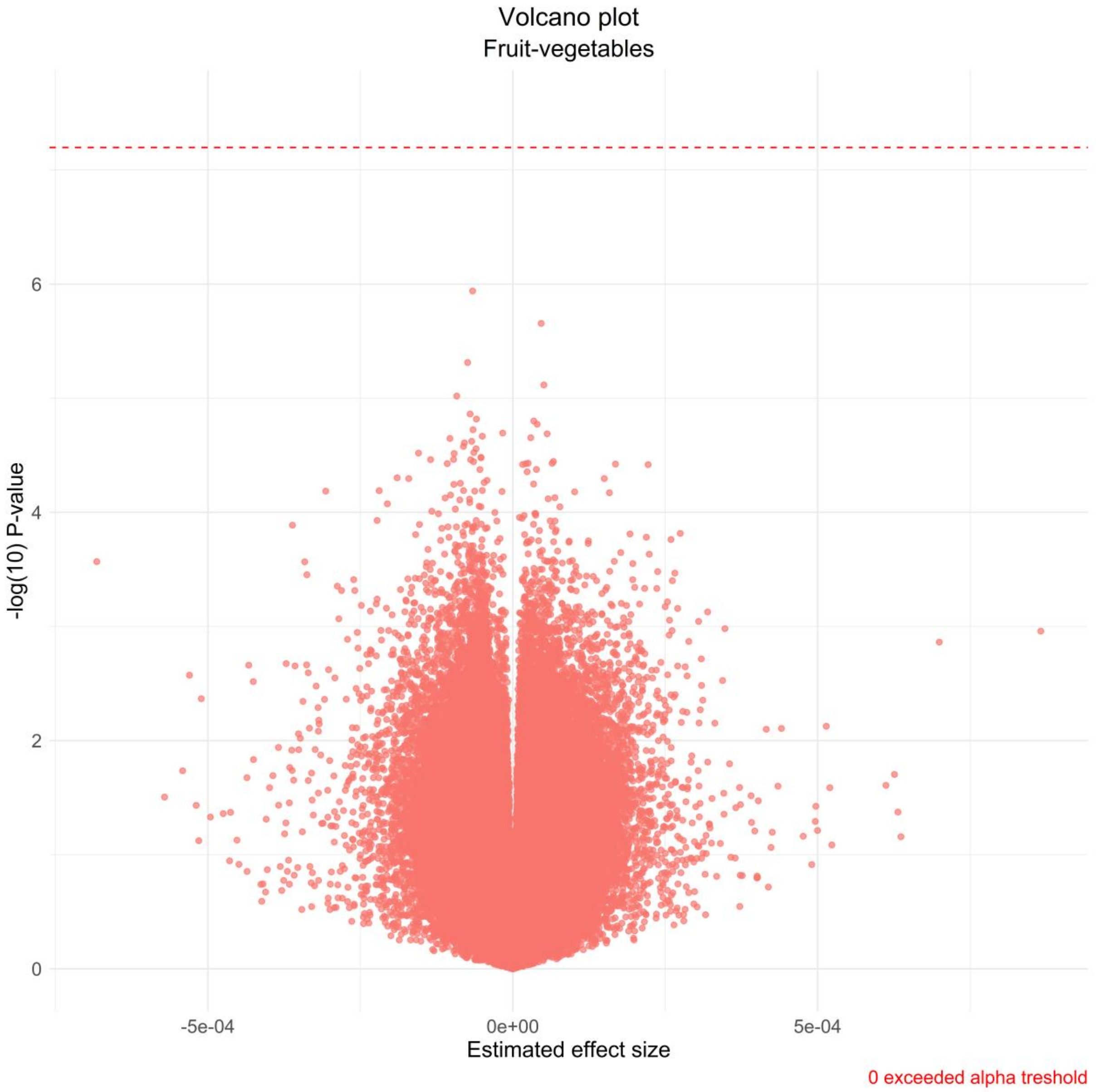
Volcano plot
Fruit-vegetables
-log(10) P-value
6
4
2
0
-5e-04
0e+00
5e-04
Estimated effect size
0 exceeded alpha treshold

Figure S14

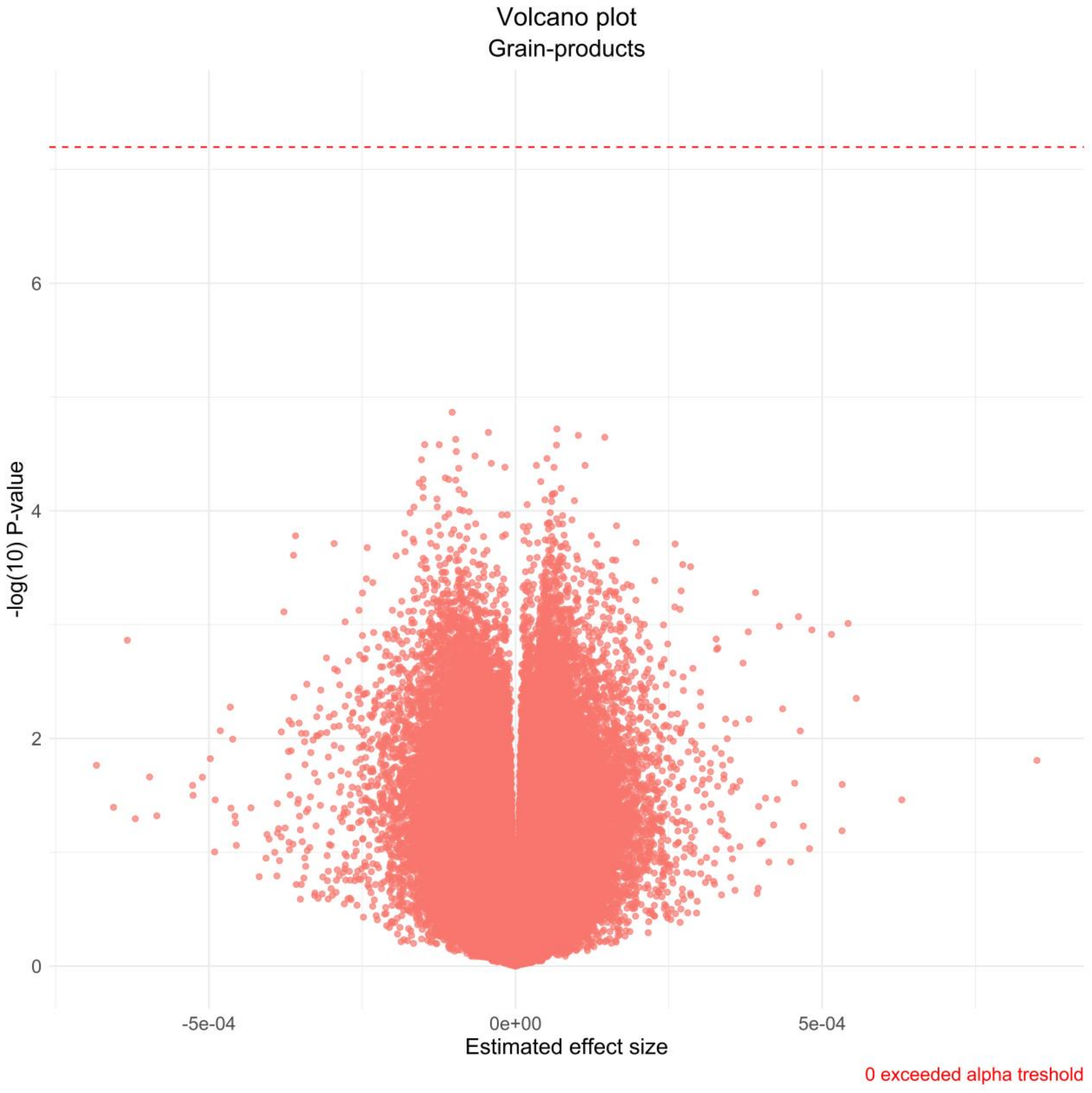

Volcano plot
Grain-products
-log(10) P-value
6
4
2
0
-5e-04
0e+00
5e-04
Estimated effect size
0 exceeded alpha treshold

Figure S15

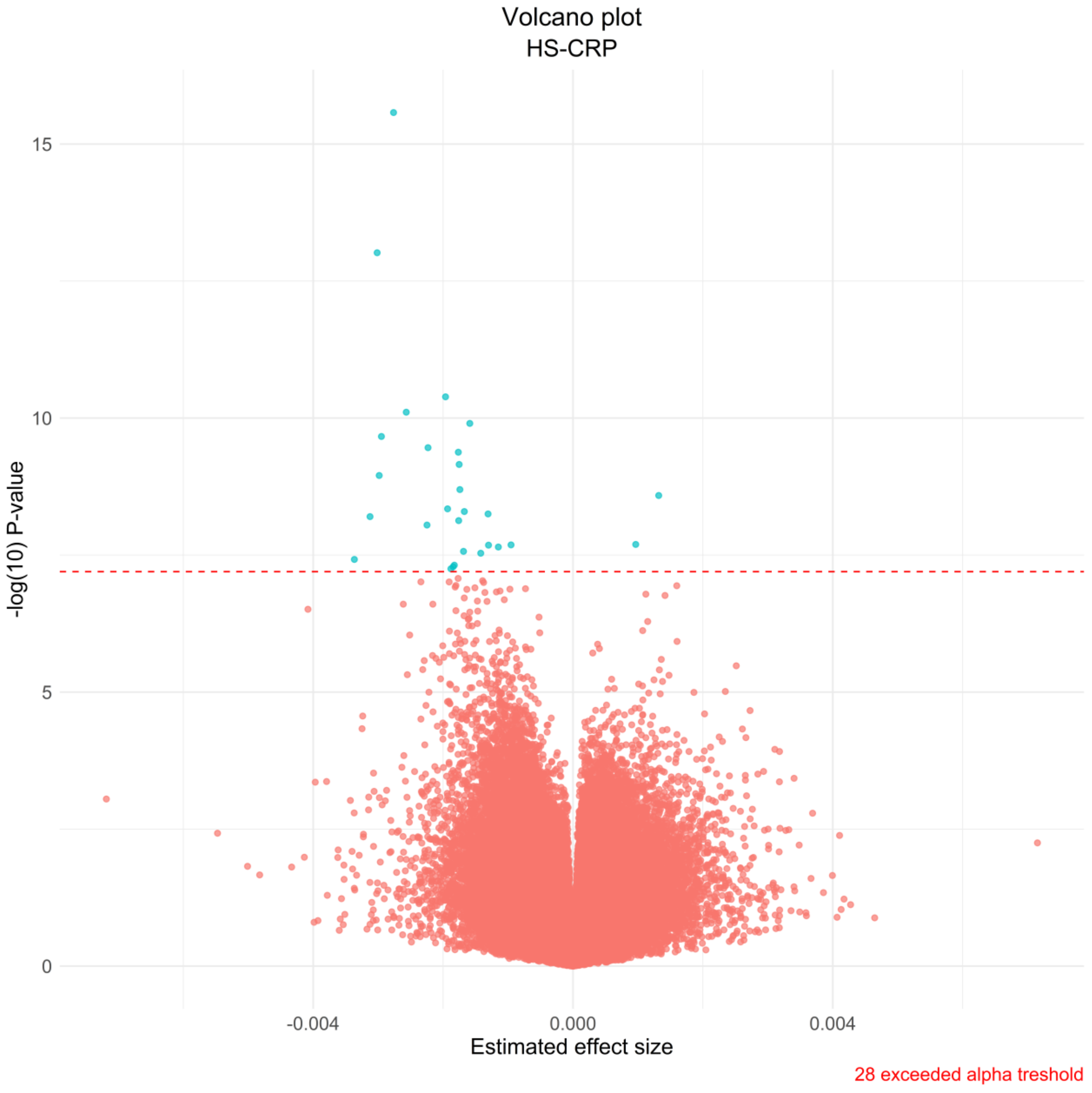

Volcano plot
HS-CRP
15
10
5
0
-log(10) P-value
-0.004
0.000
0.004
Estimated effect size
28 exceeded alpha treshold

Figure S16

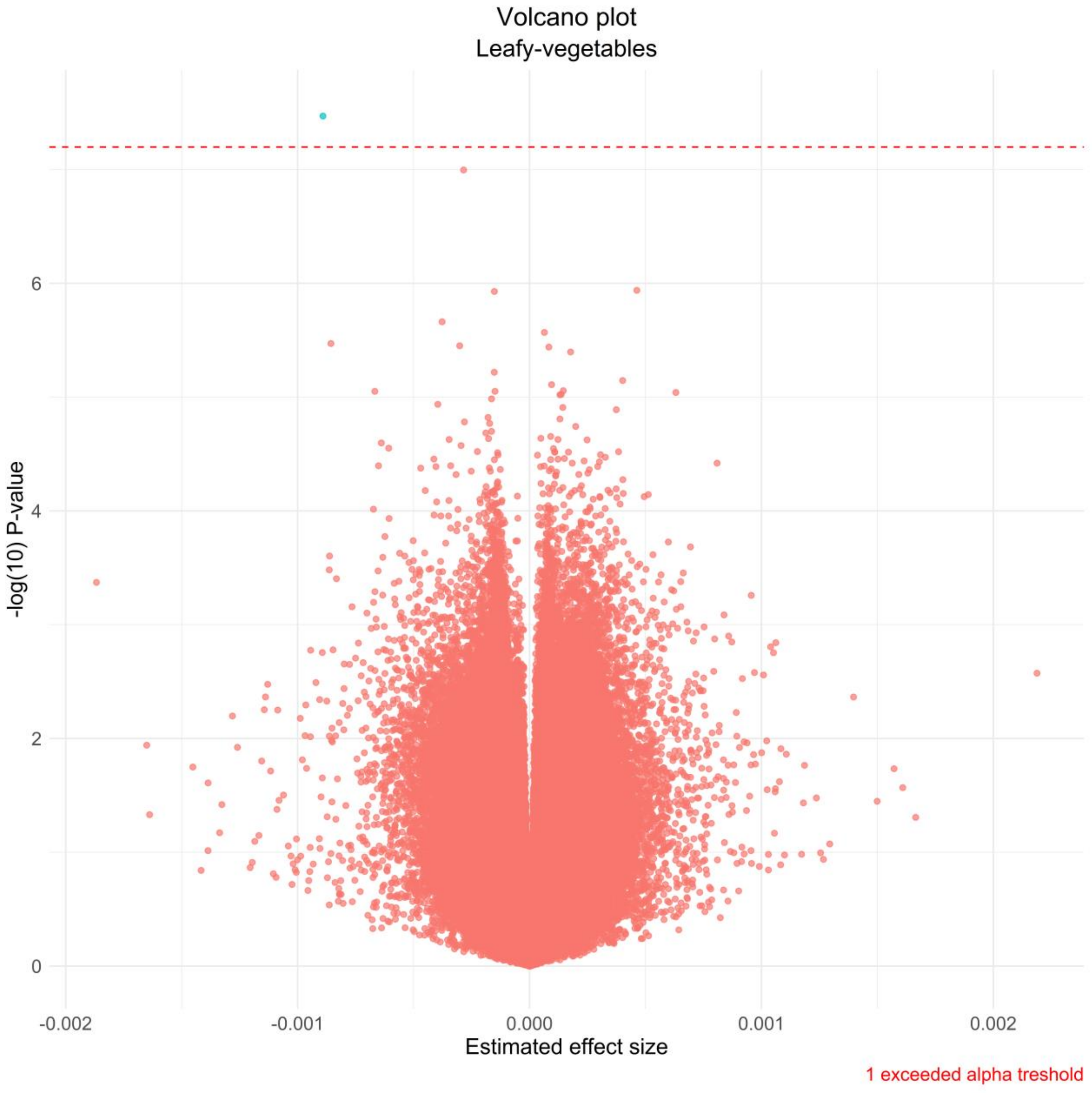

Volcano plot
Leafy-vegetables
-log(10) P-value
0
2
4
6
-0.002
-0.001
0.000
0.001
0.002
Estimated effect size
1 exceeded alpha treshold

Figure S17

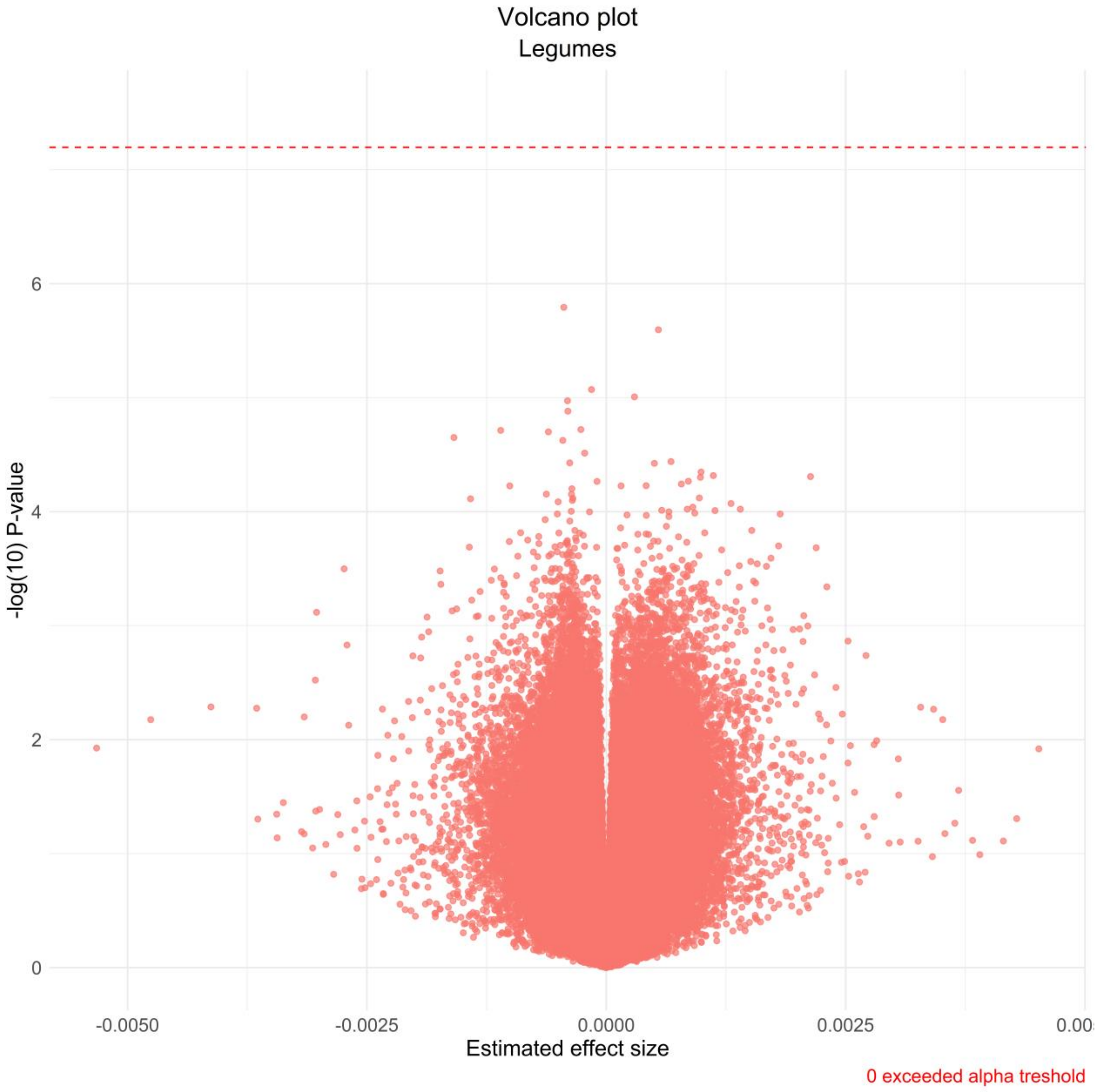
Volcano plot
Legumes
-log(10) P-value
6
4
2
0
-0.0050
-0.0025
0.0000
0.0025
Estimated effect size
0 exceeded alpha treshold

Figure S18

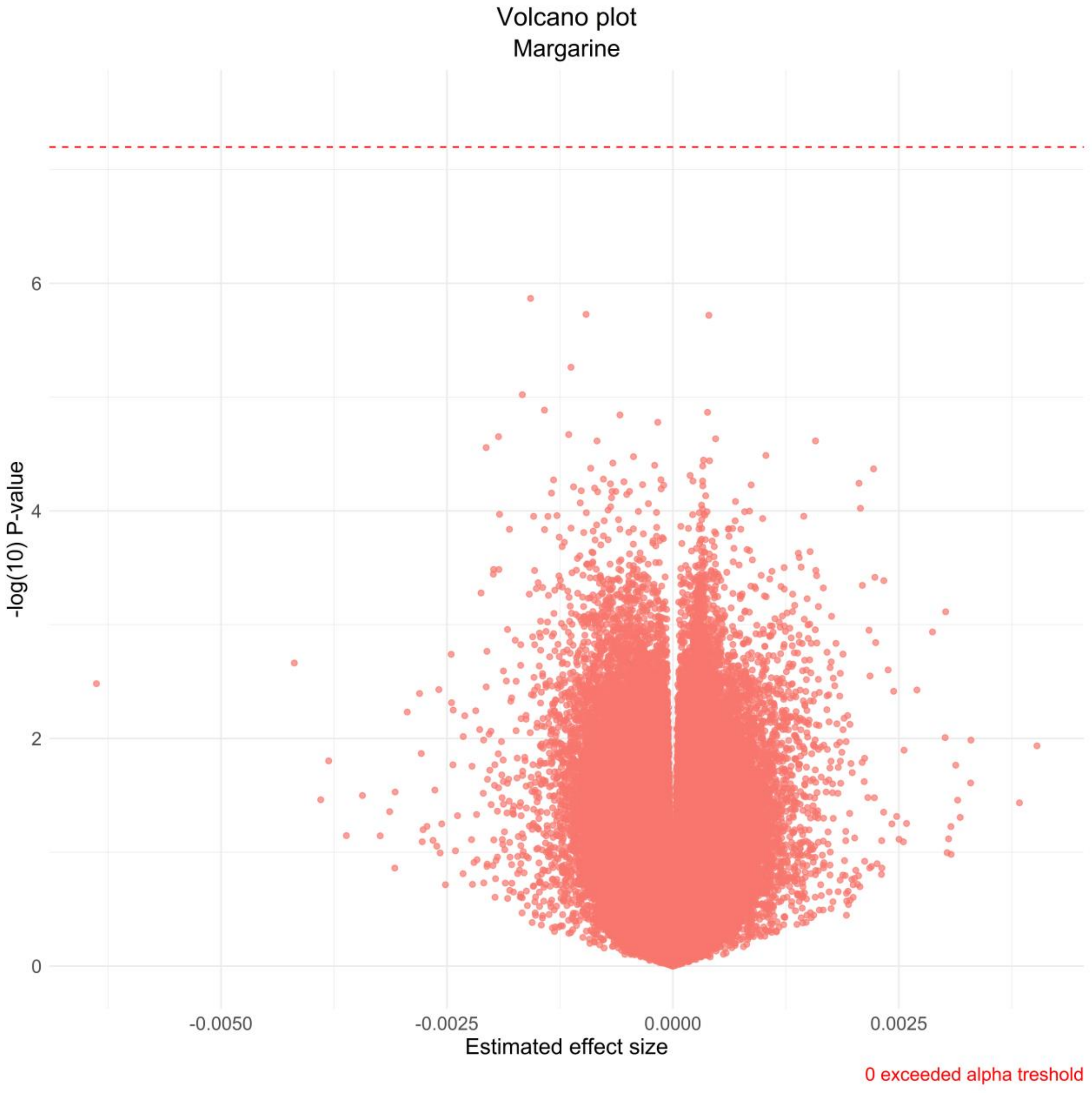
Volcano plot
Margarine
6
4
2
0
-log(10) P-value
-0.0050
-0.0025
0.0000
0.0025
Estimated effect size
0 exceeded alpha treshold

Figure S19

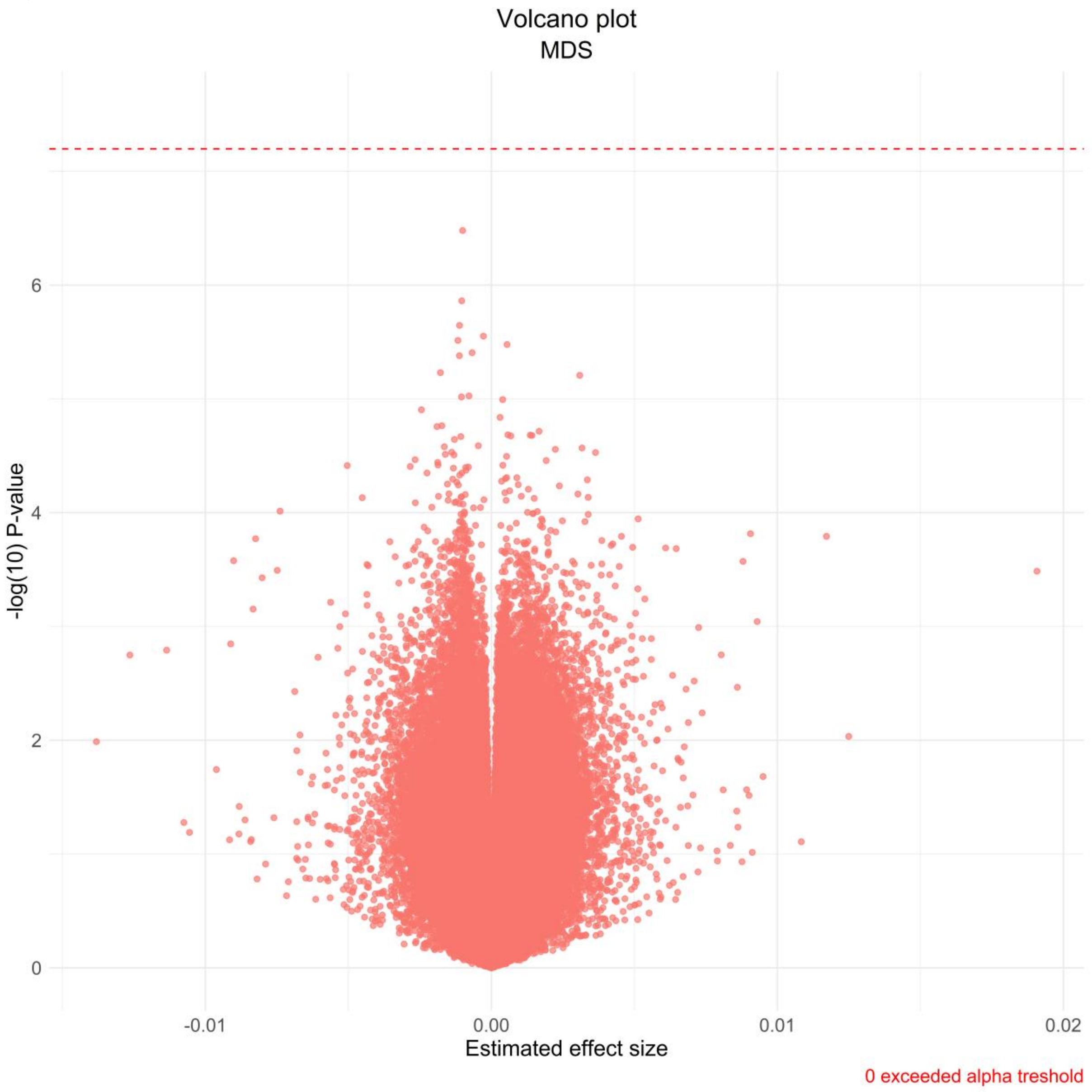
Volcano plot
MDS
-log(10) P-value
0
2
4
6
-0.01
0.00
0.01
0.02
Estimated effect size
0 exceeded alpha treshold

Figure S20

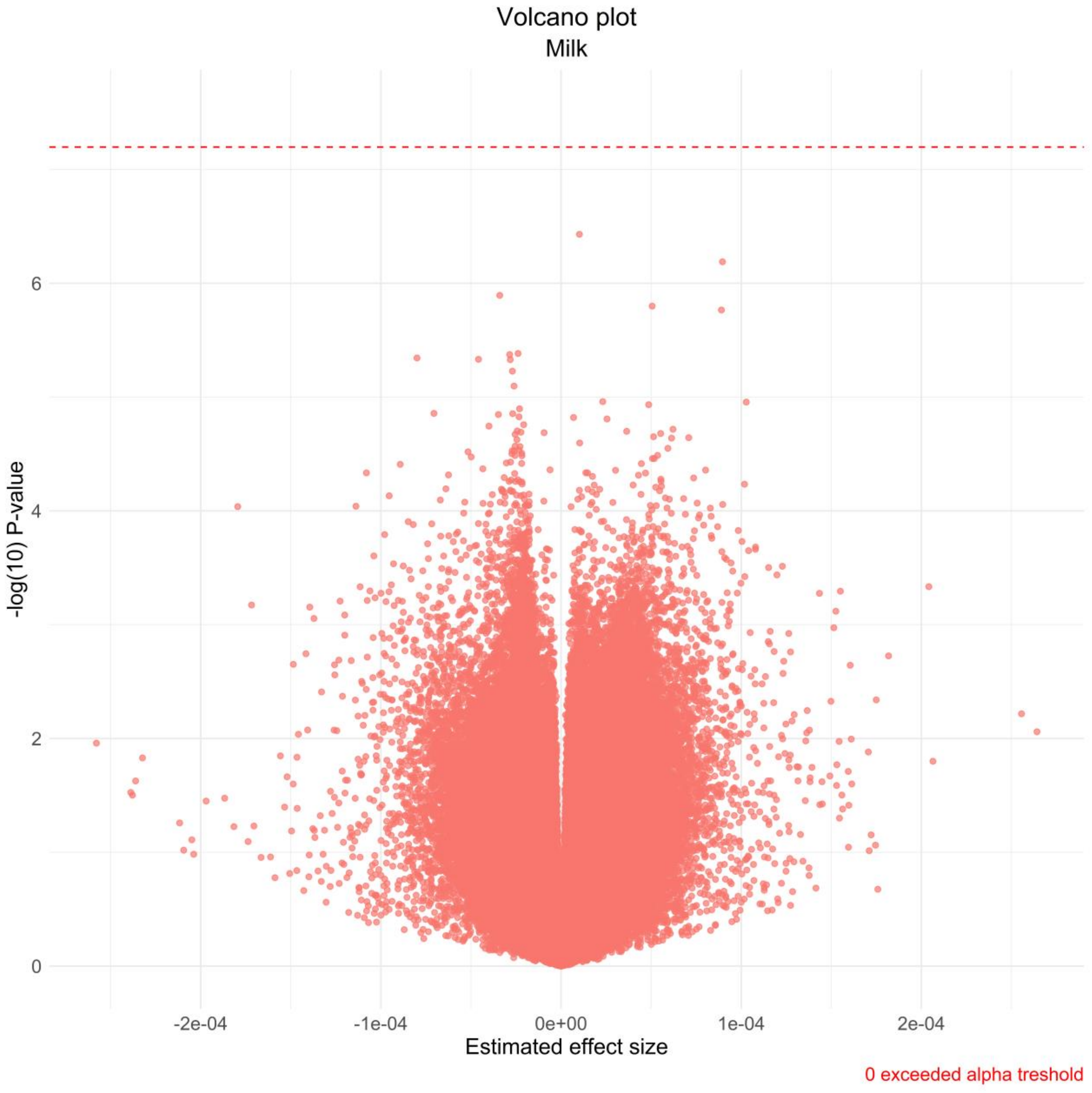
Volcano plot
Milk
-log(10) P-value
0
2
4
6
-2e-04
-1e-04
0e+00
1e-04
2e-04
Estimated effect size
0 exceeded alpha treshold

Figure S21

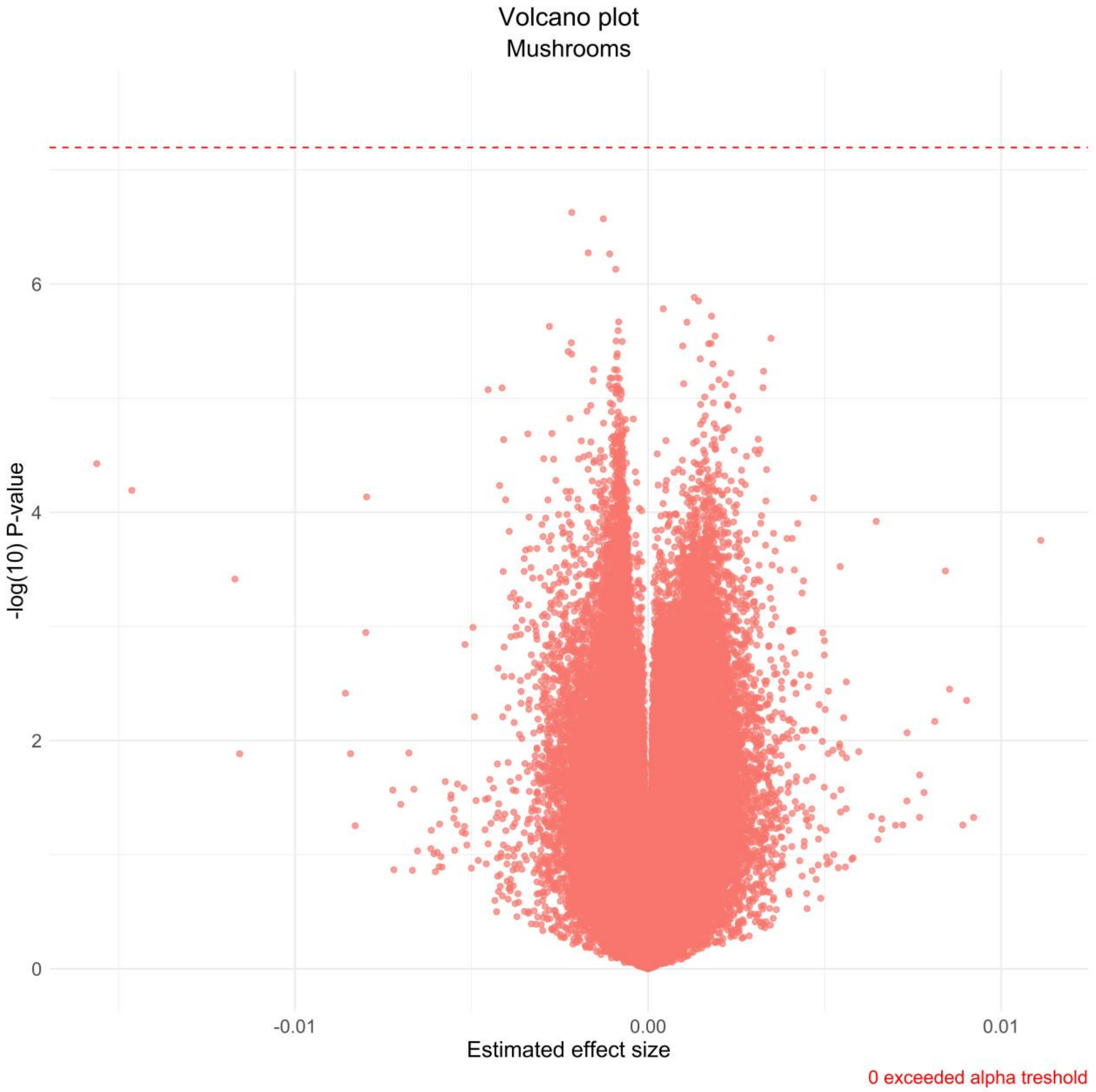
Volcano plot
Mushrooms
-log(10) P-value
0
2
4
6
-0.01
0.00
0.01
Estimated effect size
0 exceeded alpha treshold

Figure S22

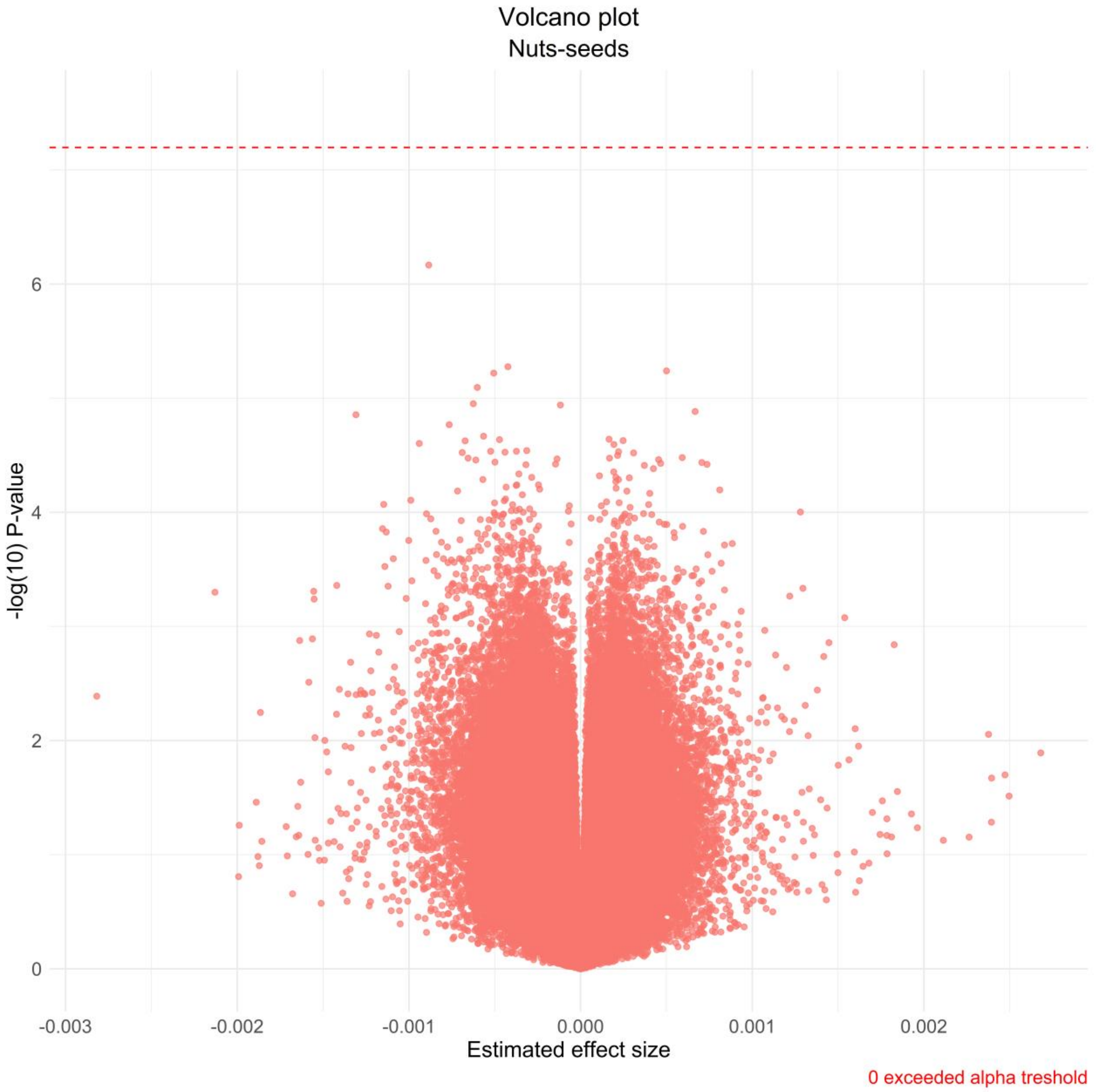
Volcano plot
Nuts-seeds
6
4
2
0
-log(10) P-value
-0.003
-0.002
-0.001
0.000
0.001
0.002
Estimated effect size
0 exceeded alpha treshold

Figure S23

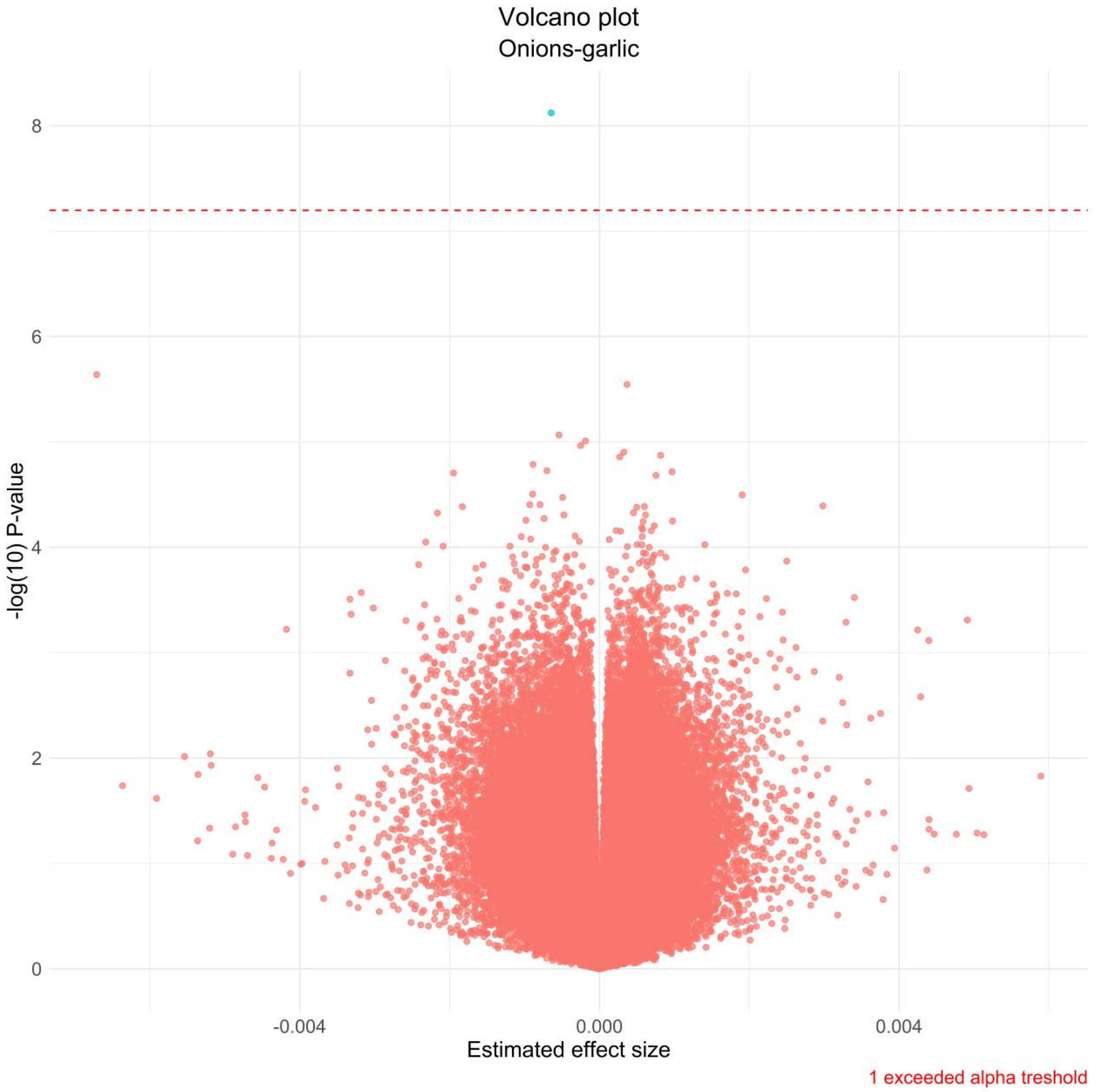
Volcano plot
Onions-garlic
8
6
4
2
0
-log(10) P-value
-0.004
0.000
0.004
Estimated effect size
1 exceeded alpha treshold

Figure S24

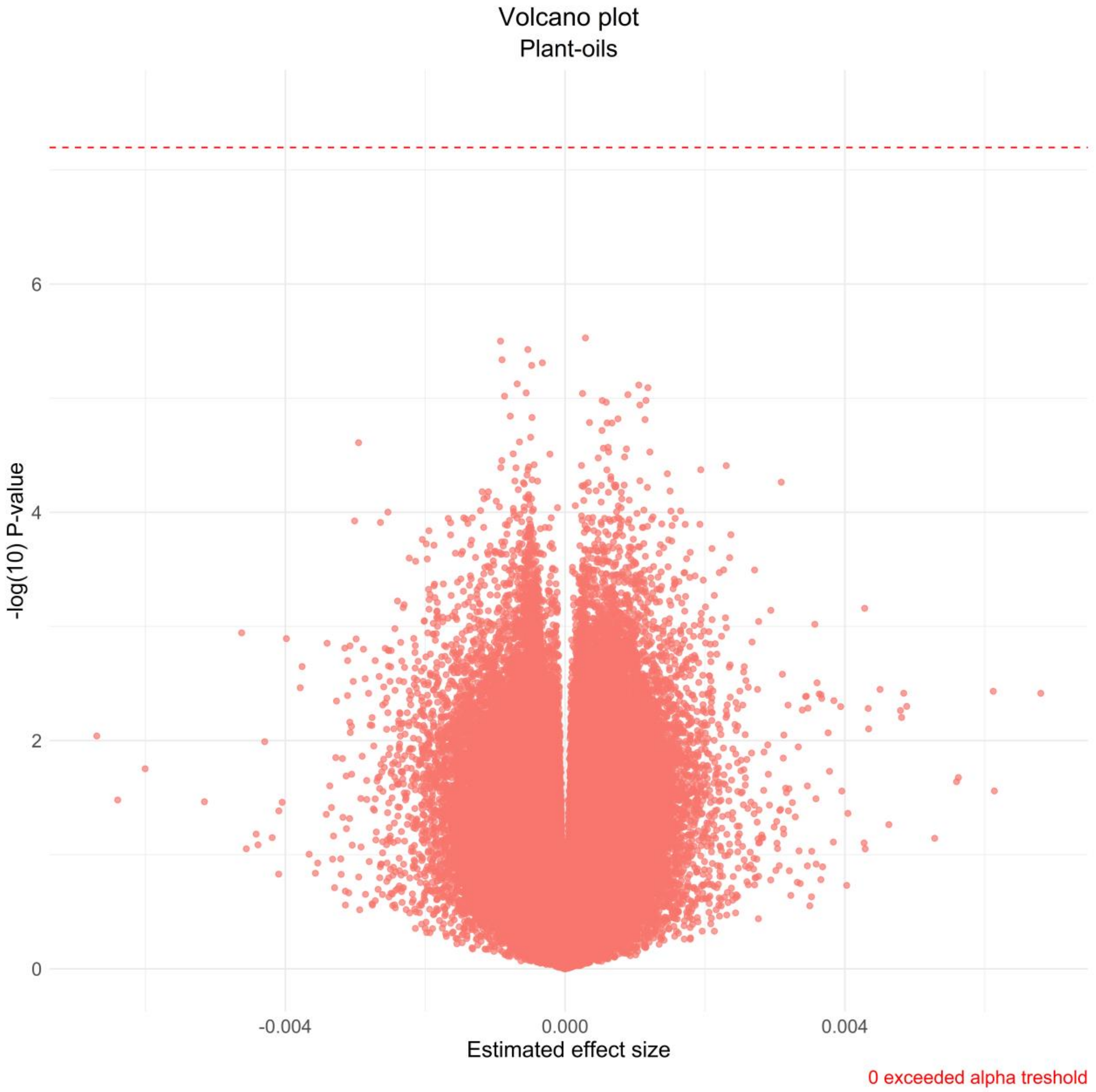

Volcano plot
Plant-oils
6
4
2
0
-log(10) P-value
-0.004
0.000
0.004
Estimated effect size
0 exceeded alpha treshold

Figure S25

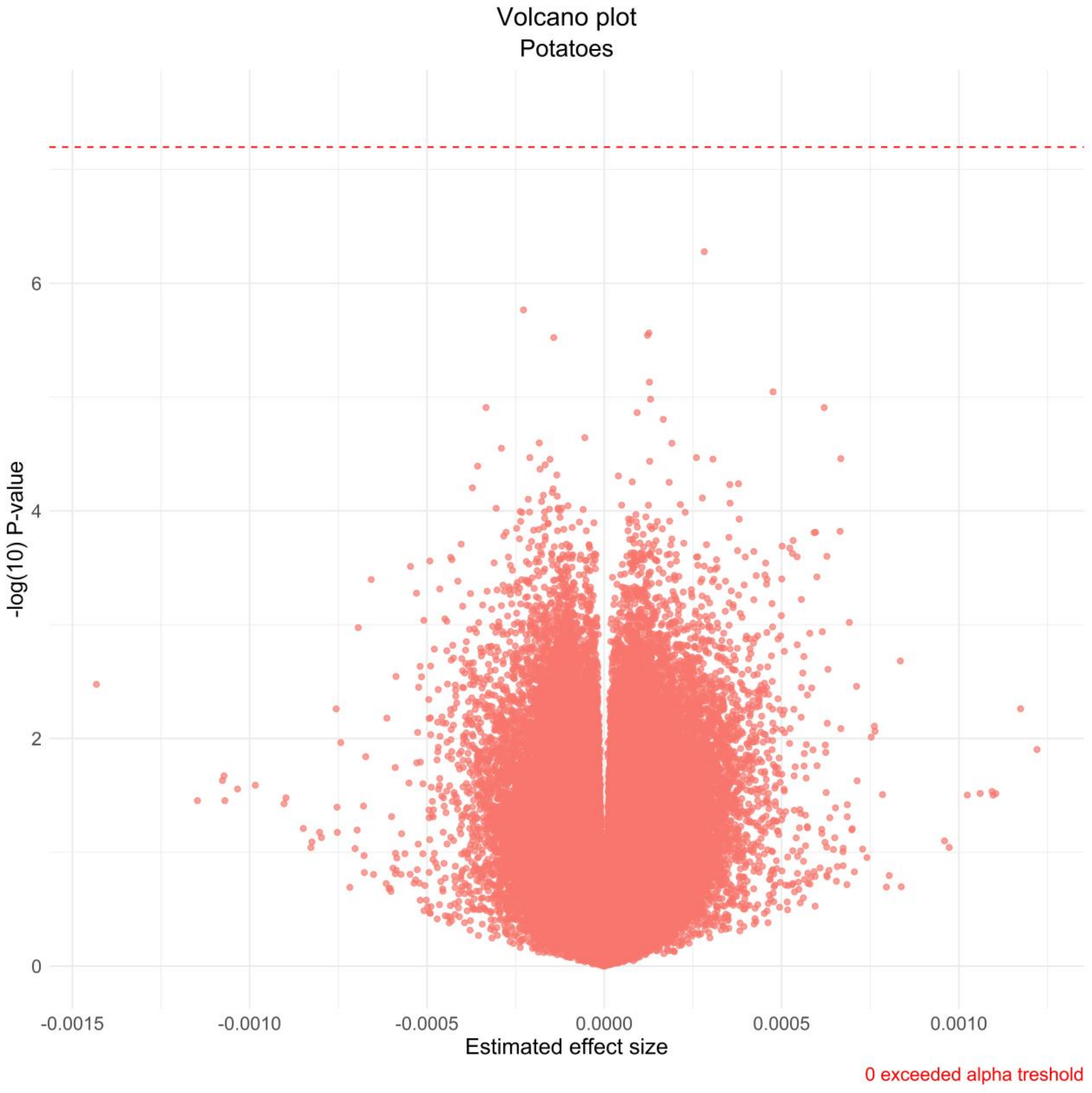
Volcano plot
Potatoes
-log(10) P-value
0
2
4
6
-0.0015
-0.0010
-0.0005
0.0000
0.0005
0.0010
Estimated effect size
0 exceeded alpha treshold

Figure S26

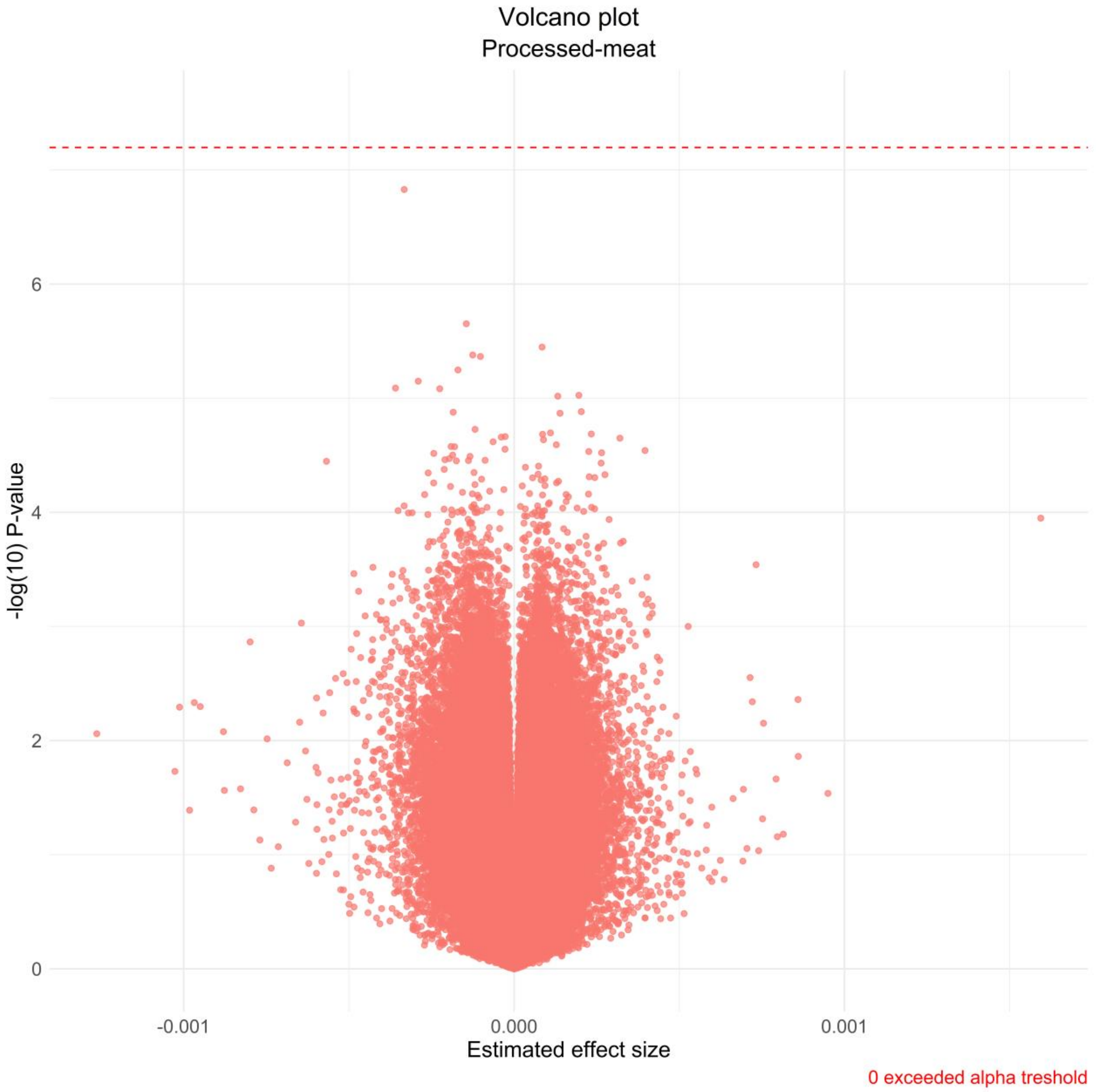

Volcano plot
Processed-meat
-log(10) P-value
6
4
2
0
-0.001
0.000
0.001
Estimated effect size
0 exceeded alpha treshold

Figure S27

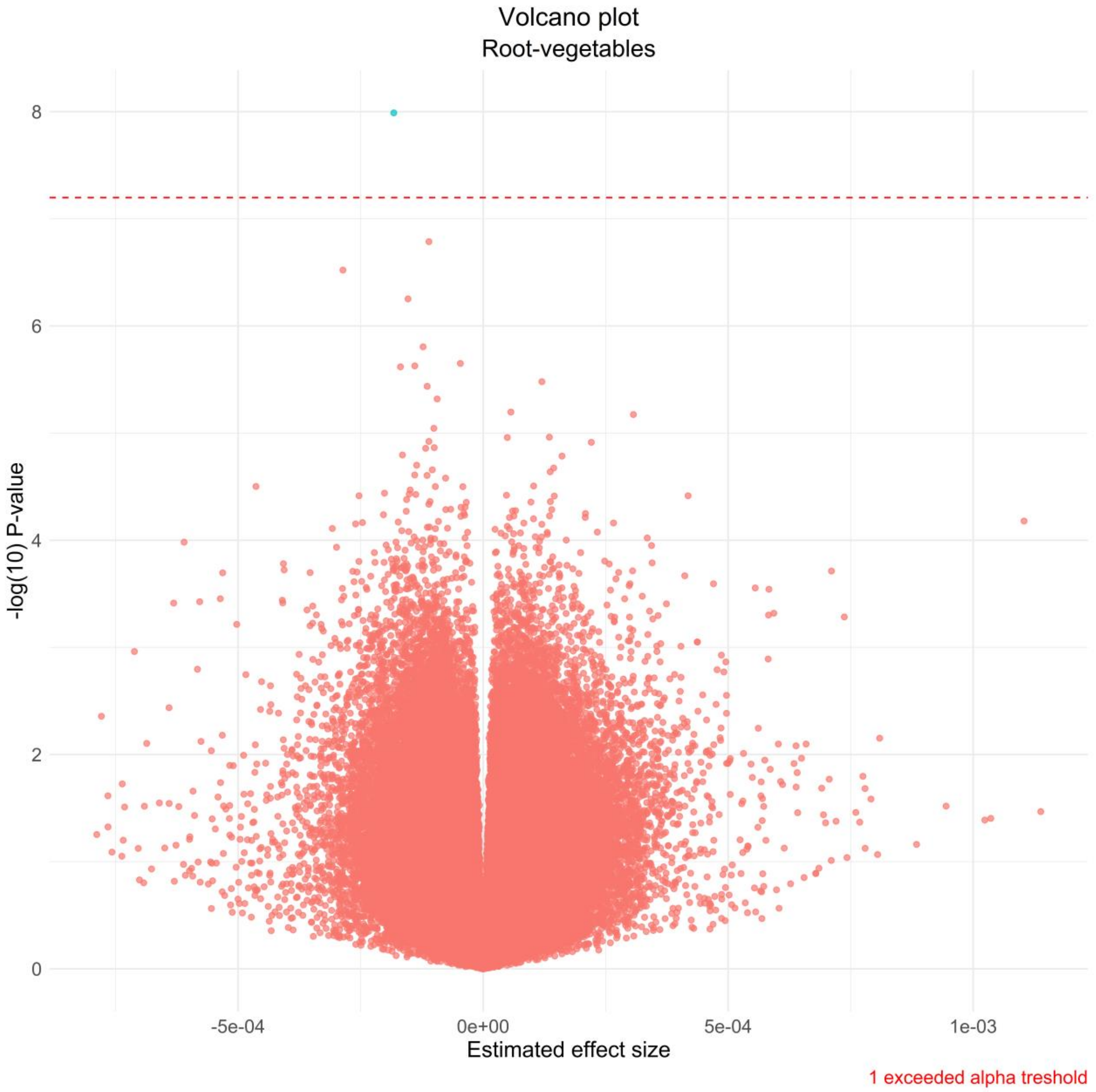

Volcano plot
Root-vegetables
8
6
4
2
0
-log(10) P-value
-5e-04
0e+00
5e-04
1e-03
Estimated effect size
1 exceeded alpha treshold

Figure S28

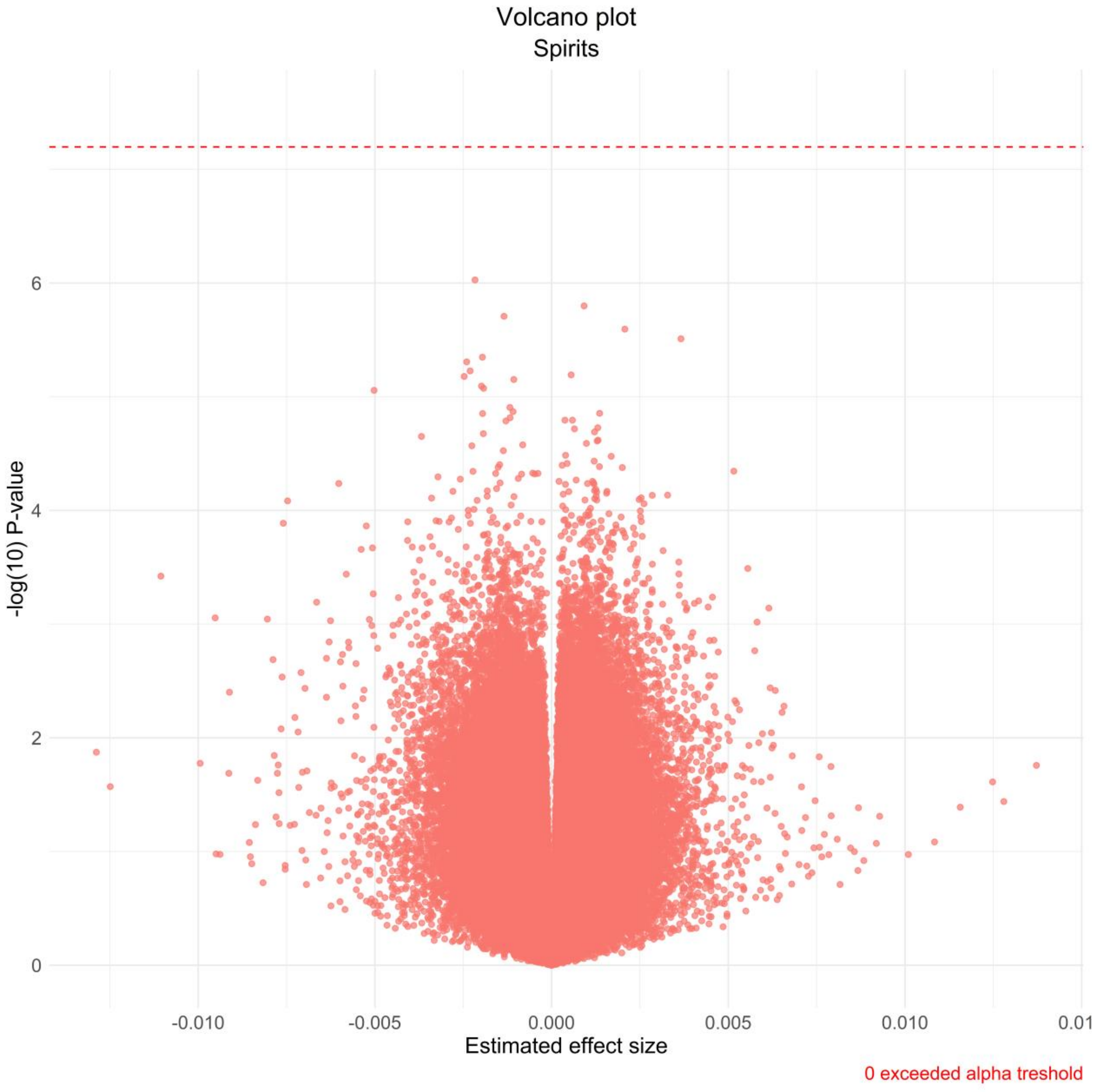

Volcano plot
Spirits
-log(10) P-value
0
2
4
6
-0.010
-0.005
0.000
0.005
0.010
0.01
Estimated effect size
0 exceeded alpha treshold

Figure S29

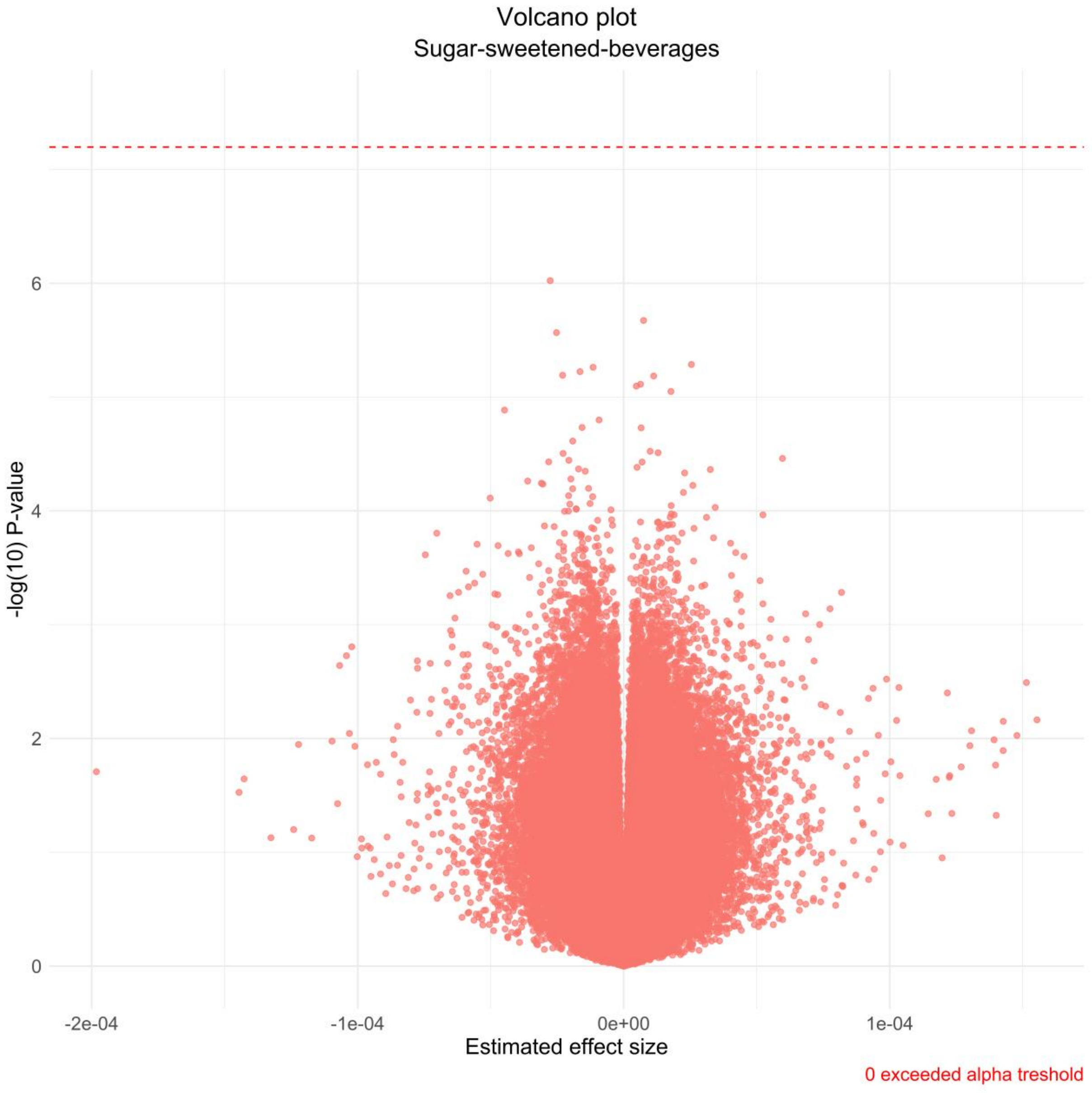

Volcano plot
Sugar-sweetened-beverages
-log(10) P-value
6
4
2
0
-2e-04
-1e-04
0e+00
1e-04
Estimated effect size
0 exceeded alpha treshold

Figure S30

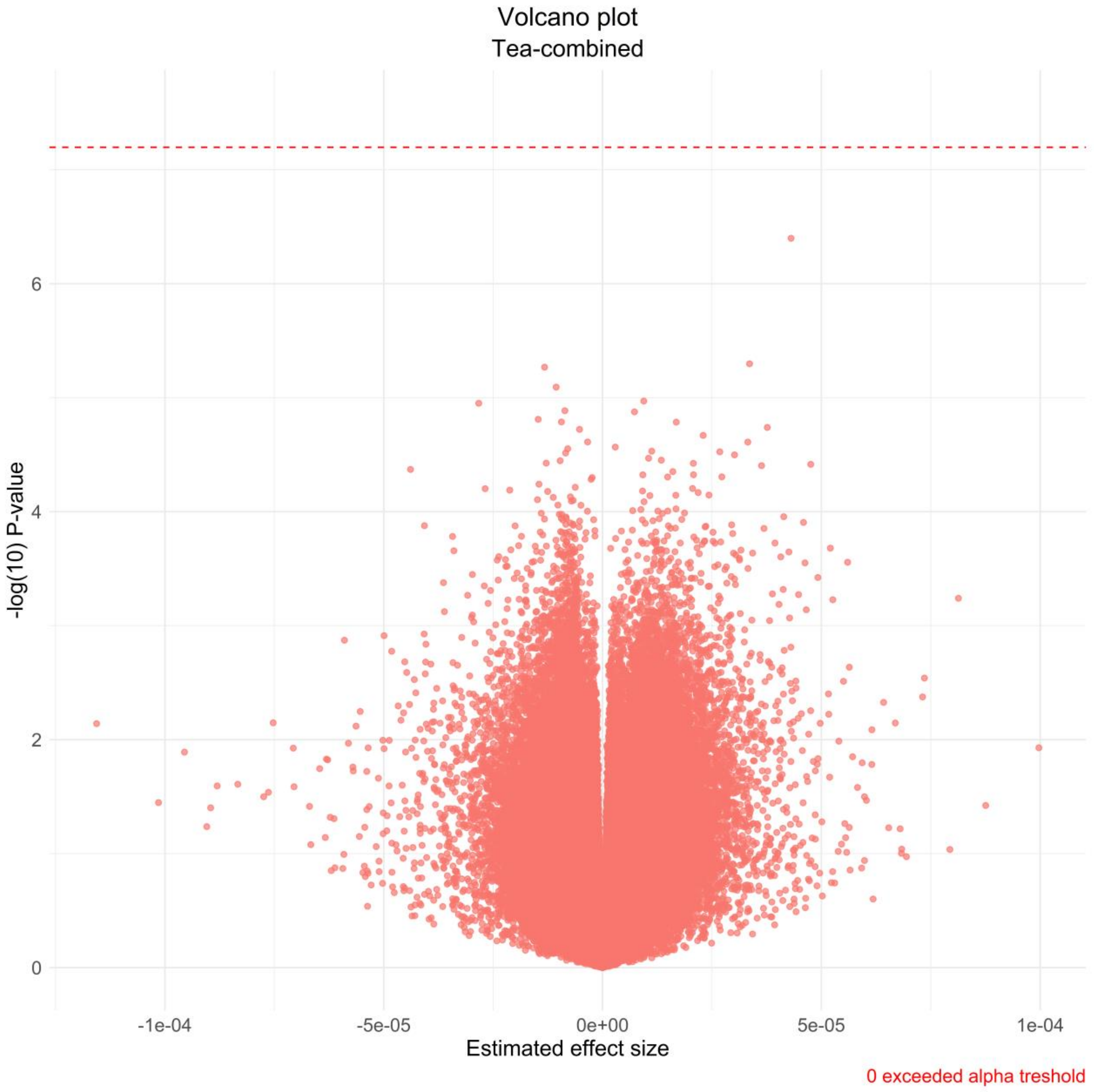

Volcano plot
Tea-combined
-log(10) P-value
0
2
4
6
-1e-04
-5e-05
0e+00
5e-05
1e-04
Estimated effect size
0 exceeded alpha treshold

Figure S31

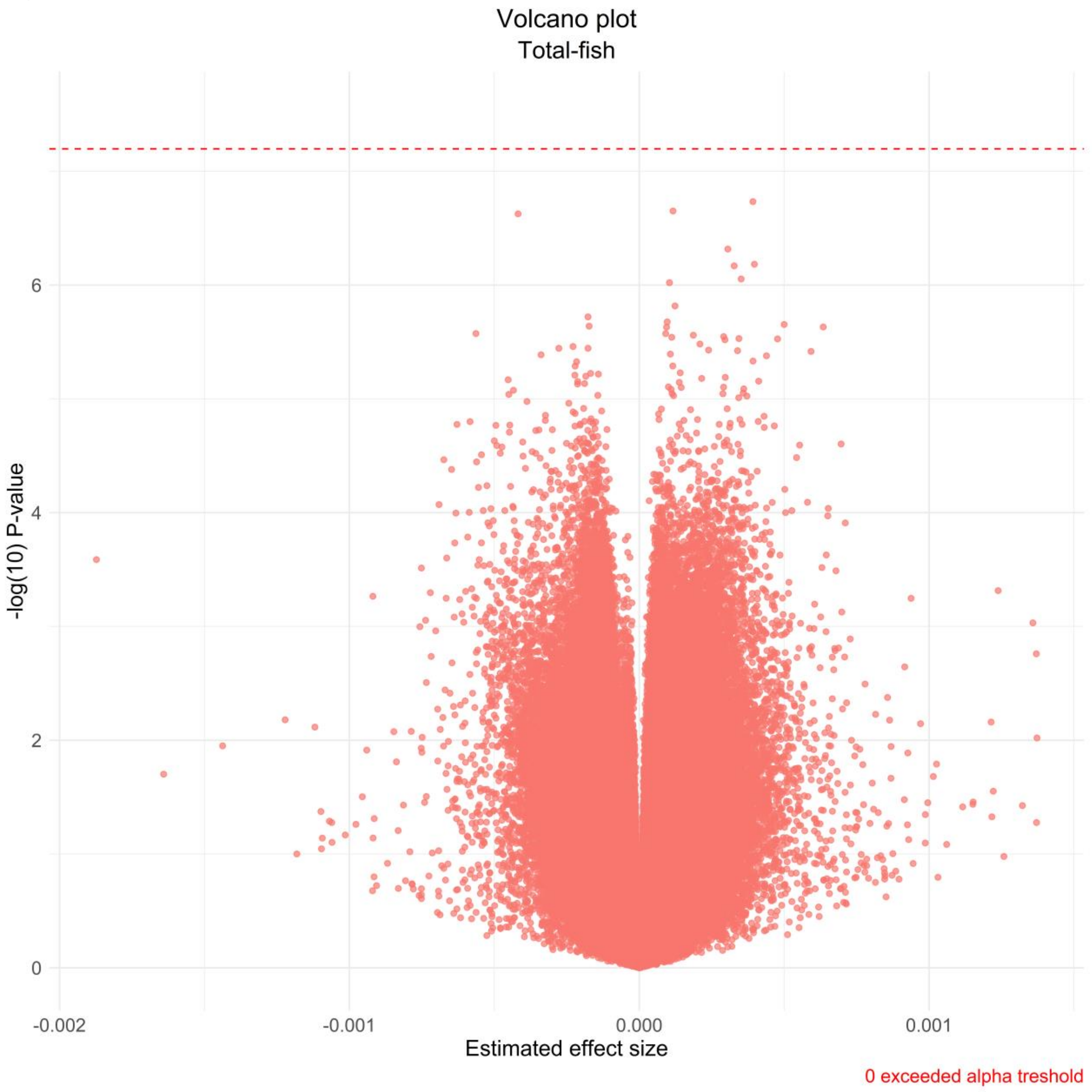
Volcano plot
Total-fish
-log(10) P-value
0
2
4
6
-0.002
-0.001
0.000
0.001
Estimated effect size
0 exceeded alpha treshold

Figure S32

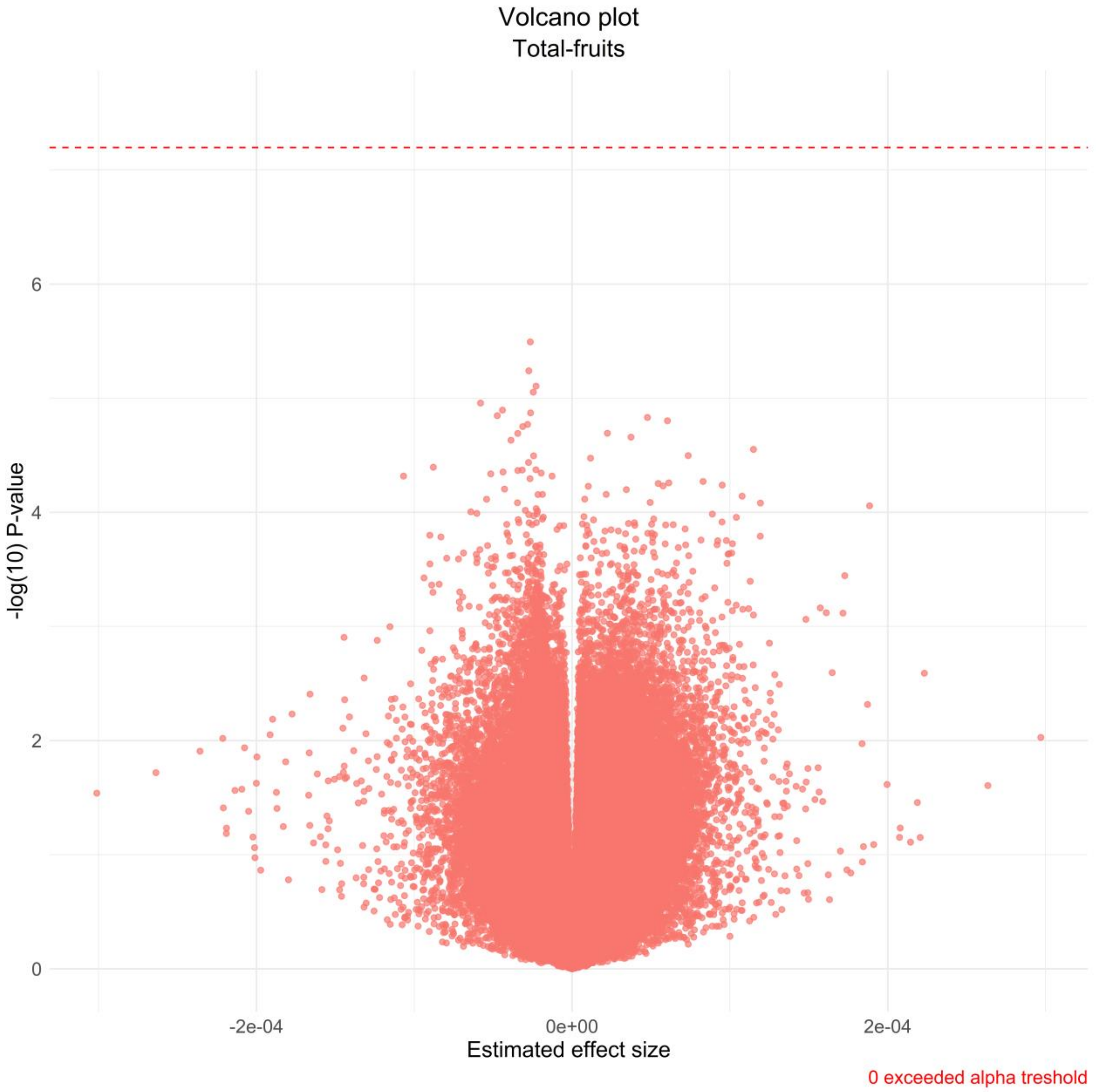
Volcano plot
Total-fruits
6
4
2
0
-log(10) P-value
-2e-04
0e+00
2e-04
Estimated effect size
0 exceeded alpha treshold

Figure S33

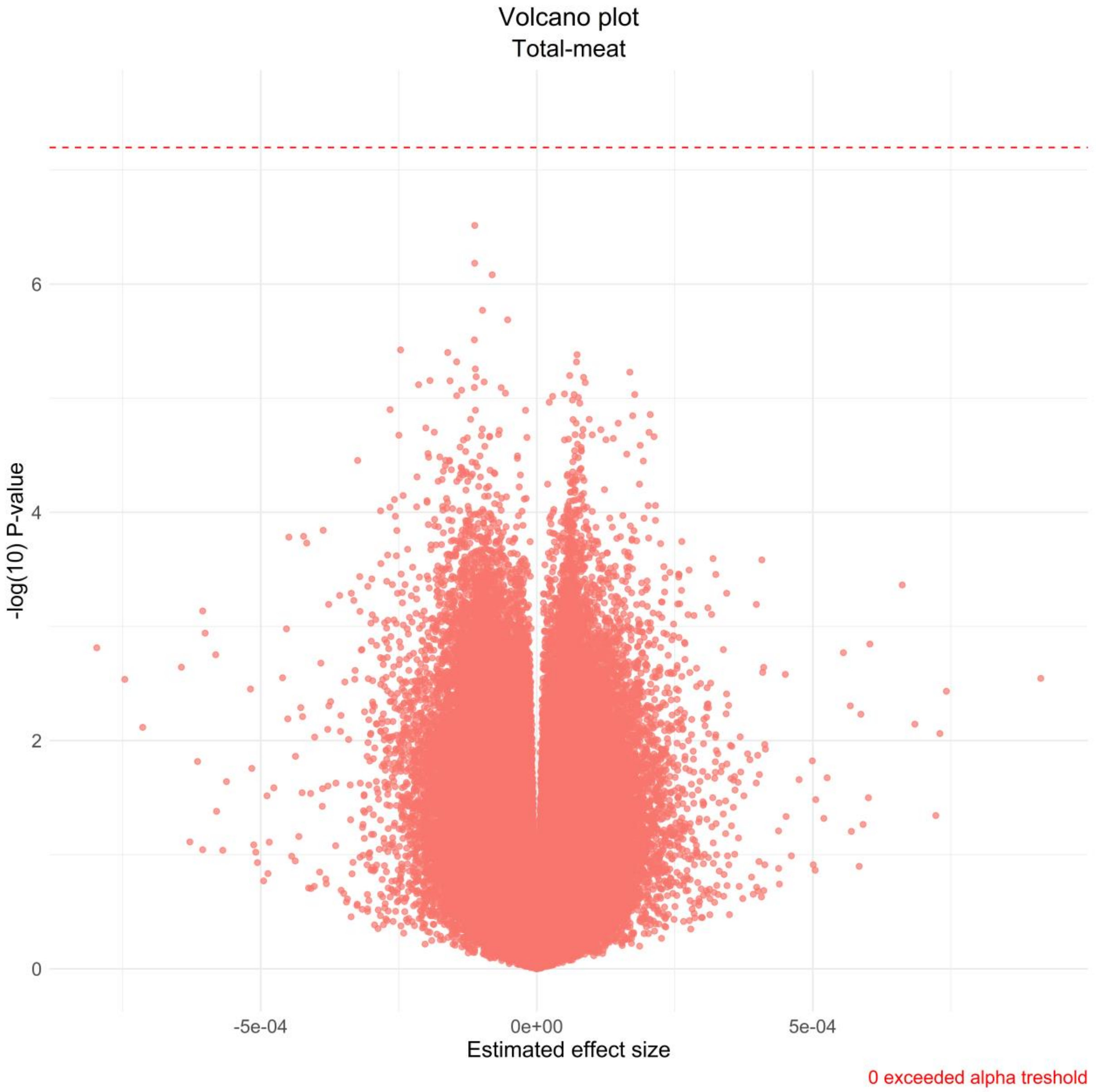

Volcano plot
Total-meat
-log(10) P-value
0
2
4
6
-5e-04
0e+00
5e-04
Estimated effect size
0 exceeded alpha treshold

Figure S34

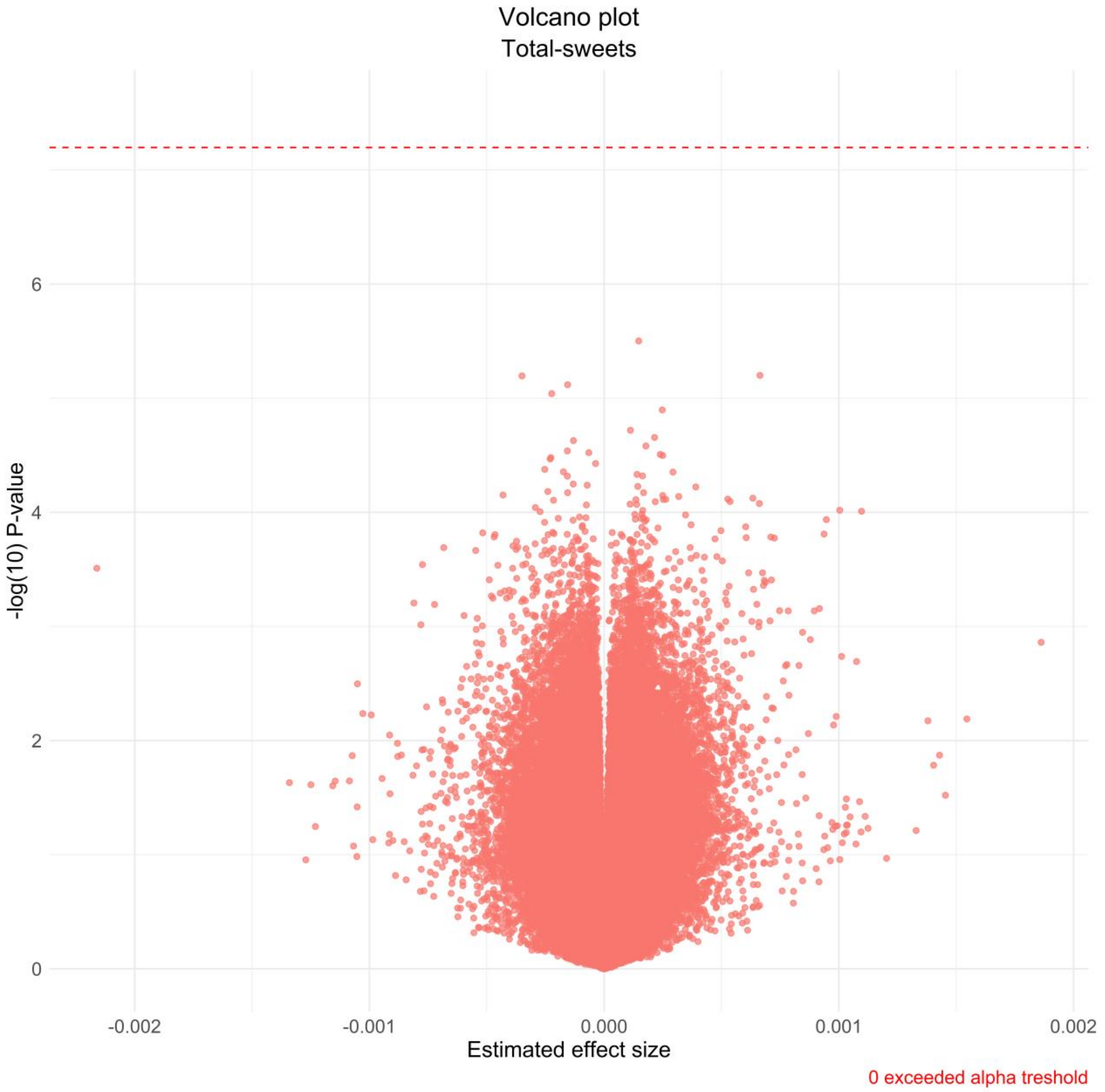

Volcano plot
Total-sweets
-log(10) P-value
0
2
4
6
-0.002
-0.001
0.000
0.001
0.002
Estimated effect size
0 exceeded alpha treshold

Figure S35

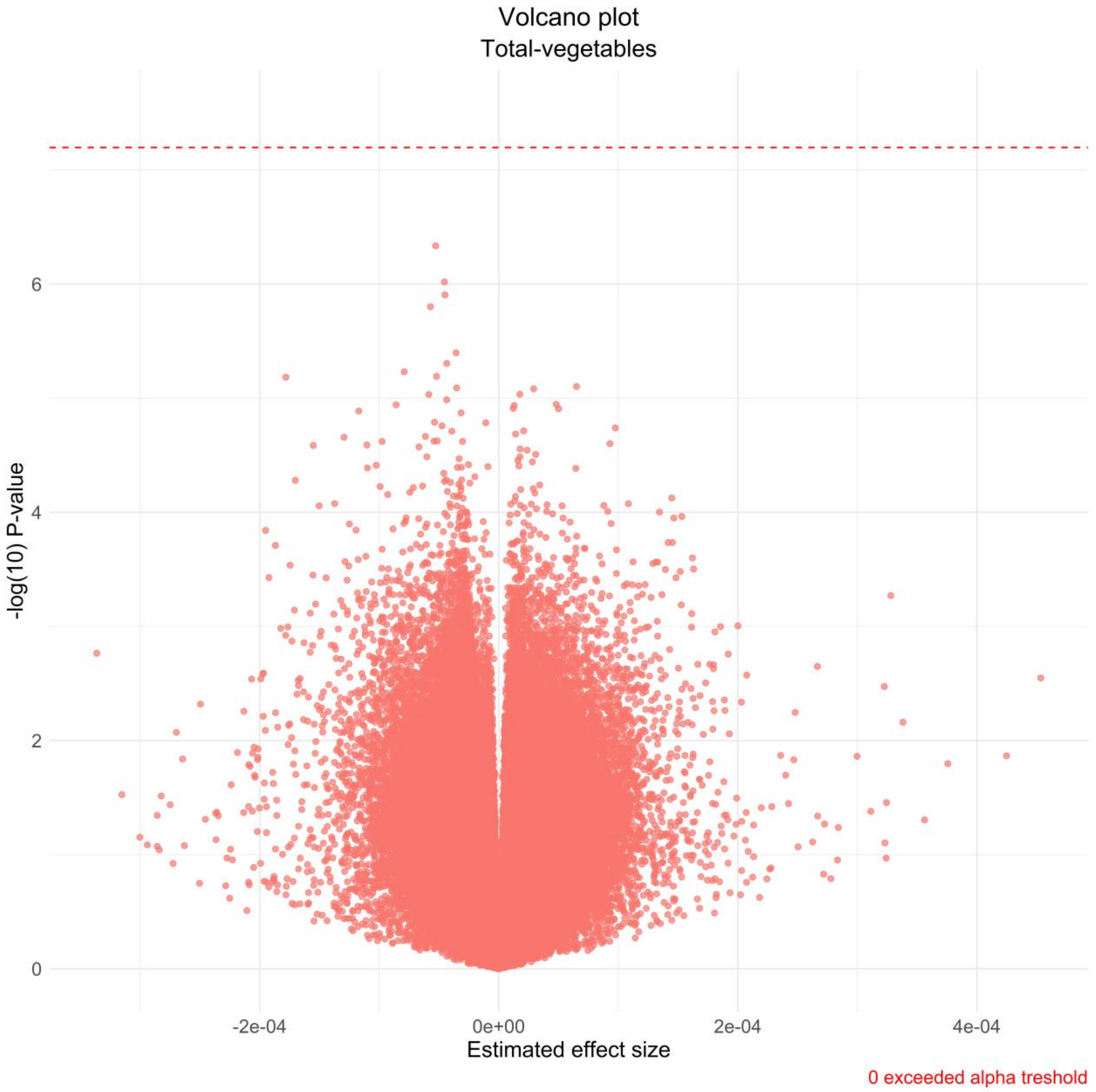

Volcano plot
Total-vegetables
-log(10) P-value
0
2
4
6
-2e-04
0e+00
2e-04
4e-04
Estimated effect size
0 exceeded alpha treshold

Figure S36

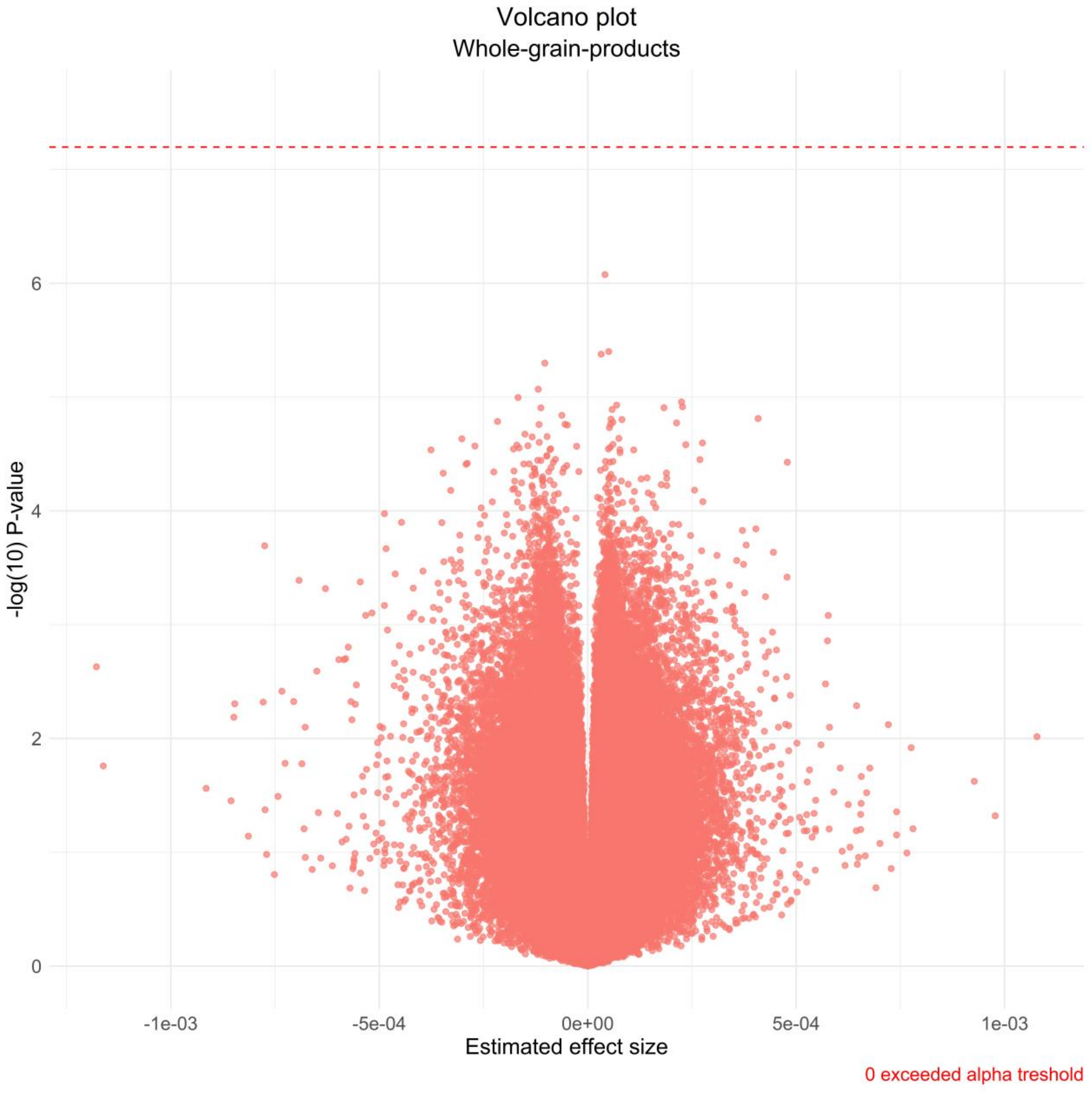

Volcano plot
Whole-grain-products
-log(10) P-value
6
4
2
0
-1e-03
-5e-04
0e+00
5e-04
1e-03
Estimated effect size
0 exceeded alpha treshold

Figure S37

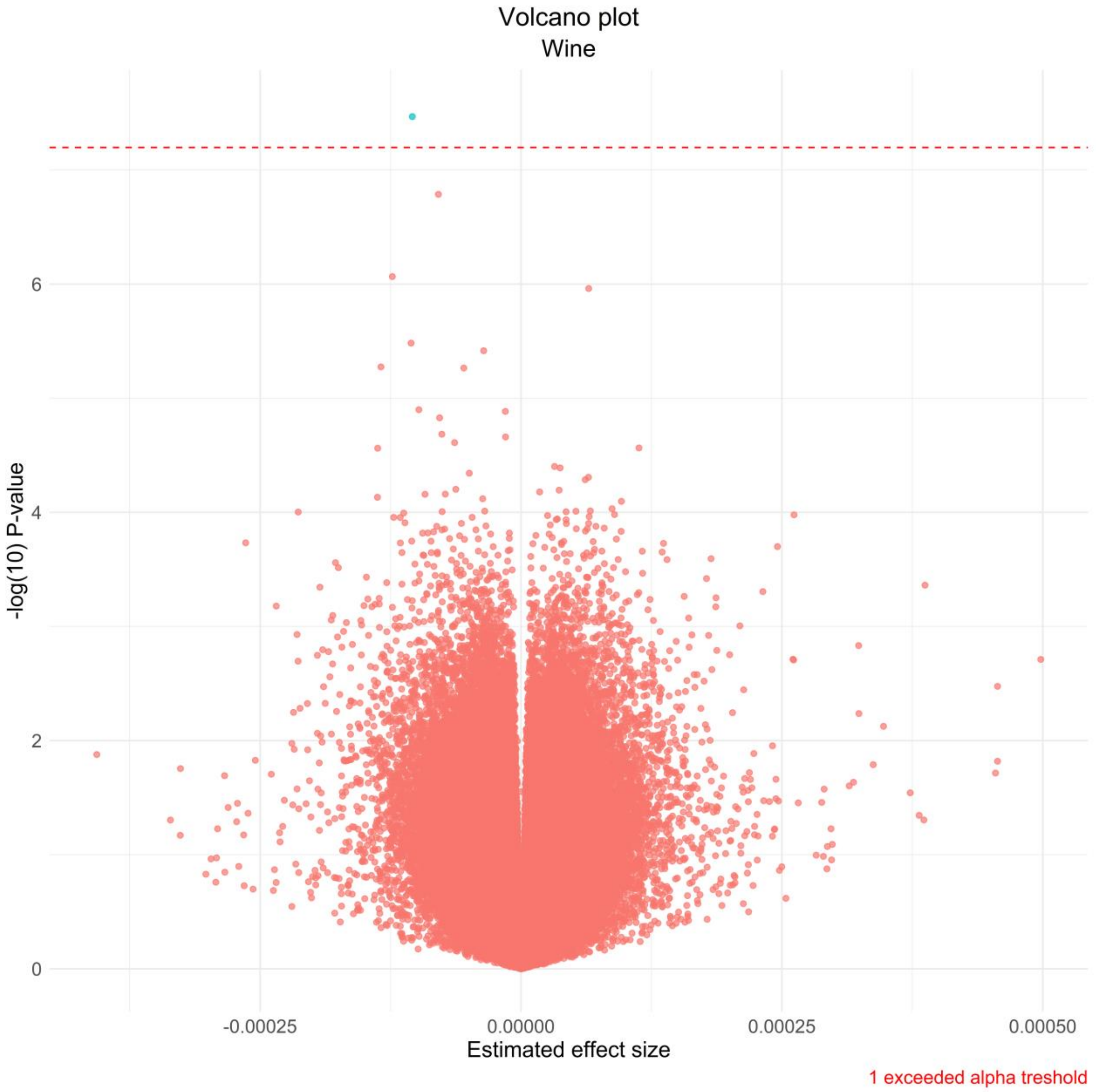

Volcano plot
Wine
-log(10) P-value
6
4
2
0
-0.00025
0.00000
0.00025
0.00050
Estimated effect size
1 exceeded alpha treshold

Figure S38

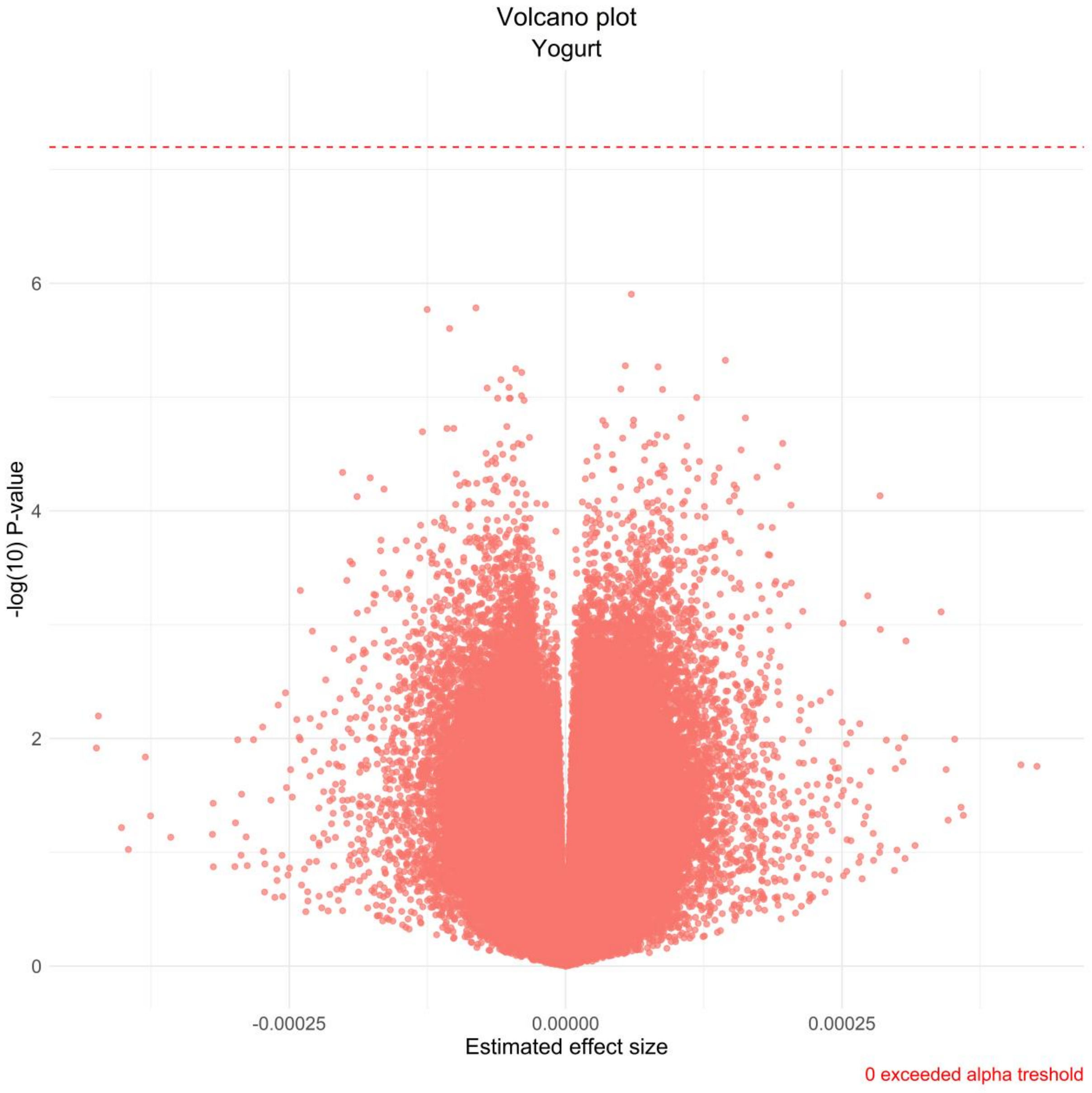

Volcano plot
Yogurt
-log(10) P-value
6
4
2
0
-0.00025
0.00000
0.00025
Estimated effect size
0 exceeded alpha treshold
