# Supplementary material for: Association between Usual Dietary Intake of Food Groups and DNA Methylation and Effect Modification by Metabotype in the KORA FF4 Cohort
Source: Life (Basel). 2022 Jul 15;12(7):1064. doi: 10.3390/life12071064 (PMC9318948; doi:10.3390/life12071064)
Supplement: Supplementary file 1 [file life-12-01064-s001.zip › life-1794131-supplementary/Suppl_tables/TableS4-Supplementary Material - legend.pdf]

Columns

probeID: CpG identifier

P_adj_fdr_mtype1;2;3: False-discovery rate adjusted p-value for exposure term or interaction term with metabotype 2 or 3

BETA_nutr*mtype2;3: Effect size of interaction term metabotype 2/3 in respective sheet

SE_nutr*mtype2;3: Standard error of interaction term metabotype 2/3 in respective sheet
