# Supplementary material for: Association between Usual Dietary Intake of Food Groups and DNA Methylation and Effect Modification by Metabotype in the KORA FF4 Cohort
Source: Life (Basel). 2022 Jul 15;12(7):1064. doi: 10.3390/life12071064 (PMC9318948; doi:10.3390/life12071064)
Supplement: Supplementary file 1 [file life-12-01064-s001.zip › life-1794131-supplementary/Suppl_tables/TablesS1-S3-legend_Supplementary Material.pdf]

Columns:

- probeID: Name of the probeID of the chip
- N_samp: Sample size available for the respective probeID
- BETA_nutr_in_mtype1: Effect size for metabotype 1
- SE_nutr_in_mtype1: Standard error for metabotype 1
- P_fdr_in_mtype1: P-value for metabotype 1 adjusted with FDR
- P_adj_bacon_fdr_mtype(x): P-value after addressing genomic inflation via the bacon package and adjusting with FDR
- P_interact_fdr_mtype(x): P-value of the interaction term with metabotype (x)
- Beta_mtype(x): Calculated marginal effect size for metabotype (x)
- Se_mtype(x): Calculated marginal standard error for metabotype (x)
- P_fdr_mtype(x): P-value calculated from the marginal effect size and standard error for metabotype (x) and adjusted with FDR with sample size for the adjustment being only probeIDs that were significant in the interaction term
- Mean_beta: mean methylation level of this probeID over all subjects included in the respective model
- SD_beta: standard deviation of the methylation level of this probeID over all subjects included in the respective model
- CHR: Chromsome
- UCSC_Ref_Gene_Name: Gene names from the UCSC database (see https://emea.support.illumina.com/bulletins/2016/08/infinium-methylationepic-manifest-column-headings.html?langsel=/fo/)
- UCSC_RefGene_Group: Gene region feature category describing the CpG position, from the UCSC. Features are listed in the same order as gene transcripts.
    - TSS200 = 0-200 bases upstream of the transcriptional start site (TSS)
    - TSS1500 = 200-1500 bases upstream of the TSS
    - Body = Between the ATG and stop codon; irrespective of the presence of introns, exons, TSS, or promoters.
    - 3'UTR = Between the stop codon and poly A signal
- Relation_to_UCSC_CpG_Island: The location of the CpG relative to the CpG island
    - Shore = 0-2kb from island
    - Shelf = 2-4kb from island
    - N = Upstream (5') of CpG island
    - S = Downstream (3') of CpG island
- Flag: Coding value – A number above zero means that this gene appeared multiple times across all exposures analyzed and shown in this sheet.

Coloring

- Green p-values – Statistically significant regarding the applied p-value adjustment and alpha threshold
- Coloring in the column UCSC_RefGene_Name means that this gene appeared multiple times across all exposures analyzed and shown in this sheet – (Basically a visualization of the flag column)
- Blue in bacon adjusted p-value columns mean that this CpG was still statistically significant (fdr < 0.1) after the genomic inflation correction
